# HIF-1–mediated PPME1 expression promotes breast cancer progression via AKT activation and **β**-catenin recruitment

**DOI:** 10.1172/JCI205415

**Published:** 2026-10-01

**Authors:** Yajing Lyu, Varen Talwar, Yongkang Yang, Si-Sim Kang, Shuyi Li, Shaima Salman, Daiana Drehmer, Yufeng Wang, Chelsey Chen, Vijay Ramu, Sujin Kim, Dylan Park, Tina Yi-Ting Huang, Emmanuel Datan, Dominic Dordai, Jonathan P. Schneck, Gregg L. Semenza

**Affiliations:** 1 Institute for Cell Engineering and; 2 Department of Genetic Medicine, Johns Hopkins University School of Medicine, Baltimore, Maryland, USA.; 3 Johns Hopkins University, Baltimore, Maryland, USA.; 4 Sidney Kimmel Comprehensive Cancer Center at Johns Hopkins, Baltimore, Maryland, USA.

**Keywords:** Genetics, Oncology, Breast cancer

## Abstract

Hypoxia-inducible factor 1 (HIF-1) orchestrates the transcriptional regulation of thousands of genes involved in breast cancer (BC) progression. Here, we identified protein phosphatase 2A (PP2A) methylesterase 1 (*PPME1*) as a critical HIF-1 target gene that drives oncogenic signaling under hypoxic conditions. In BC cells, HIF-1–dependent PPME1 expression caused inhibition of the PP2A catalytic subunit (PP2Ac), thereby diminishing PP2A activity, which led to AKT activation, phosphorylation of β-catenin, and its nuclear translocation. Nuclear β-catenin cooperates with HIF-1 to promote BC stem cell specification by activating transcription of the *NANOG* and *KLF4* genes, which encode pluripotency factors, and to drive immune evasion by activating transcription of *VEGFA*, which recruits and polarizes immunosuppressive tumor-associated macrophages and *ISG20*, which represses STAT1/IRF1-dependent expression of *CXCL10*, thereby impairing CD8^+^ T cell recruitment. In vivo, PPME1 knockdown altered the tumor immune microenvironment, enhanced antitumor immunity, and synergized with anti–CTLA-4 immunotherapy to enable complete tumor eradication. These findings establish PPME1 as a critical regulator linking hypoxia signaling, stemness, and immune evasion and highlight its potential as a BC therapeutic target in combination with immune checkpoint blockade.

## Abstract 2

Hypoxia-inducible factor 1 blocks protein phosphatase 2A activity in breast cancer cells, which increases AKT signaling, leading to cancer stem cell specification and immune evasion.

## Graphical abstract

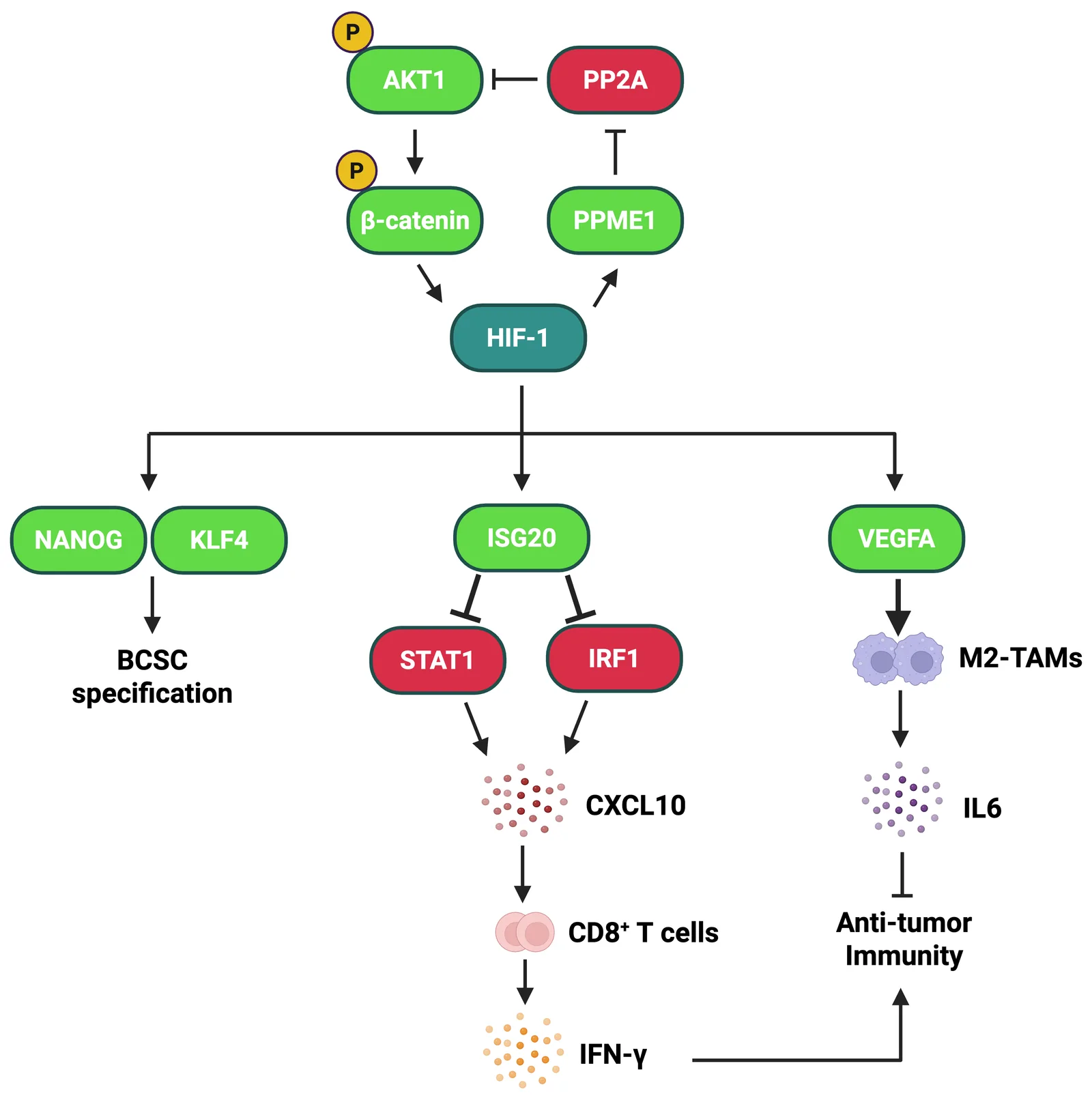

## Introduction

In 2025, over 300,000 women were diagnosed with breast cancer (BC) in the United States (https://seer.cancer.gov/statfacts/html/breast.html), making it the most common cancer diagnosis. BC is also projected to be the fourth-leading cause of cancer deaths, accounting for approximately 7% of all cancer-related mortality (https://seer.cancer.gov/statfacts/html/breast.html). Despite recent therapeutic advances, clinical outcomes remain suboptimal. A major contributor to treatment failure is the profound cellular heterogeneity within BC, particularly the presence of BC stem cells (BCSCs). These cells are thought to act as the clonogenic reservoir for new tumor growth (1). Beyond conventional treatments, immune checkpoint blockade (ICB) has become integrated into the treatment of triple-negative BC (TNBC), but only a small subset of patients has derived benefit due to multiple mechanisms by which cancers evade immune surveillance (2, 3). Therefore, it remains an urgent priority to deepen our understanding of the mechanisms underlying BCSC specification and immune evasion in order to identify novel therapeutic targets that can yield more potent and durable strategies for BC treatment.

The hypoxic tumor microenvironment is strongly associated with increased risk of BC metastasis and patient mortality (4). Intratumoral hypoxia is frequently observed in advanced BCs, with a median partial pressure of O_2_ of only approximately 10 mm Hg, compared with approximately 65 mm Hg in normal breast tissue (5). Hypoxia-inducible factors (HIFs) orchestrate the transcriptional response to low O_2_ levels (6). HIFs are heterodimeric transcription factors composed of an O_2_-regulated HIF-1α or HIF-2α subunit and a constitutively expressed HIF-1β subunit (7). Under normoxia, HIF-α subunits undergo O_2_-dependent prolyl hydroxylation, leading to von Hippel–Lindau protein (VHL)-mediated ubiquitination and subsequent proteasomal degradation (8). HIFs accumulate under hypoxic conditions, dimerize, bind to hypoxia-response elements (HREs) containing matches to the consensus sequence 5′-(A/G)CGTG-3′ in target genes, and recruit coactivators to drive transcription (9, 10). The resulting HIF-regulated transcriptional program contributes to every critical aspect of cancer progression, including cancer stem cell specification and immune evasion (11–13).

Protein phosphatase 2A (PP2A) is a serine/threonine phosphatase that plays a central role in regulating diverse cellular processes. PP2A is generally recognized as a tumor suppressor and is frequently inactivated in a wide range of cancers (14). Structurally, PP2A exists as a heterotrimeric holoenzyme, composed of a scaffolding subunit, a catalytic subunit (PP2Ac), and one of many regulatory B subunits. The composition and function of PP2A holoenzymes are tightly controlled by posttranslational modification of the carboxy terminal tail of PP2Ac. In particular, carboxymethylation at leucine 309 (Leu-309) is a critical modification. Leucine carboxylmethyltransferase-1 catalyzes this methylation, enabling stable interaction with certain regulatory subunits, such as the B55/PR55 family; conversely, PP2A methylesterase 1 (PPME1) reverses this modification, demethylating PP2Ac and thereby destabilizing holoenzyme formation by impairing binding of certain B subunits (15–17). In addition to its methylesterase activity, PPME1 can directly inhibit PP2Ac catalytic activity through active site occlusion mediated by the Met335 inhibitory loop. Upon binding PP2Ac, the Met335-containing loop of PPME1 inserts into the PP2Ac active site and displaces two catalytic Mn^2+^ ions that are required to activate the bridging water nucleophile for phosphate ester hydrolysis, rendering PP2Ac catalytically inactive independently of Leu-309 methylation status (18). PP2A holoenzymes assembled with distinct B regulatory subunits suppress multiple major oncogenic signaling cascades, including PI3K-AKT, Wnt/β-catenin, mTOR, and MAPK/ERK. Consequently, dysregulation of PP2A activity leads to the coordinate hyperactivation of these cascades, thereby driving cancer progression (19–21).

Tumor progression relies on crosstalk among multiple signaling pathways. AKT (protein kinase B) has been shown to phosphorylate β-catenin at serine 552 (Ser-552), which promotes its translocation into the nucleus (22). Nuclear-localized β-catenin interacts with transcription factors to enhance the expression of downstream target genes, thereby driving tumor invasion (23). PPME1, by antagonizing PP2A activity, plays a pivotal role in modulating these pathways. Indeed, PPME1 overexpression has been shown to correlate with enhanced proliferation and invasive phenotypes in endometrial adenocarcinoma, where it sustains ERK and AKT activation by inhibiting PP2A activity (24, 25). β-Catenin has also been reported to mediate immunosuppression through exclusion of cytotoxic T cells from some cancers (26). However, the molecular mechanisms through which PPME1 contributes to BC progression remain poorly defined.

In the current study, we identified the *PPME1* gene as a direct transcriptional target of HIF-1 and demonstrated its critical role in BCSC specification and immune evasion. Specifically, HIF-1–driven upregulation of PPME1 inhibits PP2Ac activity, leading to AKT activation, enhanced β-catenin phosphorylation, and its nuclear translocation. Nuclear β-catenin cooperates with HIF-1 to activate transcription of the genes encoding Nanog homeobox (NANOG) and Krueppel-like factor 4 (KLF4), thereby promoting BCSC specification, as well as IFN-stimulated gene 20 (ISG20) and VEGFA, which drive immune evasion by modulating tumor-infiltrating immune cell populations. In vivo, *PPME1* knockdown altered the tumor immune microenvironment, enhanced antitumor immunity, and synergized with anti–CTLA-4 therapy. Our results establish *PPME1* as a critical regulator linking hypoxia signaling, BCSCs, and immune evasion and highlight its potential as a BC therapeutic target in combination with ICB.

## Results

### PPME1 expression is mediated by HIF-1 in hypoxic BC cells.

Analysis of several recently published RNA-seq datasets from our lab revealed that PPME1 mRNA expression was significantly elevated in hypoxic BC cells (13, 27, 28). Consistently, hypoxia induced PPME1 expression at both the mRNA (Figure 1A and Supplemental Figure 1A; supplemental material available online with this article; https://doi.org/10.1172/JCI205415DS1) and protein (Figure 1B and Supplemental Figure 1B) levels in human estrogen receptor–positive (ER^+^) and progesterone receptor–positive (PR^+^) MCF-7 and T-47D cells; human epidermal growth factor receptor 2–positive (HER2^+^) HCC1954 cells; human triple-positive BT-474 cells; human triple-negative MDA-MB-231, SUM159, and SUM149 cells; mouse triple-positive EO771 cells; mouse triple-negative EMT6 and 4T1 cells; and the mouse brain metastatic TNBC subclone 4T1-BR5.

Analysis of The Cancer Genome Atlas (TCGA) BC dataset revealed that patients with high PPME1 expression exhibited significantly increased expression of a 17-gene HIF signature (ANGPTL4, PDK1, VEGFA, LDHA, PGK1, HK2, CA9, CXCR3, L1CAM, BNIP3, PLOD1, P4HA1, P4HA2, NARF, ISG20, PADI4, and ITGB3) compared with patients with low PPME1 expression (Figure 1C). This association suggests that HIFs might regulate PPME1 expression in human BCs.

To determine the HIF dependency of PPME1 induction, we established MDA-MB-231 and MCF7 subclones stably transduced with a lentiviral vector encoding a nontargeting control (NTC) shRNA or shRNA(s) targeting HIF-1α, HIF-2α, or both (double knockdown [DKD]) and exposed the subclones to 1% O_2_. HIF-1α-knockdown (KD) and DKD, but not HIF-2α–KD, abrogated the hypoxic induction of PPME1 mRNA and protein in MDA-MB-231 (Figure 1, D and E) and MCF7 (Figure 1, F and G) cells. In contrast, expression of *RPL13A*, a gene that is not HIF regulated, was not affected by exposure to hypoxia or by HIF-KD in MDA-MB-231 or MCF7 (Supplemental Figure 1, C and D) cells. Treatment of BALB/c mice bearing EMT6 tumors with dual HIF-1/2 inhibitors that induce HIF-1α/2α protein degradation abrogated PPME1 protein expression in tumor tissue (Supplemental Figure 1E). Thus, both in vitro and in vivo data indicate that PPME1 expression is regulated by HIF-1.

To examine whether HIF-1 directly binds to the *PPME1* gene for transcriptional activation in SUM159 cells, we performed ChIP using an anti–HIF-1α antibody followed by DNA sequencing (ChIP-seq) and identified a HIF-1α binding peak in the *PPME1* gene. Within this peak, we found 2 overlapping sequences that match the HIF-1 consensus binding motif, 5′-(A/G)CGTG-3′, located 92 bp downstream of the transcription start site (Figure 1H). We exposed MDA-MB-231 and MCF7 cells to 1% O_2_ and performed ChIP followed by quantitative real-time PCR (ChIP-qPCR) assays. Binding of HIF-1α and HIF-1β, but not HIF-2α, to the *PPME1* gene, but not the *RPL13A* gene, was induced by hypoxia in MDA-MB-231 (Figure 1I and Supplemental Figure 1F) and MCF7 cells (Figure 1J and Supplemental Figure 1G). These results indicate that *PPME1* is bound by HIF-1 but not HIF-2. Together, these findings demonstrate that *PPME1* is a direct transcriptional target of HIF-1 in BC cells.

### PPME1 promotes nuclear localization of β-catenin in BC cells by enhancing AKT activity.

PPME1 negatively regulates PP2A by demethylation of Leu-309 in the catalytic subunit (29, 30). In MDA-MB-231 cells, hypoxia-induced PPME1 expression led to demethylation at Leu-309 of PP2Ac, whereas PPME1-KD abrogated hypoxia-induced Leu-309 demethylation, without markedly affecting total PP2Ac protein levels (Figure 2A). Hypoxia significantly decreased PP2A catalytic activity, as measured by liberation of phosphate from a model substrate, and this effect was lost in PPME1-KD subclones (Figure 2B).

We next analyzed AKT signaling, which plays a key role in BC progression and is negatively regulated by PP2A (31, 32). Hypoxia has been reported to induce AKT kinase activation and phosphorylation of serine 552 (Ser-552), and β-catenin has been shown to be mediated by AKT (23). In MDA-MB-231 subclones, hypoxia-induced phosphorylation of AKT at Ser-473 and Thr-308 and phosphorylation of β-catenin at Ser-552 were attenuated in PPME1-KD subclones, with no changes in total AKT or β-catenin protein levels (Figure 2C), indicating that PPME1 is required for hypoxia-mediated activation of AKT → β-catenin signaling in BC cells. Gene set enrichment analysis (GSEA) of RNA-seq data from patients with BC (TCGA) revealed enrichment of phosphatidylinositol 3-kinase–AKT (PI3K-AKT) signaling in tumors with the highest PPME1 expression compared with tumors with the lowest expression, supporting the role of PPME1 in activating AKT signaling (Supplemental Figure 1H). To validate the correlation between hypoxia and PI3K-AKT signaling in patients with BC, we examined pathway activity using gene expression signatures (33, 34) in TCGA data (35). We observed a significant positive association between hypoxia and PIK3CA pathway scores (Supplemental Figure 1I), indicating that hypoxia is associated with increased PI3K-AKT signaling in human BC tissue. Hypoxia-induced activation of the AKT → β-catenin axis was also observed in the nontumorigenic human mammary epithelial cell line MCF10A, indicating that this response is not restricted to malignant cells (Supplemental Figure 2A). We analyzed a published proteomic dataset comprising BC specimens from 105 patients (36) and found that PPME1 protein expression was positively correlated with β-catenin phosphorylation at Ser-552 (Supplemental Figure 2B). Cytoplasmic and nuclear fractionation in MDA-MB-231 subclones revealed that hypoxia increased nuclear Ser-552–phosphorylated β-catenin abundance, whereas PPME1 KD markedly attenuated this hypoxia-induced nuclear accumulation (Supplemental Figure 2C). Together, these data establish that PPME1 is required for hypoxia-induced nuclear translocation of β-catenin through Ser-552 phosphorylation and confirm the clinical relevance of this axis in BC.

To determine the HIF dependency of AKT → β-catenin signaling, we examined AKT and β-catenin phosphorylation in MDA-MB-231 subclones. HIF-1α, but not HIF-2α, was required for hypoxia-induced phosphorylation of AKT and β-catenin (Figure 2D). This pattern mirrors the hypoxia-induced activation of *PPME1* by HIF-1 and indicates that HIF-1 → PPME1 transcriptional activation leads to AKT → β-catenin pathway activation.

To investigate the involvement of PP2A in this pathway, we treated MDA-MB-231 cells with PP2A activator SMAP-2 or PP2A inhibitor LB-100. Treatment with SMAP-2 mimicked the effect of PPME1-KD, abrogating hypoxia-induced phosphorylation of AKT and β-catenin (Figure 2E). In contrast, treatment with LB-100 produced the opposite effect, enhancing the phosphorylation of AKT and β-catenin (Figure 2F). No changes were observed with respect to total AKT or β-catenin protein levels across all conditions. Because neither compound affected PP2A protein levels, these findings suggest that PP2A activity is critical for PPME1-mediated activation of AKT → β-catenin signaling under hypoxia in BC cells.

To determine which regulatory function of PPME1 drives hypoxia-induced AKT → β-catenin activation, we employed two point mutants with distinct biochemical properties: S156A, which abolishes methylesterase activity while retaining PP2A inhibition; and M335D, which retains methylesterase activity but fails to inhibit PP2A (37). Reexpression of S156A in PPME1-KD cells fully recapitulated wild-type PPME1, restoring AKT and β-catenin phosphorylation and hypoxia-induced ISG20 and KLF4 expression, whereas M335D failed to restore any of these outputs (Supplemental Figure 2D). This dissociation establishes that the tumor-promoting AKT → β-catenin axis is governed by the PP2Ac active site inhibition function of PPME1, independent of its demethylase activity.

Taken together, our findings demonstrate that HIF-1–mediated PPME1 expression leads to PP2Ac inhibition, thereby decreasing its phosphatase activity toward AKT at Ser-473 and Thr-308, which results in enhanced AKT signaling, promoting β-catenin nuclear localization via phosphorylation at Ser-552 (Figure 2G).

### PPME1 is required for hypoxia-induced BCSC specification.

HIF-1 plays a critical role in BCSC specification by inducing the expression (13) and activity (14) of pluripotency factors under hypoxic conditions. To investigate how HIF-1–mediated PPME1 expression contributes to BCSC enrichment, we analyzed TCGA data. We observed a significant positive correlation between the expression of PPME1 mRNA and a 20-gene BCSC signature (38) (Figure 3A). Culturing BC cells under nonadherent conditions induces the formation of mammospheres, which have been shown to be enriched in BCSCs (39). We performed mammosphere formation assays using NTC and PPME1-KD subclones of MDA-MB-231 and MCF7 cells to assess secondary mammosphere formation, which was initiated after dissociating primary mammospheres and represents a more rigorous measure of BCSC specification (40). The hypoxia-induced enhancement of mammosphere formation observed in MDA-MB-231 NTC subclones persisted through the second generation over 14 days after removal from the hypoxic environment. In contrast, this effect was abolished in PPME1-KD subclones (Figure 3, B and C). Similar results were observed in MCF7 cells (Supplemental Figure 3, A and B). Furthermore, we compared the expression of PPME1 with expression of NANOG and KLF4, which are required for BCSC specification (11, 41–44). Both mRNA and protein levels were markedly induced in MDA-MB-231 mammospheres as compared with adherent cells (Figure 3, D and E). Similar results were observed in MCF7 cells (Supplemental Figure 3, C and D). These findings demonstrate that PPME1 is required for hypoxia-induced NANOG and KLF4 expression and BCSC specification.

Aldehyde dehydrogenase (ALDH) is a marker of BCSCs, and its catalytic activity regulates tumor stemness (45). Hypoxia induces expansion of the BCSC population, as reflected by an increased percentage of ALDH^+^ cells (46). ALDH^+^ cells in NTC and PPME1-KD subclones of MDA-MB-231 and MCF7 cells exposed to 20% or 1% O_2_ were quantified by flow cytometry (gating shown in Supplemental Figure 3, E and F). PPME1-KD significantly decreased the ALDH^+^ population after hypoxic exposure of MDA-MB-231 (Figure 3F) or MCF7 (Supplemental Figure 3G) cells. Both mRNA and protein levels of NANOG and KLF4 were also decreased in PPME1-KD as compared with NTC subclones of MDA-MB-231 (Figure 3, G–I) and MCF7 (Supplemental Figure 3, H–J). Taken together, these results indicate that PPME1 promotes hypoxia-induced BCSC specification by stimulating the expression of NANOG and KLF4.

### PPME1 regulates BC tumor initiation and growth.

To investigate the role of PPME1 in regulating the tumor-initiating capacity of BC cells — a key functional property of BCSCs in vivo (47, 48) — we orthotopically injected 1,000 NTC or PPME1-KD subclone cells into the mammary fat pad of SCID mice. Under these circumstances, BCSCs are limiting for tumor formation. Following a long-term observation period of 87 days, tumors developed in 9 of 10 mice injected with NTC cells, whereas only 5 of 20 mice injected with either of 2 independent PPME1-KD subclones developed tumors (Figure 4A), indicating that PPME1 is essential for efficient tumor initiation in vivo. We began measurement of tumor volume on day 70 when all tumors could be reliably assessed. At this time point, tumors in the NTC group appeared larger than those in the PPME1-KD group. Tumor growth was monitored for an additional 17 days. In those mice in which tumor formation occurred, PPME1-KD markedly suppressed tumor growth, as evidenced by decreased tumor volume over time (Figure 4B) and decreased mean tumor weight at the endpoint (Figure 4C). These findings indicate that PPME1 is critical for both BC tumor initiation and growth.

To validate the involvement of NANOG and KLF4 in PPME1-mediated BCSC enrichment in vivo, we extracted mRNA and protein from tumor tissues and analyzed the expression of PPME1, NANOG, and KLF4. The sustained depletion of PPME1 at both mRNA and protein levels confirmed long-term KD efficiency in vivo. Consistently, the expression of NANOG and KLF4 was markedly decreased in PPME1-KD tumors at both the mRNA and protein levels (Figure 4, D–G). Our laboratory has previously established through gain- and loss-of-function studies that NANOG and KLF4 are essential and sufficient mediators of hypoxia-induced BCSC specification downstream of HIF-1 (11, 49, 50). Thus, PPME1 regulates BCSC specification and tumor initiation capacity in association with regulation of NANOG and KLF4 expression in vivo.

### Increased PPME1 expression correlates with adverse clinical outcome.

We analyzed publicly available datasets and found that levels of both PPME1 mRNA (Supplemental Figure 4, A and B) and protein (Supplemental Figure 4C) were elevated in BC compared with normal breast tissue. PPME1 mRNA levels were higher in all stages (Supplemental Figure 4D) and in all subtypes (Supplemental Figure 4E) of human BC. These clinical data are consistent with the similar phenotypes and regulatory mechanisms that we observed in cell lines derived from both TNBC (MDA-MB-231) and ER^+^/PR^+^ BC (MCF7). Kaplan-Meier analysis of 2 GEO datasets revealed that elevated PPME1 expression was significantly associated with decreased overall survival (Supplemental Figure 4F) and relapse-free survival (Supplemental Figure 4G) of patients with BC.

### β-Catenin is required for hypoxia-induced and HIF-1–dependent BCSC enrichment.

β-Catenin plays a dynamic role in tumorigenesis and has been reported to enhance HIF-1–mediated transcription (51). Given our finding that PPME1 promotes β-catenin nuclear translocation via AKT signaling, we sought to investigate the role of nuclear β-catenin in hypoxic BC cells. β-Catenin KD (CTNNB1-KD) markedly decreased the expression of NANOG and KLF4 in MDA-MB-231 (Figure 5, A–C) and MCF7 (Supplemental Figure 5, A–C) cells, indicating that nuclear β-catenin is required for the hypoxia-induced upregulation of NANOG and KLF4. CTNNB1-KD also decreased the percentage of ALDH^+^ cells under hypoxic conditions, to a degree comparable to HIF1A-KD (Figure 5D), further supporting the role of β-catenin in hypoxia-induced BCSC specification.

To validate regulation of the AKT–β-catenin pathway by PP2A in BCSCs, we performed the ALDH assay in MDA-MB-231 cells treated with PP2A activator SMAP-2, PI3K inhibitor LY294002, or β-catenin inhibitor MSAB. Each treatment significantly decreased the ALDH^+^ cell population under hypoxia (Figure 5E), validating the mechanism by which PPME1 mediates hypoxia-induced BCSC specification. Treatment with MSAB, which triggers β-catenin degradation, abolished the hypoxia-induced expression of NANOG and KLF4 protein in both MDA-MB-231 (Supplemental Figure 5D) and MCF7 (Supplemental Figure 5E) cells.

To investigate the sufficiency of β-catenin for BCSC specification, we engineered β-catenin overexpression (β-catenin–OE) in MDA-MB-231 cells. β-Catenin–OE slightly increased the ALDH^+^ cell population under nonhypoxic conditions but significantly enhanced the hypoxia-induced expansion of ALDH^+^ cells, and this enhancement was abrogated by HIF1A-KD (Figure 5F). Furthermore, cooverexpression of β-catenin and HIF-1α under nonhypoxic conditions also led to a notable increase in ALDH^+^ cells (Supplemental Figure 5F), indicating that β-catenin amplifies the effect of HIF-1 on hypoxia-induced BCSC specification.

### HIF-1, PPME1, and β-catenin are required for broad transcriptional response to hypoxia.

To establish a genome-wide role for PPME1 and β-catenin in HIF-1–mediated transcription, we performed RNA-seq. NTC, HIF1A-KD, PPME1-KD, and CTNNB1-KD subclones of SUM159 TNBC cells were exposed to 20% or 1% O_2_ for 24 hours, and 3 biological replicates for each condition were subjected to RNA-seq. In response to hypoxia, expression of 2,685 RNAs was increased and expression of 2,322 RNAs was decreased >1.5-fold with a FDR < 0.05 in the NTC subclone (Supplemental Figure 6A). Enrichment analysis of these 2 gene sets revealed distinct signaling patterns under hypoxia. Among hypoxia-induced RNAs, hypoxia and TNF-α signaling ranked at the top (Supplemental Figure 6B). In contrast, hypoxia-repressed RNAs were most enriched for IFN-α and IFN-γ response (Supplemental Figure 6C).

Heatmap analysis of the 4 subclones revealed that the transcriptional responses to hypoxia mediated by HIF-1 were largely dependent on PPME1 and β-catenin (Figure 6A). Venn analysis of the hypoxia-induced genes that were HIF-1α–dependent, PPME1-dependent, and β-catenin–dependent revealed a high degree of overlap (Figure 6B).

To further explore the role of PPME1 and β-catenin in HIF-1–dependent transcription in BC cells, we analyzed the expression of a HIF-1–dependent reporter gene. Cells were cotransfected with p2.1, which contains the *ENO1* HRE upstream of a basal SV40 promoter and firefly luciferase (FLuc) coding sequences, and pSV-RL, which contains the SV40 promoter alone upstream of Renilla luciferase (RLuc) coding sequences, such that the FLuc/RLuc ratio is a measure of HIF-1 transcriptional activity. Hypoxia increased HIF-1–dependent transcription in NTC cells but KD of HIF-1α, PPME1 or β-catenin expression blocked hypoxia-induced transcription (Figure 6C).

We also analyzed the effects of PPME1 and β-catenin depletion on HIF-1α transactivation domain (TAD) function. Cells were cotransfected with pGalA, which encodes a fusion protein containing the GAL4 DNA-binding domain fused to HIF-1α residues 531–826; reporter plasmid pG5-E1b-FLuc, which contains 5 GAL4 binding sites upstream of a basal adenoviral *E1b* promoter and FLuc coding sequences; and control reporter pSV-RL. Hypoxia induced GalA-dependent transcription in NTC but not in PPME1-KD or CTNNB1-KD cells (Figure 6D). No transcriptional activity was observed in cells transfected with pGalO, which encodes the GAL4 DNA-binding domain alone (Figure 6D). Together, these results suggest that deficiency of PPME1 or β-catenin impairs HIF-1 transcriptional activity due to loss of TAD function. This finding is in agreement with a previous report in SW480 colorectal cancer cells, in which CTNNB1-KD significantly reduced hypoxia-induced HIF-1–dependent reporter activity (51). Taken together, these results demonstrate that HIF-1, PPME1, and β-catenin are all required for a broad transcriptional response to hypoxia in BC cells.

### PPME1 and β-catenin repress the STAT1/IRF1/CXCL10 axis via ISG20.

Because IFN-α and IFN-γ responses were identified as the top 2 enriched signatures among hypoxia-repressed RNAs (Supplemental Figure 6C), and a recent study from our lab demonstrated that ISG20 mediates immune evasion in hypoxic BC cells (12), we reanalyzed our RNA-seq data and were intrigued to find that *ISG20* expression depends on HIF-1, PPME1, and β-catenin (Supplemental Figure 6D). Next, we investigated how PPME1 and β-catenin regulate *ISG20* expression in hypoxic BC cells. ChIP-qPCR assays using antibodies against HIF-1α, HIF-1β, and p300 in MDA-MB-231 cells demonstrated that both PPME1 and β-catenin were required for HIF-1α, HIF-1β, and p300 binding to the *ISG20* HRE (Figure 7, A–C). Immunoblot analysis of HIF-1α and HIF-1β protein levels at the same time point showed no marked changes across all subclones (Figure 7D), confirming that the loss of HIF-1 and p300 binding to the *ISG20* HRE was not due to protein degradation.

HIF-1 has been shown to increase transcription initiation of target genes, which is measured by the presence of RNA polymerase II (RPII) phosphorylated at Ser-5 of the C-terminal domain (RPII-S5P), and to stimulate the release of paused RPII, which is measured by phosphorylation at Ser-2 of the C-terminal domain (RPII-S2P) (13). Hypoxia-induced occupancy of the *ISG2*0 HRE by RPII–S5P and RPII–S2P in NTC cells was impaired in HIF1A-KD, PPME1-KD, and CTNNB1-KD subclones (Supplemental Figure 7A). These results indicate that, as in the case of HIF-1α, both PPME1 and β-catenin are required for efficient induction of transcription initiation and release of paused RPII for productive transcript elongation at the *ISG20* gene under hypoxic conditions.

Hypoxia has been reported to suppress CXCL10 expression in various cancer cell types (52–55). STAT1 and IRF1, transcription factors that cooperatively activate *CXCL10* transcription (56, 57), are also known to be downregulated in hypoxia in a HIF-dependent manner (58). ISG20 is an exonuclease that degrades STAT1 and IRF1 mRNAs, thereby inhibiting CXCL10 expression in hypoxic BC cells. These findings provide direct causal evidence that ISG20 functions as an effector mediating hypoxia-induced repression of the STAT1/IRF1/CXCL10 axis (12). Thus, we analyzed the expression of ISG20, STAT1, IRF1, and CXCL10 in MDA-MB-231 cells. Hypoxia-induced ISG20 expression was markedly attenuated in HIF1A-KD, PPME1-KD, and CTNNB1-KD cells, whereas the hypoxia-mediated repression of STAT1, IRF1, and CXCL10 was reversed in these KD subclones, at both the mRNA (Figure 7, E–H) and protein (Figure 7I) levels. A similar mRNA expression pattern was observed in EMT6 cells (Supplemental Figure 7, B–E). Collectively, these results demonstrate that both PPME1 and β-catenin are required for hypoxia-mediated repression of the STAT1/IRF1/CXCL10 axis via HIF-1–mediated transcriptional activation of *ISG20*.

### PPME1 → β-catenin → HIF-1 → ISG20 signaling mediates immune evasion.

We hypothesized that PPME1 contributes to hypoxia-induced suppression of antitumor immunity. To test this hypothesis, we established orthotopic EMT6 breast tumors in syngeneic immunocompetent BALB/c mice using NTC, PPME1-KD, or CTNNB1-KD subclones. Depletion of PPME1 markedly suppressed tumor growth (Figure 8A) and decreased tumor weight at the endpoint (Figure 8B). CTNNB1-KD cells formed very small tumors and failed to sustain further growth (Figure 8A). This observation is consistent with those in previous reports showing that loss of β-catenin suppresses tumor growth in BC and other tumor types (59–63). Due to the limited amount of CTNNB1-KD tumor tissue, subsequent experimental analyses were performed with NTC and PPME1-KD tumors only. Sustained depletion of PPME1 mRNA confirmed the long-term KD efficiency in vivo (Figure 8C). Tumor tissues were analyzed by multispectral flow cytometry to quantify CD8^+^ T cells (Supplemental Figure 8A), which mediate antitumor immunity, and M2-type tumor-associated macrophages (M2-TAMs) (Supplemental Figure 8B), which mediate immunosuppression. The proportion of intratumoral CD8^+^ T cells was significantly increased (Figure 8D), whereas M2-TAMs were markedly decreased (Figure 8E) in PPME1-KD tumors. These results indicate that PPME1 mediates BC immune evasion by impairing CD8^+^ T cell recruitment and enhancing the M2-TAM population. CD8^+^ T cells produce IFN-γ, an effector cytokine that plays a critical role in the cell-mediated immune response against intracellular pathogens and tumor cells (64, 65). We observed increased IFN-γ expression at both the mRNA (Figure 8F) and protein (Figure 8G) levels in PPME1-KD tumors. CXCL10 mediates cell recruitment by binding to the chemokine receptor CXCR3 on the surface of T cells. CXCL10 expression was increased at both the mRNA (Figure 8H) and protein (Figure 8I) levels in PPME1-KD tumors. These results indicate that PPME1 mediates T cell exclusion by suppressing CXCL10 expression.

ISG20 mRNA levels were significantly decreased in PPME1-KD tumors (Figure 8J), whereas STAT1 (Figure 8K) and IRF1 (Figure 8L) mRNA levels were increased. Consistent changes were observed at the protein level (Figure 8M). In our recent study, ISG20-KD restored STAT1, IRF1, and CXCL10 expression, as well as intratumoral CD8^+^ T cell and NK cell recruitment in orthotopic breast tumor models, establishing ISG20 as a direct driver of immune exclusion (12). Collectively, these findings indicate that PPME1-dependent ISG20 expression suppresses STAT1/IRF1-mediated CXCL10 expression, thereby blocking the recruitment of CD8^+^ T cells.

In addition to CXCL10, we also analyzed the expression of VEGFA and IL6, which mutually reinforce M2-TAM recruitment and immunosuppressive functions in multiple cancers (66–70). Consistent with the decreased number of M2-TAMs, VEGFA (Figure 8, N and O) and IL6 (Figure 8, P and Q) expression was decreased in PPME1-KD tumors. Since *VEGFA* is a well-known HIF-1 target gene, we further examined its dependency on PPME1 and β-catenin and found that hypoxic induction of VEGFA was dependent on HIF-1α, PPME1, and β-catenin expression in EMT6 cells (Supplemental Figure 9A). ELISA further validated these results at the secreted protein level (Supplemental Figure 9B). However, IL6 mRNA was undetectable in EMT6 cells according to our previous RNA-seq analysis (12). These data suggest that the reduced level of IL6 production in tumors is likely attributable to a decreased population of M2-TAMs, which are known to express IL6, rather than altered expression in tumor cells. Together, these results suggest that PPME1 KD modulates tumor-infiltrating activated T cells and M2-TAMs by altering the expression of key chemokines (CXCL10 and VEGFA), thereby reshaping the balance between antitumor (IFN-γ) and protumor (IL6) cytokines in the tumor microenvironment to enhance antitumor immunity.

We further investigated the PPME1–AKT–β-catenin axis, previously analyzed in cell culture, in EMT6 tumors. Consistent with our in vitro findings, PPME1-KD led to decreased phosphorylation of AKT at Ser-473 and Thr-308, and decreased phosphorylation of β-catenin at Ser-552, without major changes in the total levels of each protein (Supplemental Figure 10A). Unexpectedly, HIF-1α protein levels were decreased in PPME1-KD tumors, suggesting that long-term PPME1 depletion might impair HIF-1α protein synthesis or stability.

### PPME1-KD suppresses tumor growth via CXCL10-driven CD8^+^ T cell recruitment.

To dissect the immune mechanisms underlying the tumor-suppressive effects of PPME1 KD, we orthotopically implanted BALB/c mice with EMT6 subclones expressing NTC or PPME1 shRNA. NTC tumor-bearing mice received intraperitoneal injections of anti-VEGFA or IgG isotype control antibody, while shPPME1 tumor-bearing mice were treated with anti-CXCL10, anti-CD8α, or IgG isotype control antibody (Figure 9A). Anti-VEGFA treatment significantly reduced tumor growth in NTC mice relative to that in IgG controls, whereas neutralization of CXCL10 or depletion of CD8^+^ T cells in shPPME1 mice both substantially rescued tumor growth (Figure 9B). Consistent with this, shPPME1+IgG tumors exhibited a marked increase in intratumoral CD8^+^ T cell abundance relative to NTC+IgG; anti-CXCL10 treatment significantly reduced this infiltration, and anti-CD8α treatment nearly abolished it, confirming that CXCL10 is required for CD8^+^ T cell recruitment in PPME1-deficient tumors (Figure 9C). Analysis of M2-TAMs revealed that anti-VEGFA treatment significantly decreased the percentage of M2-TAMs in NTC tumors, indicating that VEGFA drives M2-TAM polarization or infiltration (Figure 9D). Collectively, these findings demonstrate that PPME1-KD suppresses tumor growth through CXCL10-driven CD8^+^ T cell recruitment, while VEGFA sustains an immunosuppressive microenvironment through M2-TAM enrichment.

### PPME1-KD enhances the efficacy of anti-CTLA-4 immunotherapy.

To investigate whether PPME1 silencing can enhance the response to ICB, we established orthotopic EMT6 breast tumors in immunocompetent BALB/c mice using NTC and PPME1-KD subclones. Once palpable tumors developed, mice were treated with either anti–CTLA-4 antibody or isotype control IgG (Figure 10A). Compared with NTC tumors treated with isotype control antibody, anti–CTLA-4 treatment of NTC tumors and PPME1-KD tumors treated with isotype control antibody showed significantly decreased tumor growth; however, anti–CTLA-4 treatment in the PPME1-KD group resulted in complete tumor eradication in all of the mice (Figure 10, B–F). Mice with eradicated tumors were given a secondary tumor challenge on day 117 but no further treatment. By the final endpoint (day 237), no tumor recurrence was observed in any of the mice, indicating durable antitumor immunity. Thus, PPME1 KD enhances the therapeutic efficacy of anti–CTLA-4 ICB.

## Discussion

Intratumoral hypoxia is one of the most common features of advanced cancers and activates a wide array of signaling pathways critical for tumor progression (7). AKT is a kinase that regulates diverse cellular processes to promote cancer progression (71). Hypoxia-induced AKT activation has been reported across multiple cancer types, and these studies have placed AKT both upstream and downstream of HIF-1 (72, 73). We identified a significant positive correlation between hypoxia signaling and PI3K pathway activation in patients with BC and validated hypoxia-induced AKT phosphorylation in BC cell lines. The activity of AKT is determined by the balance between stimulatory kinases such as PI3K and inhibitory phosphatases such as PP2A. Cancer research has focused primarily on gain-of-function mutations that activate stimulatory kinases. Here, we demonstrate that hypoxia is sufficient to increase AKT activity in the absence of any activating mutation, and HIF-1–driven *PPME1* expression is essential for hypoxia-induced AKT phosphorylation, leading to downstream phosphorylation of β-catenin, through the suppression of PP2A activity. PPME1 regulates PP2Ac through two distinct functions: Leu-309 demethylation, which preferentially destabilizes methylation-dependent B55-containing holoenzymes whose substrates are enriched in cell cycle regulators (74, 75), and direct PP2Ac active site inhibition, which broadly suppresses all PP2A holoenzymes, including methylation-independent B56 complexes responsible for AKT dephosphorylation (76). Using S156A PPME1 (demethylase-dead, active site inhibition intact) and M335D PPME1 (demethylase-active, active site inhibition disrupted), we showed that only the active site inhibition arm restores AKT–β-catenin signaling in PPME1-KD cells, establishing that direct suppression of PP2A activity is the critical mechanism driving HIF-1–dependent AKT–β-catenin activation in hypoxic BC. This mechanism highlights a noncanonical regulatory axis in which AKT signaling is activated not by growth factor binding to receptor tyrosine kinases at the cell membrane (77) but by HIF-1 in hypoxic BC cells.

The Wnt signaling pathway is a critical regulator of cell-fate determination, tissue homeostasis, and tumorigenesis (78). Its activation leads to the association of β-catenin with nuclear TCF–leukocyte enhancer factor proteins, resulting in transcriptional activation of downstream target genes (78, 79). Interestingly, a study investigating the interplay between hypoxia and β-catenin signaling in colorectal tumorigenesis found that hypoxia inhibited β-catenin–TCF-4 transcriptional activity. β-Catenin was shown to interact with HIF-1α and enhance HIF-1 transcriptional activity in colorectal cancer cells (51). In our BC model, we demonstrated that β-catenin is essential for BCSC specification by maintaining NANOG and KLF4 expression under both nonhypoxic and hypoxic conditions. β-Catenin–mediated BCSC specification in hypoxia was HIF-1–dependent, consistent with previous studies (11), which demonstrated that HIF-1 orchestrates hypoxia-induced BCSC specification by regulating the expression of pluripotency factors required for self-renewal.

Our RNA-seq data revealed that the transcriptional responses to hypoxia mediated by HIF-1α were largely dependent on PPME1 and β-catenin. Mechanistically, luciferase reporter assays of HIF-1 transcriptional activity demonstrated that both PPME1 and β-catenin are required for HIF-1–mediated transactivation, suggesting that β-catenin functions as a HIF-1 cofactor in regulating a large subset of hypoxia-inducible genes. PPME1, by controlling β-catenin nuclear translocation, cooperates with β-catenin to regulate these HIF-1 target genes. ChIP-qPCR assays revealed that PPME1 and β-catenin expression were necessary for stable occupancy of HREs by HIF-1 and the coactivator p300 in hypoxic BC cells.

Several HIF-induced transcriptional repressors have been identified that enable cancer cell adaptation to the hypoxic microenvironment (43, 80–82). Notably, hypoxia-repressed transcriptional signatures derived in vitro have been reported to hold prognostic value in BC (83). In this study, we validated STAT1 and IRF1 as HIF-1α–, PPME1-, and β-catenin–dependent hypoxia-repressed genes and demonstrated that their repression leads to decreased expression of CXCL10. Here, a key insight came from a recent study in our laboratory, which revealed that binding of STAT1 and IRF1 to the *Cxcl10* gene is lost in hypoxic BC cells, due to HIF-dependent expression of ISG20, an exonuclease that degrades STAT1 and IRF1 mRNA (12). We identified ISG20 among the HIF-1α–, PPME1-, and β-catenin–dependent hypoxia-induced RNAs, thereby providing a molecular mechanism linking PPME1 expression to CXCL10 repression and the exclusion of CD8^+^ T cells from the tumor immune microenvironment. To functionally validate this axis in vivo, we showed that neutralization of CXCL10 or depletion of CD8^+^ T cells in shPPME1 tumor-bearing mice fully rescued tumor growth and abolished intratumoral CD8^+^ T cell infiltration, establishing that CXCL10-driven CD8^+^ T cell recruitment is the dominant immune mechanism underlying the tumor-suppressive effect of PPME1-KD.

It has become increasingly clear that hypoxia shapes and induces specific macrophage phenotypes that promote tumor malignancy (84, 85). TAMs are preferentially recruited to hypoxic tumor regions through chemoattractants secreted by hypoxic tumor and stromal cells, among which VEGFA plays a central role (85). Together with other tumor microenvironmental signals, VEGFA reprograms TAMs toward a tumor-supportive phenotype (86) by suppressing M1-associated markers (CXCL10, CCR7, and IL12) while inducing M2-associated markers (CD206, CD163, and CCL17) (68). We observed decreased numbers of M2-TAMs in PPME1-KD tumors, accompanied by corresponding decreases in VEGFA mRNA and protein within the tumor tissue. We further validated VEGFA expression in human and mouse BC cell lines and found that it is robustly induced by hypoxia in a HIF-1α–, PPME1-, and β-catenin–dependent manner. Consistent with this, VEGFA blockade in NTC tumor-bearing mice significantly reduced both tumor growth and intratumoral M2-TAMs, functionally supporting a role for VEGFA in PPME1-dependent regulation of M2-TAMs. In a feedforward loop, M2-TAMs themselves secrete VEGFA to reinforce tumor angiogenesis (87) and further TAM recruitment/polarization. Notably, CD8^+^ T cell depletion restored M2-TAM abundance in shPPME1 tumors, revealing a reciprocal relationship in which CXCL10-recruited CD8^+^ T cells may suppress M2-TAM accumulation; however, the underlying mechanism remains to be fully elucidated, with IFN-γ–mediated macrophage repolarization representing one plausible pathway (88).

M2-TAM–derived IL6 inhibits drug-induced apoptosis and thereby increases chemoresistance in colorectal cancer by activating the IL6R/STAT3 signaling pathway (89). Conversely, tumor-derived IL6 promotes tumor invasion by stimulating M2-TAM polarization and TNF-α secretion (90). IL6 mediates immunosuppression by negatively regulating the antitumor activity of natural killer and CD8^+^ T cells (91). We observed a marked decrease in IL6 mRNA and protein levels in PPME1-KD tumors. However, IL6 mRNA expression was very low, even under hypoxic conditions, in EMT6 cells cultured in vitro (12). These findings suggest that, in the orthotopic EMT6 model, immune cells such as M2-TAMs are the predominant source of IL6. PPME1 KD decreases the percentage of M2-TAMs in tumors by inhibiting VEGFA expression, thereby substantially decreasing IL6 production. Thus, PPME1 represses STAT1/IRF1→CXCL10 signaling to block recruitment of CD8^+^ T cells producing cytotoxic IFN-γ and activates VEGFA expression to increase recruitment/polarization of M2-TAMs producing immunosuppressive IL6.

PPME1 has emerged as an attractive cancer therapeutic target. ABL127 is a selective covalent PPME1 inhibitor that engages both the Ser156 methylesterase active site and disrupts the physical PPME1-PP2Ac interaction (92). That ABL127 targets the correct functional interface yet still fails in vivo implicates pharmacological rather than target-selection limitations, underscoring the need for next-generation PPME1-PP2Ac interface disruptors with improved drug-like properties (25). PPME1-KD altered the expression of a broad battery of genes to impair BCSC specification and restore antitumor immunity and a durable, complete response to anti–CTLA-4 antibody treatment, effects that have also been observed after treatment with HIF inhibitors (93, 94). Our findings nominate HIF inhibition as a comprehensive therapeutic strategy by establishing HIF-1 as the transcriptional driver of PPME1 expression. Treatment with HIF inhibitor 1.21S9N led to complete tumor eradication across mouse models of breast, colorectal, melanoma, and prostate cancer when combined with ICB, with oral bioavailability and a favorable safety profile (95), providing compelling and robust therapeutic validation of the HIF-1–PPME1–immune evasion axis characterized here.

## Methods

### Sex as a biological variable.

Our study exclusively examined female mice because the vast majority of BC occurs in females.

### Cell culture.

The sources of cell lines and culture media are shown in Supplemental Table 1. Cells were cultured at 37°C in a 5% CO_2_, 95% air incubator. The culture media recipes and hypoxic culture conditions are provided in the Supplemental Methods.

### Lentiviral transduction.

Lentiviral vectors encoding shRNA targeting HIF-1α, HIF-2α, PPME1, and β-catenin were purchased from Millipore Sigma (clone IDs are listed in Supplemental Table 6). Lentiviral packaging and transduction procedures are described in the Supplemental Methods.

### RT-qPCR.

Total RNA was isolated using TRIzol (Invitrogen), reverse-transcribed using the High-Capacity RNA-to-cDNA Kit (Thermo Fisher Scientific), and qPCR was performed using SYBR Green (Bio-Rad) and the CFX96 Real-Time PCR detection system (Bio-Rad). The expression (*E*) of each target mRNA relative to 18S rRNA was calculated based on the cycle threshold (Ct) as *E* = 2^–Δ(ΔCt)^, in which ΔCt = Ct (target) – Ct (18S), and Δ (ΔCt) = ΔCt (test sample) – ΔCt (control sample). PCR primer sequences are shown in Supplemental Table 2.

### Immunoblot assays.

Whole cell lysates were prepared in modified RIPA buffer with protease inhibitor cocktail. Equal amounts of lysate were fractionated by SDS-PAGE and transferred onto nitrocellulose membranes. The blots were incubated with primary antibodies (Supplemental Table 3) overnight at 4°C and with secondary antibodies at room temperature for 1 hour, followed by signal detection using ECL Plus (Cytiva, catalog RPN2236).

### ChIP-qPCR.

Cells were incubated at 20% or 1% O_2_ for 16 hours, cross-linked in 1% formaldehyde for 10 minutes at 37°C, quenched in 0.125 M glycine for 5 minutes at 37°C, and lysed with SDS lysis buffer (1% SDS, 10 mM EDTA, 50 mM Tris [pH 8.1]). The detailed ChIP procedure is provided in the Supplemental Methods. Antibodies are listed in Supplemental Table 4, and primer sequences are listed in Supplemental Table 5.

### PP2A phosphatase assays.

Cells were resuspended in 1 mL ice-cold PBS, and a 200 μL aliquot of the suspension was reserved for cell counting. The remaining cells were pelleted and resuspended in 300 μL phosphatase extraction buffer. The cell suspension was sonicated for 10 seconds on ice, followed by centrifugation at 2,000*g* for 5 minutes at 4°C. The supernatants were collected, and phosphatase activity assays were performed with a phospho-AKT1 peptide substrate (K-R-pT-I-R-R). Released free phosphate was quantified as absorbance at 625 nm using the kit’s detection reagent (Ser/Thr Phosphatase Assay Kit from Upstate Biotechnology, catalog 17-127). PP2A activity was normalized to cell number.

### Mammosphere assay.

Adherent BC cells were trypsinized and dissociated into single-cell suspensions and then seeded in 6-well ultralow attachment plates at 5,000 cells/mL in Complete MammoCult Medium (STEMCELL Technologies). Primary mammospheres were cultured for 7 days under normoxic conditions, collected, and dissociated into single-cell suspensions using trypsin. Cells were reseeded under identical conditions for an additional 7 days. Secondary mammospheres were visualized and photographed using a phase-contrast microscope (Olympus), and spheres with a diameter ≥70 μm were quantified.

### ALDH assay.

The ALDEFLUOR assay (STEMCELL Technologies) was performed according to the manufacturer’s instructions, as described in the Supplemental Methods.

### RNA-seq.

SUM159 subclones were seeded into 6-well plates in 3 biological replicates and exposed to 20% or 1% O_2_ for 24 hours. Total RNA was isolated using TRIzol and treated with DNase. The library preparation, sequencing, and analysis procedures are described in the Supplemental Methods.

### Animal studies.

All animals were housed in a pathogen-free vivarium facility at the Johns Hopkins University School of Medicine. For all animal studies, 6- to 8-week-old female mice were used. The cancer cell injection procedure and tumor measurement methods are described in the Supplemental Methods.

### Tumor-infiltrating immune cell analysis.

EMT6 tumors were minced, digested with 1 mg/mL type I collagenase at 37°C for 30 minutes, and filtered through 70 μm cell strainers. Red blood cells were lysed using ammonium-chloride-potassium lysis buffer. The cells were rinsed with cold FACS buffer (1% BSA in PBS), resuspended in Fc Block (BD Biosciences), and stained with indicated antibodies (Supplemental Table 7) to capture immune cell populations that were quantified by flow cytometry (96). FlowJo software (BD Biosciences) was used for data analysis.

### ELISA.

Tumor tissues were homogenized in ice-cold PBS supplemented with 1% Triton X-100 and protease inhibitors. Detailed procedures are provided in the Supplemental Methods. Information about the ELISA kits is provided in Supplemental Table 7.

### Inhibitors, reagents, kits, and treatment antibodies.

The inhibitors, reagents, and kits used in this study are summarized in Supplemental Tables 8 and 9. Treatment antibodies used for in vivo experiments are described in Supplemental Table 10.

### Statistics.

All data are expressed as mean ± SD. Differences between 2 or multiple groups were analyzed by Student’s 2-tailed *t* test or 1-way ANOVA (Figure 9, C and D) or 2-way ANOVA for all others, respectively, and *P* values of less than 0.05 were considered significant. Details of the statistical analysis are provided in the Supplemental Methods.

### Study approval.

Protocols were approved by the Johns Hopkins University Animal Care and Use Committee and were in accordance with the NIH’s *Guide for the Care and Use of Laboratory Animals* (National Academies Press, 2011).

### Data availability.

RNA-seq and ChIP-seq data were deposited in the NCBI Gene Expression Omnibus database under accession numbers GSE314395 and GSE287168, respectively. Public data acquisition and analysis procedures are provided in the Supplemental Methods. All other data that support the findings of this study are presented in the main paper and supplemental materials. Values for all data points in graphs are reported in the Supporting Data Values file. Raw immunoblots are presented in the full unedited blot file.

## Author contributions

YL and GLS conceived and designed research studies and experiments. YL performed most of the experiments and acquired data, with help from VT, YY, SSK, SL, SK, SS, D Drehmer, VR, YW, DP, TYTH, and ED. CC performed ChIP-seq and data analyses. YL, VT, and YY performed RNA-seq data and public dataset analyses. VT, YY, SS, TYTH, and D Dordai helped with animal experiments. SSK, SL, SK, and JPS helped with flow cytometry. YL and GLS interpreted data and wrote the manuscript. GLS supervised the study. All authors reviewed and approved the manuscript.

## Conflict of interest

GLS is a cofounder of and holds equity in HIF Therapeutics Inc. This arrangement has been reviewed and approved by Johns Hopkins University in accordance with its conflict-of-interest policies.

## Funding support

- Armstrong Family Foundation grant to GLS.

## Supplementary Material

Supplemental data

Unedited blot and gel images

Supporting data values

## Figures and Tables

**Figure 1:**
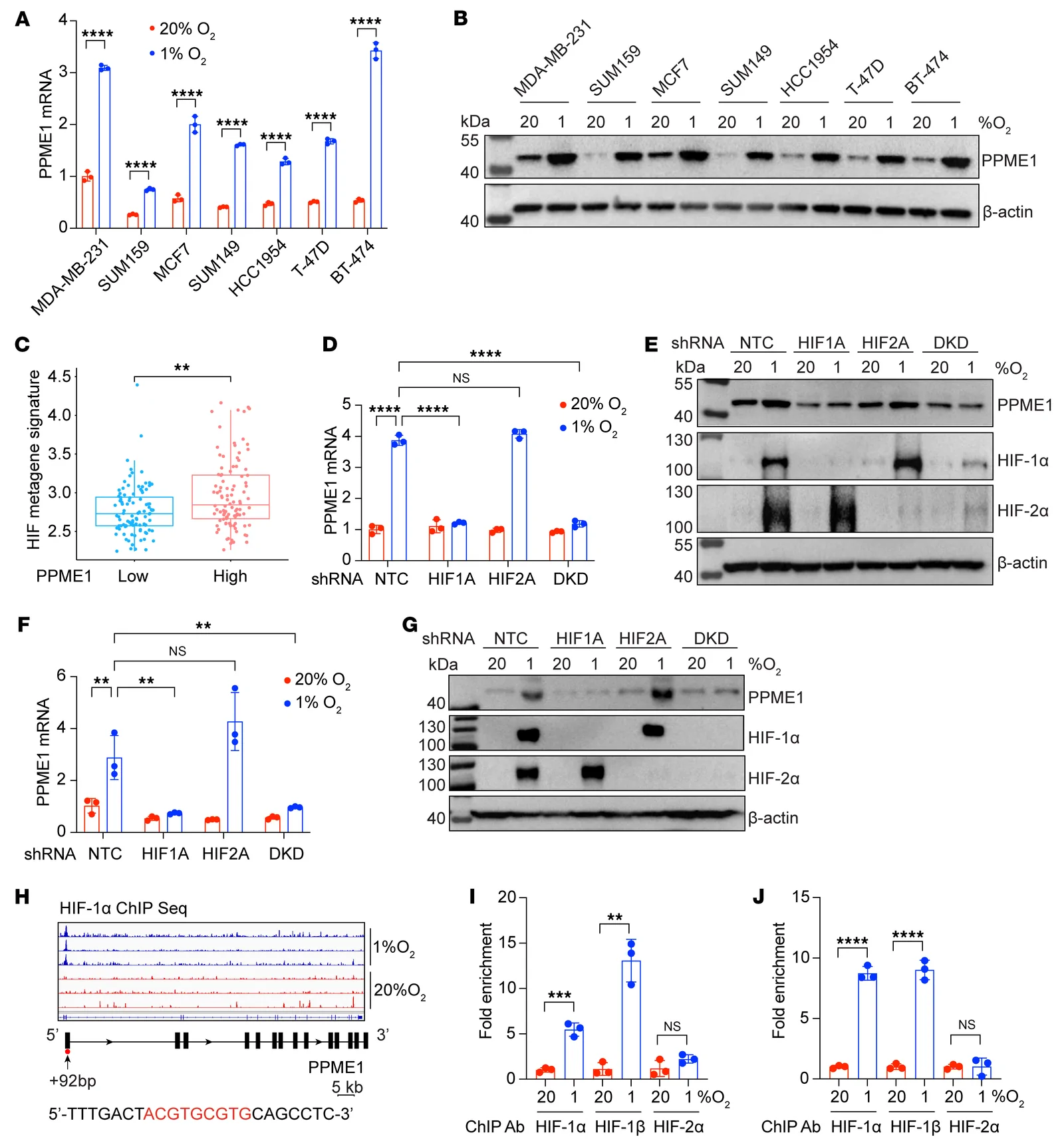
PPME1 expression is mediated by HIF-1 in hypoxic BC cells. (**A**) Human BC cell lines were exposed to 20% or 1% O_2_ for 24 hours, and PPME1 mRNA expression was analyzed by RT-qPCR. For each cell line, the expression of PPME1 mRNA was normalized to the mean result for MDA-MB-231 cells at 20% O_2_. ****P < 0.0001, 2-way ANOVA with Tukey’s multiple comparisons test. (**B**) BC cell lines were exposed to 20% or 1% O_2_ for 48 hours, whole cell lysates were prepared, and immunoblot assays were performed. (**C**) Comparison of the expression levels of 17 mRNAs encoded by HIF target genes (HIF metagene signature) in BCs that ranked in the top 100 (high) or bottom 100 (low) in TCGA database with respect to PPME1 mRNA expression. **P < 0.01, Wilcoxon test. (**D**–**G**) MDA-MB-231 (**D** and **E**) and MCF7 (**F** and **G**) subclones, which stably expressed a nontargeting control shRNA (NTC) or shRNA targeting HIF-1α, HIF-2α, or both HIF-1α and HIF-2α (DKD), were exposed to 20% or 1% O_2_ for 24 or 48 hours, followed by RT-qPCR (**D** and **F**) or immunoblot assay (**E** and **G**), respectively. **P < 0.01, ****P < 0.0001; 2-way ANOVA with Tukey’s multiple comparisons test. (**H**) ChIP-seq analysis of SUM159 cells revealed overlapping matches to the HIF consensus binding site 5′-(A/G)CGTG-3′ (highlighted in red) under the HIF-1α peak in the *PPME1* gene at +92 bp relative to the transcription start site using the Integrative Genomics Viewer genome browser. (**I** and **J**) MDA-MB-231 (**I**) and MCF7 (**J**) cells were exposed to 20% or 1% O_2_ for 16 hours, and ChIP assays were performed using antibodies against HIF-1α, HIF-1β, or HIF-2α. Primers flanking the candidate HIF binding site were used for qPCR, and results were normalized to the mean result at 20% O_2_. **P < 0.01, ***P < 0.001, ****P < 0.0001, 2-tailed Student’s *t* test. In all bar graphs, data are presented as mean ± SD (*n* = 3).

**Figure 2:**
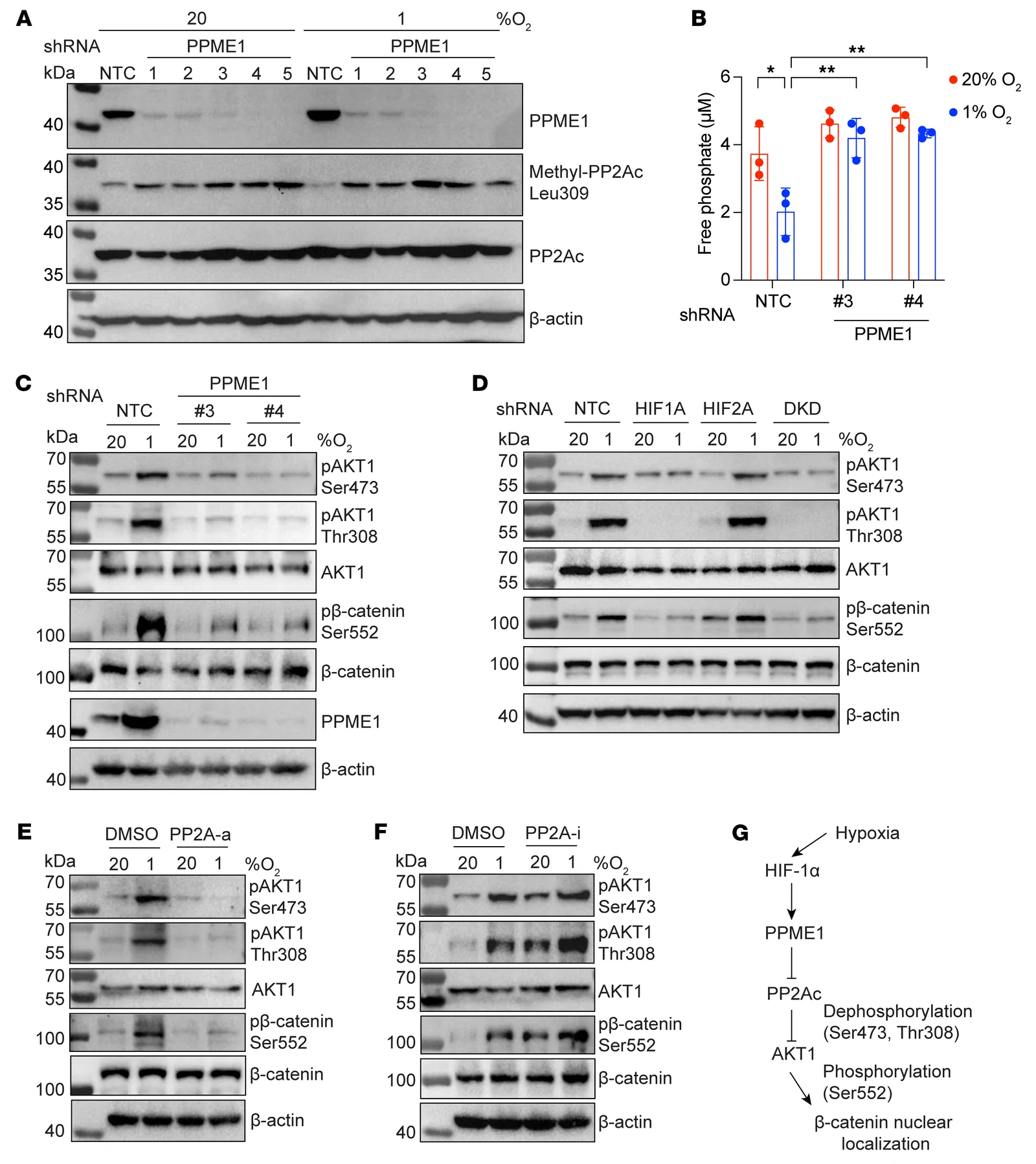
PPME1 promotes nuclear localization of β-catenin in BC cells by enhancing AKT activation through PP2Ac inhibition. (**A**) MDA-MB-231 subclones, which stably expressed an NTC shRNA or 1 of 5 shRNAs targeting PPME1 were exposed to 20% or 1% O_2_ for 48 hours, followed by immunoblot assays. (**B** and **C**) Subclones of MDA-MB-231 cells that expressed NTC or PPME1 shRNA (#3 or #4) were exposed to 20% or 1% O_2_ for 48 hours, followed by the malachite green Ser/Thr phosphatase assay (**B**) and immunoblot assays (**C**). Phosphate concentration was normalized to cell number. In the bar graph, data are presented as mean ± SD (*n* = 3); *P < 0.05, **P < 0.01, 2-way ANOVA with Tukey’s multiple comparisons test. (**D**) MDA-MB-231 subclones were exposed to 20% or 1% O_2_ for 48 hours, followed by immunoblot assays. (**E** and **F**) MDA-MB-231 cells were treated with vehicle (DMSO), PP2A activator (PP2A-a) SMAP-2 (10 μM; **E**), or PP2A inhibitor (PP2A-i) LB-100 (5 μM; **F**) and exposed to 20% or 1% O_2_ for 48 hours, followed by immunoblot assays. (**G**) The schematic illustrates the mechanism by which HIF-1 induces β-catenin nuclear translocation through PPME1 under hypoxic conditions.

**Figure 3:**
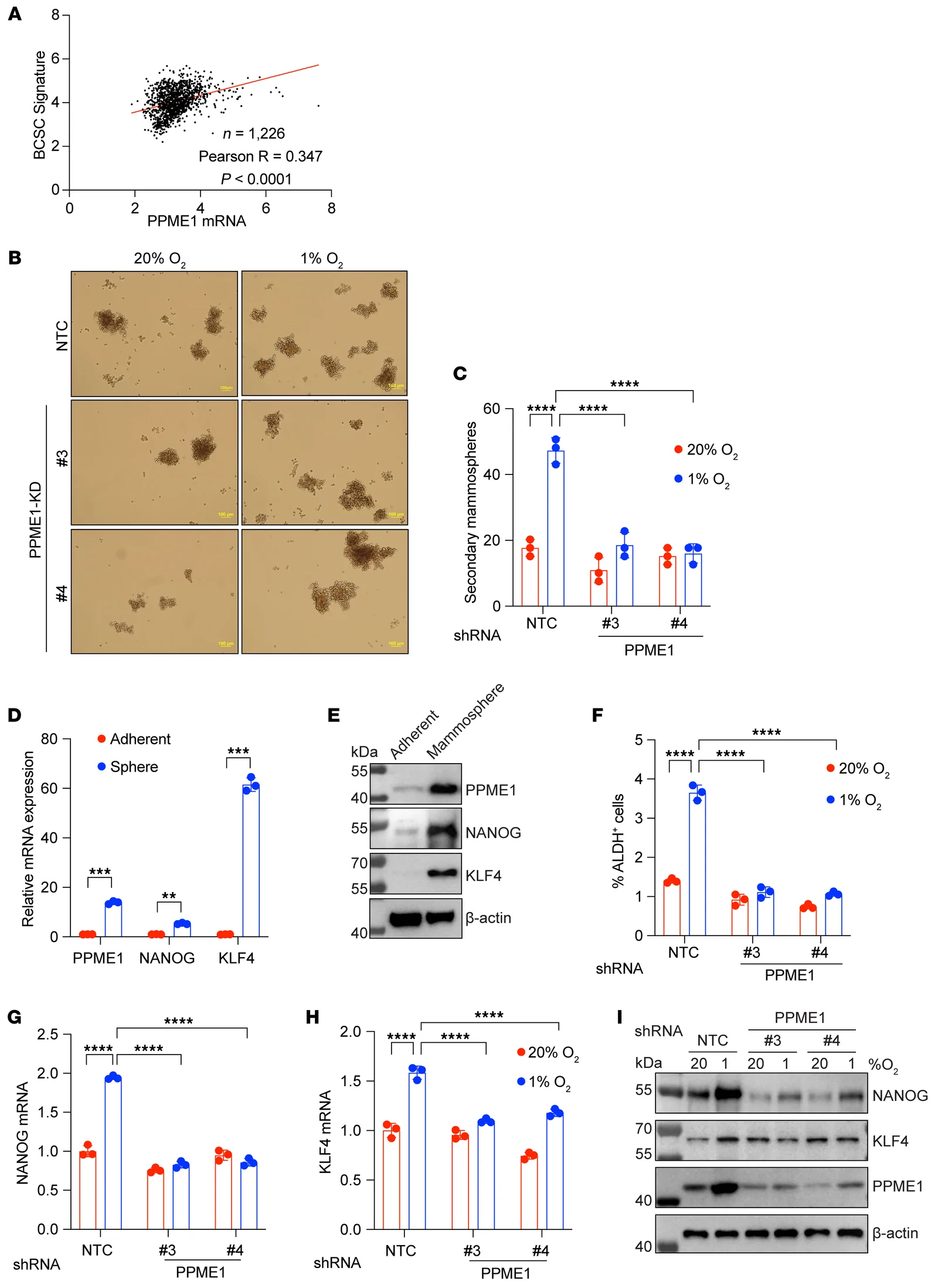
PPME1 is required for hypoxia-induced BCSC specification. (**A**) Expression of PPME1 mRNA and a BCSC signature in primary BC samples were analyzed by Pearson’s test. (**B** and **C**) Representative light microscope images (**B**; scale bar: 100 μm) and quantification (**C**) of secondary mammospheres generated from MDA-MB-231 subclones expressing NTC or PPME1 shRNA (#3 or #4) and exposed to 20% or 1% O_2_ for 72 hours. ****P < 0.0001, 2-way ANOVA with Tukey’s multiple comparisons test. (**D** and **E**) mRNA and protein were extracted from MDA-MB-231 adherent cells and secondary mammospheres, followed by RT-qPCR (**D**) and immunoblot assays (**E**), respectively. RT-qPCR results were normalized to adherent cells. **P < 0.01, ***P < 0.001, 2-tailed Student’s *t* test. (**F**) MDA-MB-231 subclones were exposed to 20% or 1% O_2_ for 72 hours, and the percentage of cells with high aldehyde dehydrogenase-expressing (ALDH^+^) activity was determined by ALDEFLUOR assay. ****P < 0.0001, 2-way ANOVA with Tukey’s multiple comparisons test. (**G**–**I**) MDA-MB-231 subclones were exposed to 20% or 1% O_2_ for 24 or 48 hours, followed by RT-qPCR (**G** and **H**) and immunoblot assays (**I**), respectively. ****P < 0.0001, 2-way ANOVA with Tukey’s multiple comparisons test. In all bar graphs, data are presented as mean ± SD (*n* = 3).

**Figure 4:**
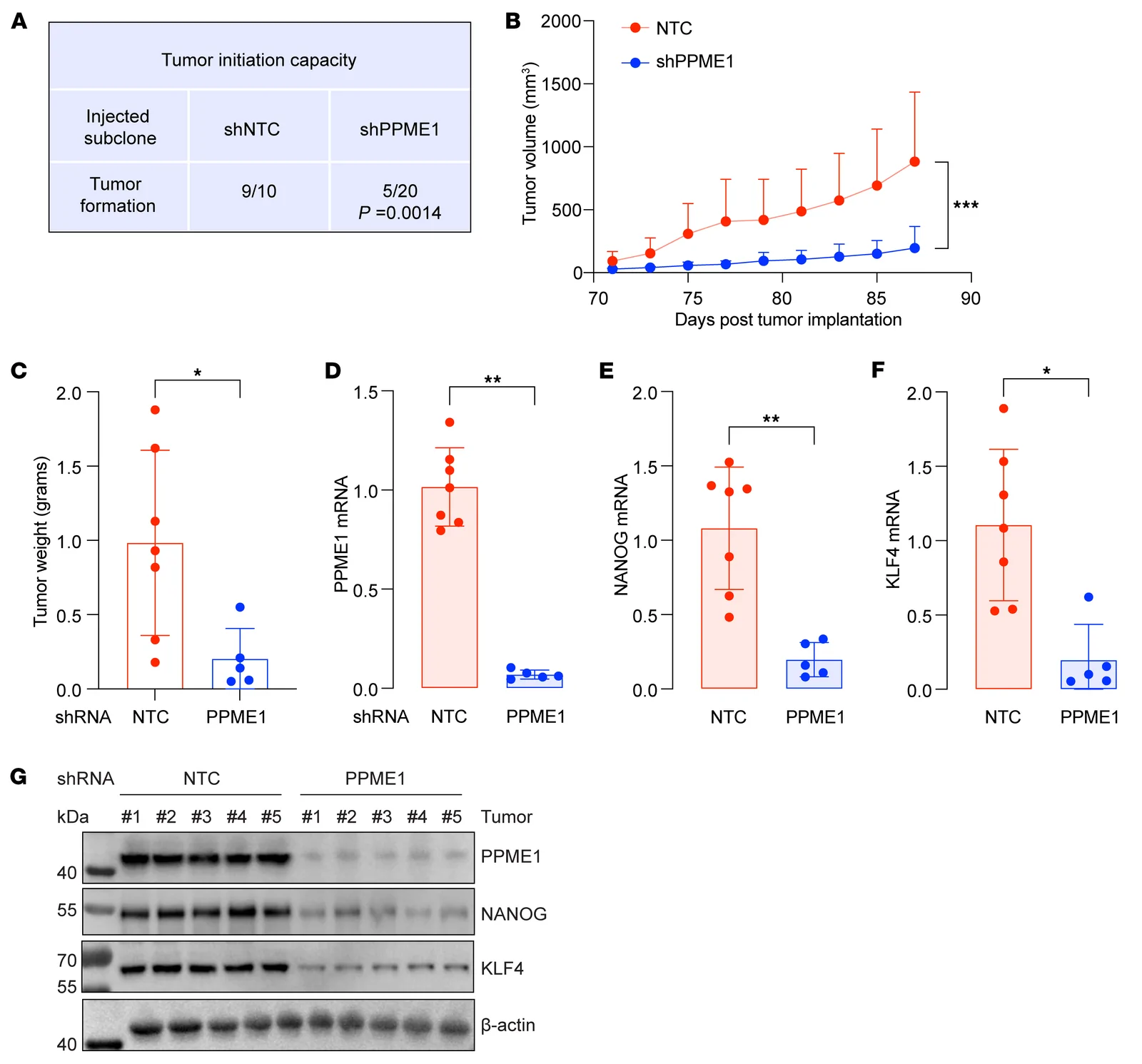
PPME1 KD impairs tumor initiation and growth. (**A**) MDA-MB-231 subclones expressing NTC or PPME1 shRNA (#3 or #4) were implanted into the mammary fat pad (MFP) of 7-week-old female SCID mice, and the mice were scored for the presence of a palpable tumor on day 87 after implantation. The two PPME1-KD groups were pooled for comparison with the NTC group using Fisher’s exact test. (**B**) Primary tumor volume was measured every 2 days. Mean ± SD are shown; ***P < 0.001, 2-way ANOVA with Šidák’s multiple comparisons test. (**C**) Tumors were harvested and weighed. Mean ± SD (*n* = 5–7; 2 mice in the NTC group died during the tumor measurement period). *P < 0.05, 2-tailed Student’s *t* test. (**D**–**G**) mRNA and protein were extracted from tumor tissues, and aliquots were subjected to RT-qPCR (**D**–**F**) and immunoblot assays (**G**), respectively. Mean ± SD (*n* = 5–7) are shown. *P < 0.05, **P < 0.01, Mann-Whitney test.

**Figure 5:**
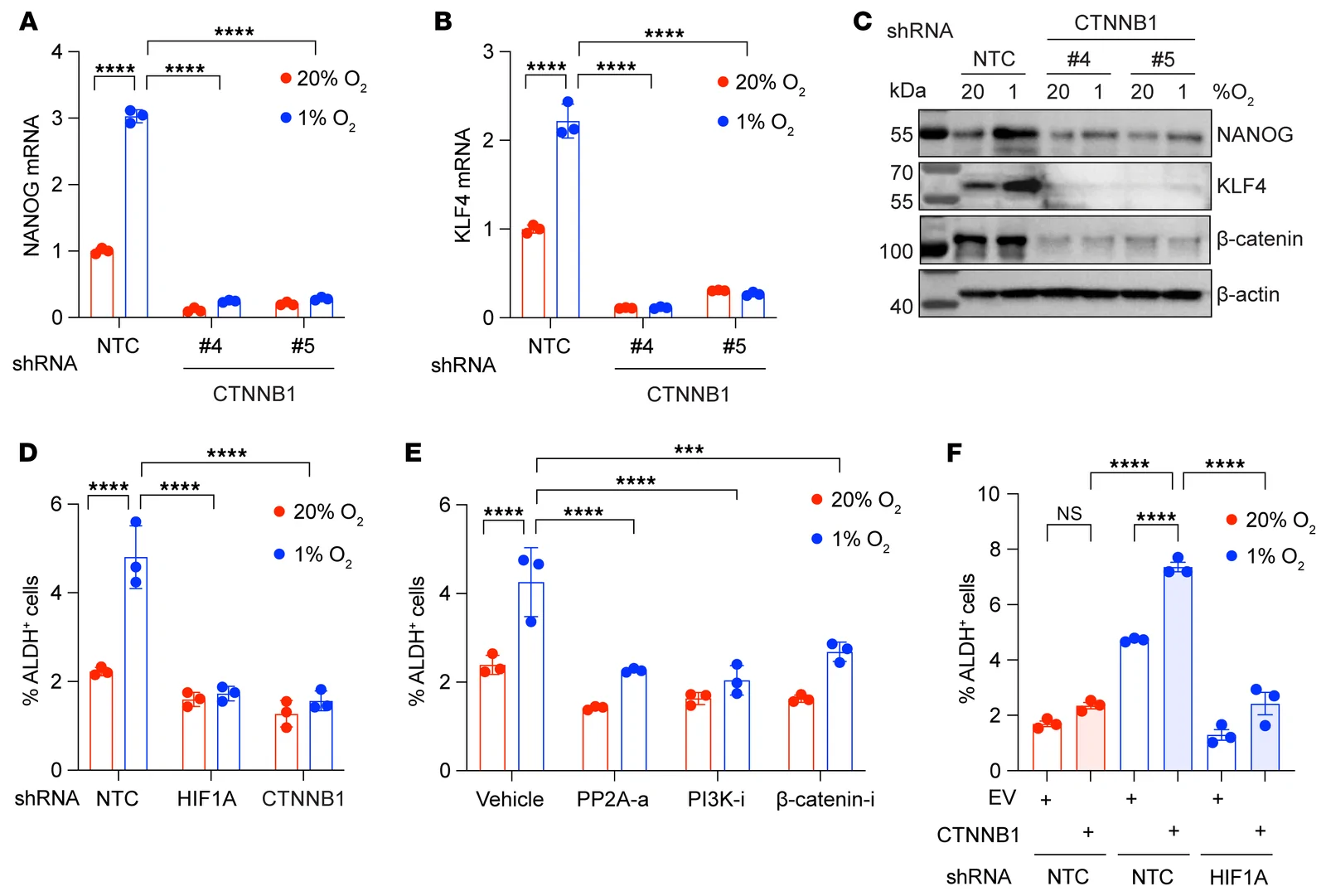
β-Catenin promotes hypoxia-induced and HIF-1–dependent BCSC enrichment. (**A** and **B**) MDA-MB-231 subclones expressing NTC or β-catenin shRNA (#4 or #5) were exposed to 20% or 1% O_2_ for 24 hours, followed by RT-qPCR with primers specific for NANOG (**A**) or KLF4 (**B**) mRNA. ****P < 0.0001, 2-way ANOVA with Tukey’s multiple comparisons test. (**C**) MDA-MB-231 subclones were exposed to 20% or 1% O_2_ for 48 hours, followed by immunoblot assays. (**D**) MDA-MB-231 subclones expressing NTC, HIF-1α, or β-catenin shRNA (#4) were exposed to 20% or 1% O_2_ for 72 hours, and the percentage of ALDH^+^ cells was determined. ****P < 0.0001, 2-way ANOVA with Tukey’s multiple comparisons test. (**E**) MDA-MB-231 cells were treated with DMSO, PP2A activator SMAP-2 (10 μM), PI3K inhibitor LY294002 (5 μM), or β-catenin inhibitor MSAB (5 μM) and exposed to 20% or 1% O_2_ for 72 hours, and the percentage of ALDH^+^ cells was determined. ***P < 0.001, ****P < 0.0001, 2-way ANOVA with Tukey’s multiple comparisons test. (**F**) MDA-MB-231 NTC and HIF1A-KD subclones were transfected with empty vector (EV) or β-catenin expression vector (CTNNB1). Cells were exposed to 20% or 1% O_2_ for 72 hours, and the percentage of ALDH^+^ cells was determined. ****P < 0.0001, 2-way ANOVA with Tukey’s multiple comparisons test. In all bar graphs, data are presented as mean ± SD (*n* = 3).

**Figure 6:**
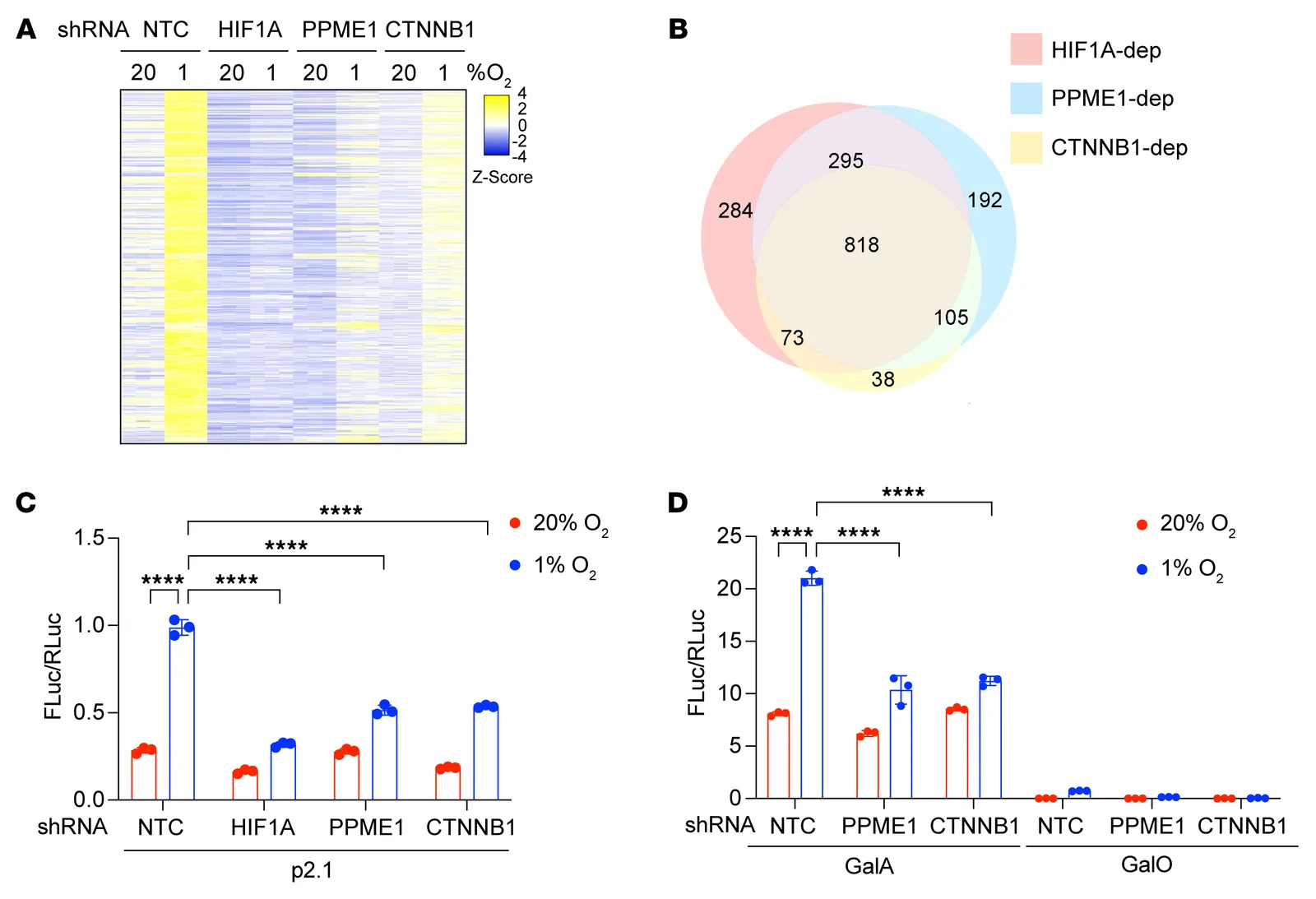
HIF-1, PPME1, and β-catenin are required for a broad transcriptional response to hypoxia. (**A**) SUM159 shRNA-expressing subclones were exposed to 20% or 1% O_2_ for 24 hours, and RNA was extracted for sequencing (*n* = 3 independent samples each). The expression of HIF-1α–dependent hypoxia-induced RNAs is shown for each of the 4 shRNA subclones as a heatmap. (**B**) Venn analysis shows the overlap among HIF-1α–dependent, PPME1-dependent and β-catenin–dependent hypoxia-induced RNAs identified by RNA-seq. (**C**) MDA-MB-231 subclones were transiently cotransfected with HIF-1 reporter plasmid p2.1, which expresses firefly luciferase (FLuc) under the control of an hypoxia response element upstream of an SV40 promoter, and control reporter pSV-RL, which expresses Renilla luciferase (RLuc) under the control of the SV40 promoter alone. At 24 hours after transfection, the cells were exposed to 20% or 1% O_2_ for 24 hours, and FLuc/RLuc ratios were determined. ****P < 0.0001, 2-way ANOVA with Tukey’s multiple comparisons test. (**D**) MDA-MB-231 subclones were transiently cotransfected with plasmid pGalO or pGalA, which encode the Gal4 DNA-binding domain either alone (GalO) or fused to HIF-1α residues 531–826 (GalA); reporter pG5E1bLuc containing 5 GAL4-binding sites and a basal *E1b* promoter upstream of FLuc coding sequences; and RLuc reporter pSV-RL. At 24 hours after transfection, the cells were exposed to 20% or 1% O_2_ for 24 hours, and FLuc/RLuc ratios were determined. ****P < 0.0001, 2-way ANOVA with Tukey’s multiple comparisons test. In both bar graphs, data are presented as mean ± SD (*n* = 3).

**Figure 7:**
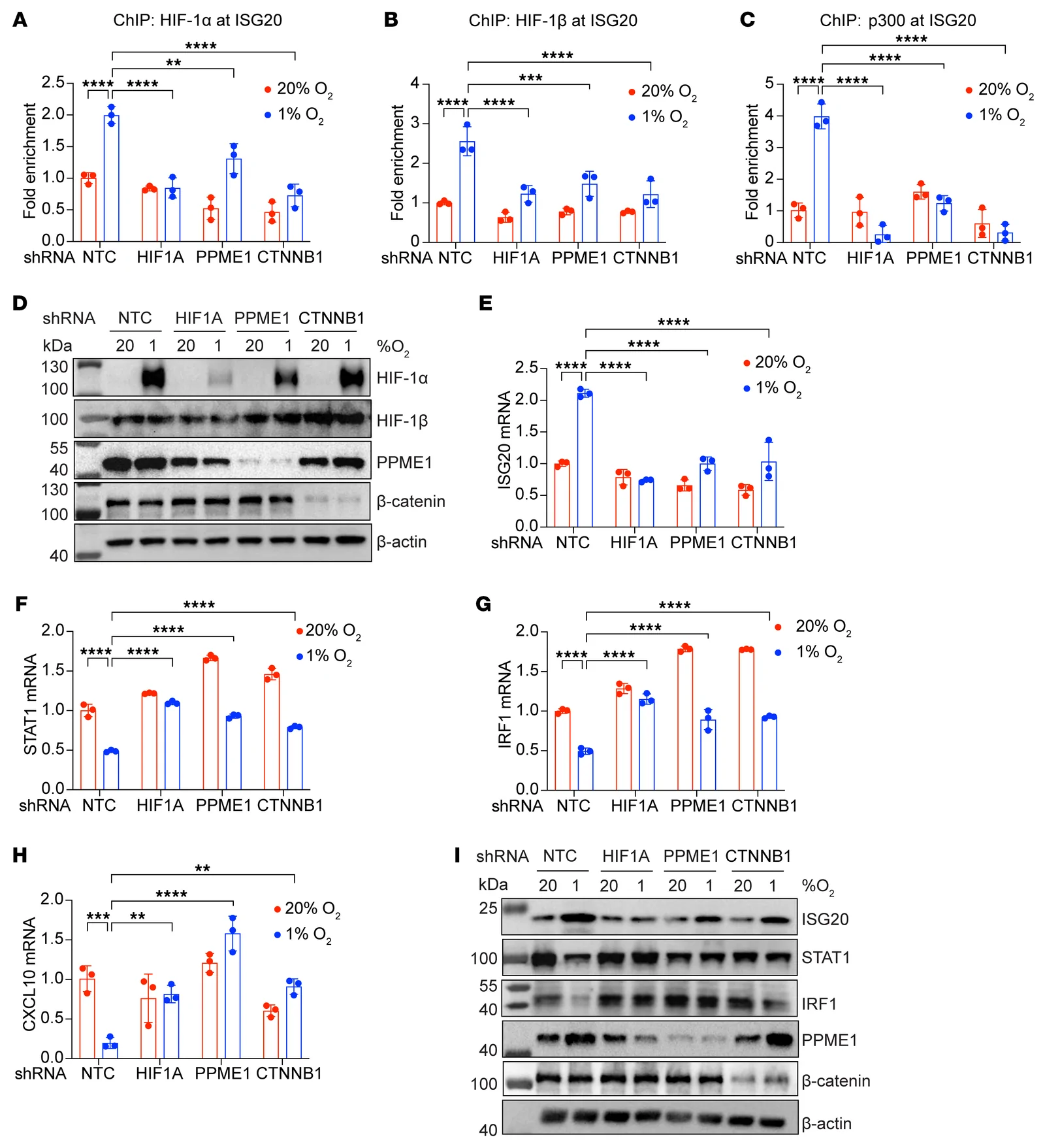
PPME1 and β-catenin are required for hypoxia-mediated repression of the STAT1/IRF1/CXCL10 axis via transcriptional regulation of *ISG20*. (**A**–**C**) MDA-MB-231 subclones expressing NTC, HIF-1α, PPME1, or β-catenin shRNA were exposed to 20% or 1% O_2_ for 16 hours, followed by ChIP-qPCR assays using antibodies against HIF-1α (**A**), HIF-1β (**B**) or p300 (**C**) and primers flanking the HIF-1 binding site of *ISG20*. **P < 0.01, ***P < 0.001, ****P < 0.0001, 2-way ANOVA with Tukey’s multiple comparisons test. (**D**) MDA-MB-231 subclones were exposed to 20% or 1% O_2_ for 16 hours, and immunoblot assays were performed. (**E**–**I**) MDA-MB-231 subclones were exposed to 20% or 1% O_2_ for 48 hours, followed by RT-qPCR (**E**–**H**) or immunoblot assays (**I**). **P < 0.01, ***P < 0.001, ****P < 0.0001, 2-way ANOVA with Tukey’s multiple comparisons test. In all bar graphs, data are presented as mean ± SD (*n* = 3).

**Figure 8:**
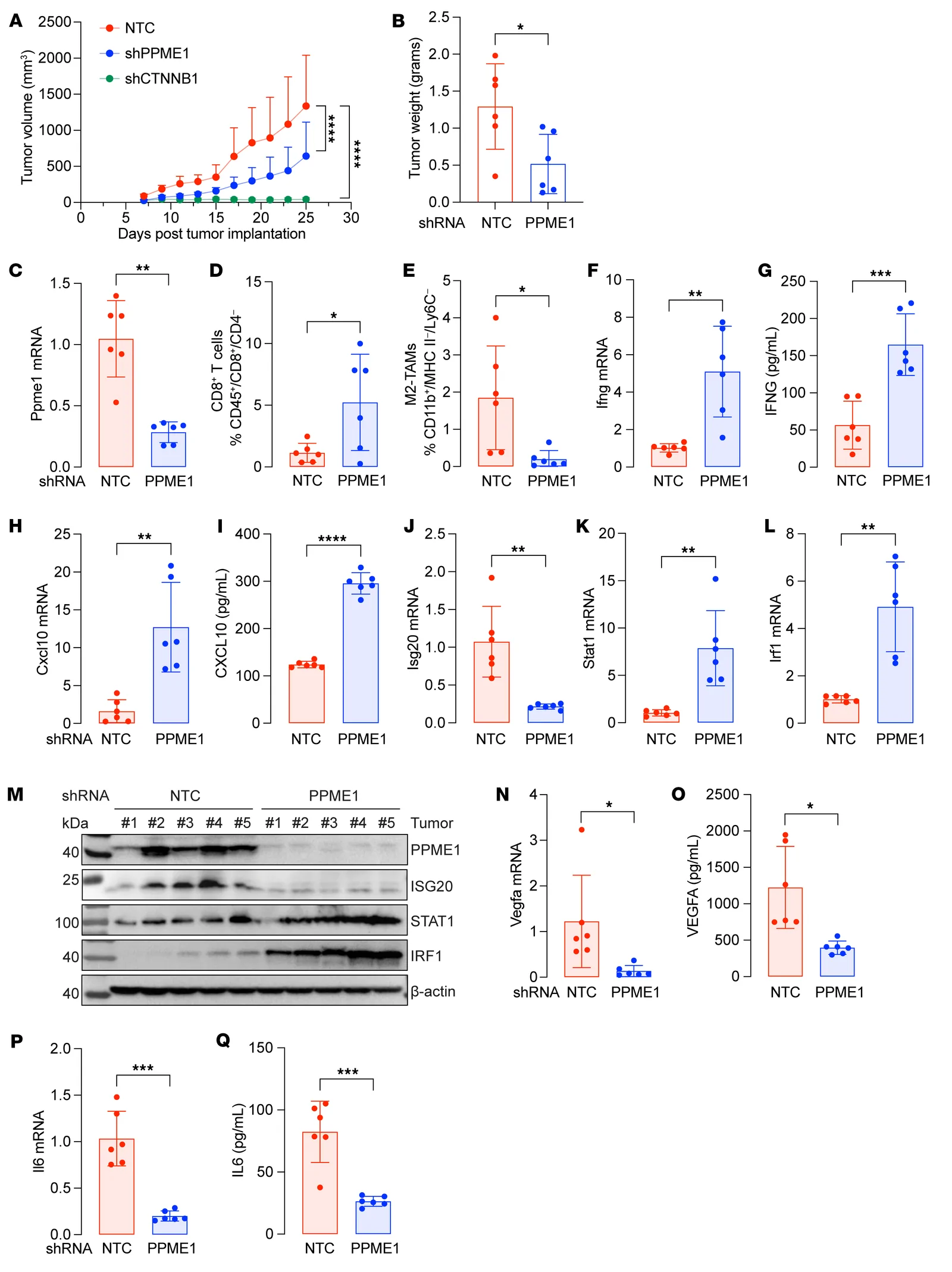
PPME1 KD suppresses BC growth in an immune-mediated manner through inhibition of ISG20 and VEGFA expression. (**A** and **B**) EMT6 subclones expressing NTC, PPME1, or β-catenin shRNA were implanted in the mammary fat pad of female BALB/c mice, and tumor growth was monitored (**A**). ****P < 0.0001, 2-way ANOVA with Tukey’s multiple comparisons test. On day 25, NTC and PPME1-KD tumors were harvested and weighed (**B**). *P < 0.05, Welch’s *t* test. (**C**) PPME1 mRNA expression in tumor tissues was analyzed by RT-qPCR and normalized to the result from NTC group. **P < 0.01, Welch’s *t* test. (**D**) The percentage of CD45^+^/CD8^+^/CD4^–^ T cells among live cells in the tumors was determined by flow cytometry. *P < 0.05, Welch’s *t* test. (**E**) The percentage of CD11b^+^/MHC II^–^/Ly6C^–^ M2-TAMs among live cells was determined by flow cytometry. *P < 0.05, Welch’s *t* test. (**F** and **G**) Analysis of IFN-γ (Ifng) mRNA expression by RT-qPCR (**F**) and protein levels by ELISA (**G**) in tumors was performed. **P < 0.01, ***P < 0.001, Welch’s *t* test. (**H** and **I**) Analysis of CXCL10 mRNA expression by RT-qPCR (**H**) and protein levels by ELISA (**I**) in tumors was performed. **P < 0.01, ****P < 0.0001, Welch’s *t* test. (**J**–**M**) mRNA and protein were extracted from tumors and RT-qPCR (**J**–**L**) and immunoblot assays (**M**) were performed. **P < 0.01, Welch’s *t* test. (**N** and **O**) VEGFA mRNA (**N**) and protein (**O**) levels were analyzed in tumors. *P < 0.05, Welch’s *t* test. (**P** and **Q**) IL6 mRNA (**P**) and protein (**Q**) levels were analyzed in tumors. ***P < 0.001, Welch’s *t* test. In all bar graphs, data are presented as mean ± SD (*n* = 6 tumors).

**Figure 9:**
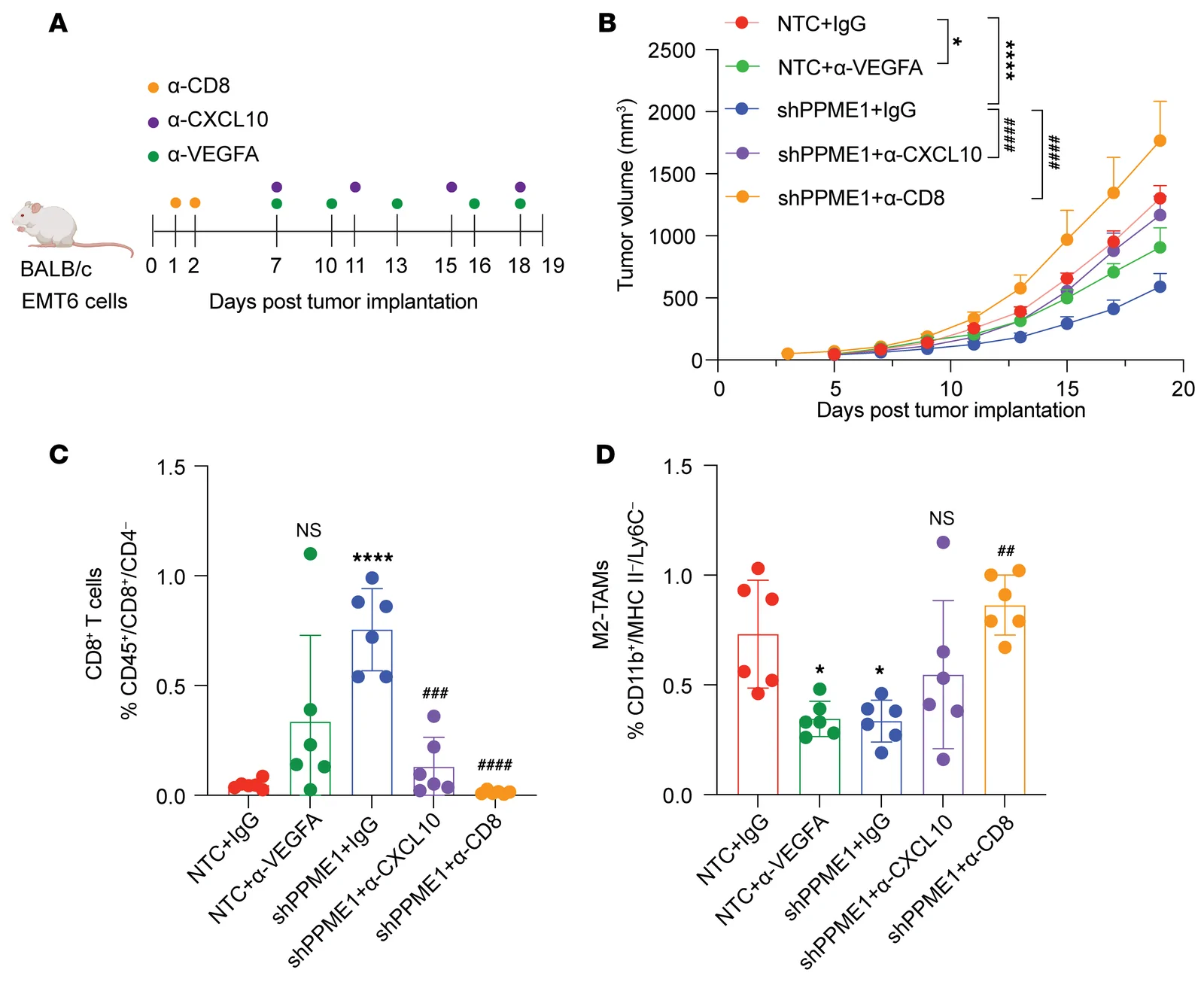
PPME1-KD suppresses tumor growth through CXCL10-driven CD8^+^ T cell recruitment, while VEGFA sustains an immunosuppressive microenvironment through M2-TAM enrichment. (**A**) BALB/c mice were orthotopically implanted with EMT6 subclones expressing NTC or PPME1 shRNA. Mice bearing NTC tumors were treated by intraperitoneal injection with anti-VEGFA antibody or IgG2a isotype control antibody at 100 μg per dose on days 7, 10, 13, 16, and 18 after tumor implantation. Mice bearing shPPME1 tumors were treated with anti-CD8α antibody at 100 μg per dose on days 1 and 2 or with anti-CXCL10 antibody or IgG isotype control antibody at 200 μg on day 7 and 100 μg on days 11, 15, and 18. (**B**) Tumor growth curves are shown as mean ± SD (*n* = 6). *P < 0.05 and ****P < 0.0001 vs. NTC + IgG; ^####^*P* < 0.0001 vs. shPPME1 + IgG, 2-way ANOVA with Tukey’s multiple comparisons test. (**C**) The percentage of CD45^+^/CD8^+^/CD4^–^ T cells among live cells was determined by flow cytometry. ****P < 0.0001 vs. NTC + IgG; ^###^*P* < 0.001 and ^####^*P* < 0.0001 vs. shPPME1 + IgG, 1-way ANOVA with Tukey’s multiple comparisons test. (**D**) The percentage of CD11b^+^/MHC II^–^/Ly6C^–^ M2-TAMs among live cells was determined by flow cytometry. *P < 0.05 vs. NTC + IgG; ^##^*P* < 0.01 vs. shPPME1 + IgG, 1-way ANOVA with Tukey’s multiple comparisons test.

**Figure 10:**
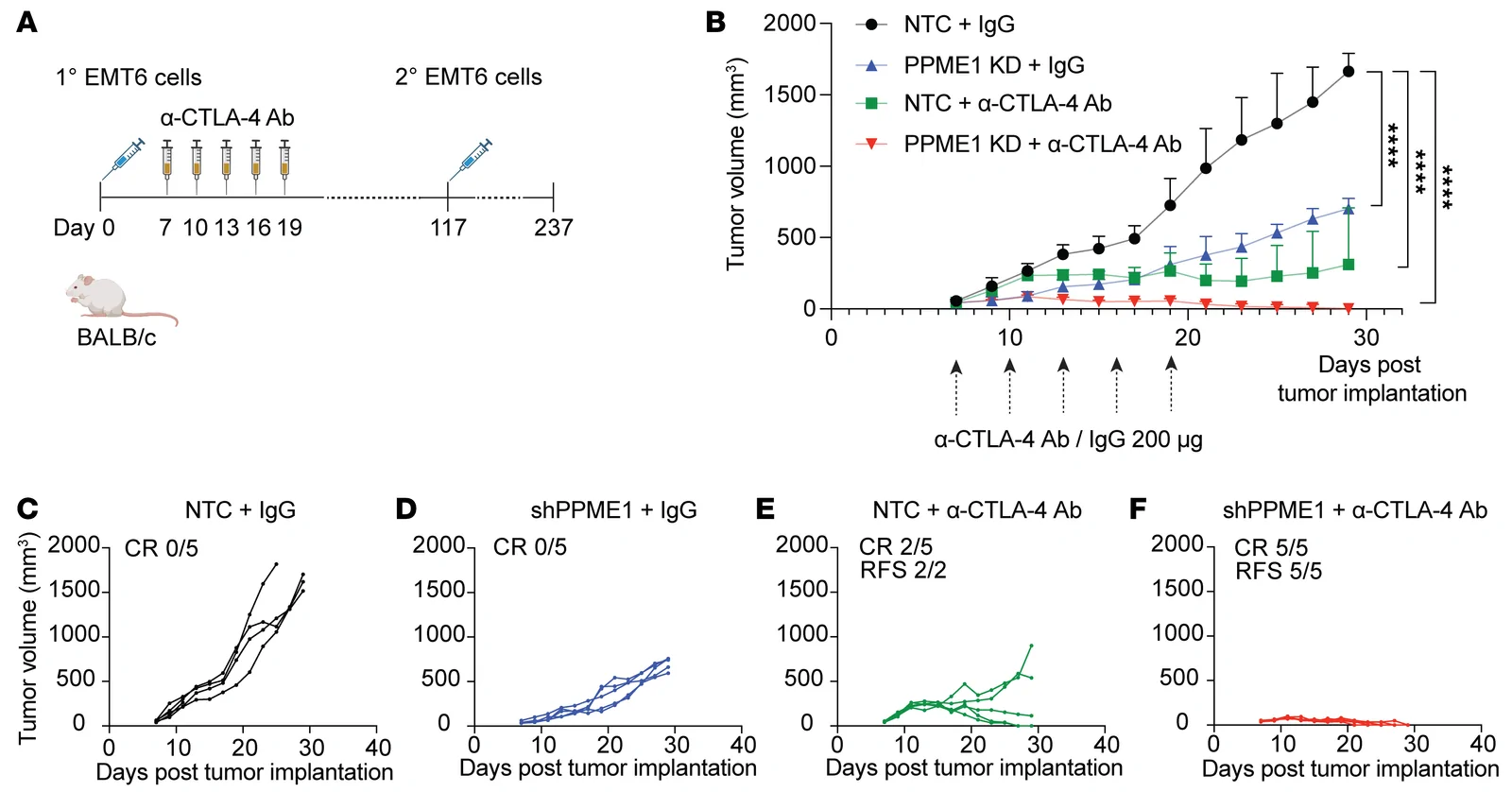
PPME1-KD enhances the efficacy of anti-CTLA-4 immunotherapy. (**A**) BALB/c mice were orthotopically implanted with EMT6 subclones expressing NTC or PPME1 shRNA. When tumors became measurable, mice were treated by intraperitoneal injection with anti-CTLA-4 antibody or IgG isotype control. (**B**) Tumor growth curves are shown as mean ± SD (*n* = 5, 1 mouse in the NTC + IgG group died during the tumor measurement period); ****P < 0.0001, 2-way ANOVA with Tukey’s multiple comparisons test. (**C**–**F**) Individual tumor growth curves are shown (1 mouse in the NTC + IgG group was euthanized on day 25 due to tumor burden). The number of mice in each group that achieved a complete response (CR) and recurrence-free survival (RFS) on day 237 after secondary tumor cell injection on day 117 is indicated.

## Version 1 10/01/2026

Electronic publication
