# Supplementary material for: HIF-1–mediated PPME1 expression promotes breast cancer progression via AKT activation and β-catenin recruitment
Source: J Clin Invest. 2026 Oct 1;136(19):e205415. doi: 10.1172/JCI205415 (PMC13626848; doi:10.1172/JCI205415)
Supplement: Supplementary file 1 [file jci-136-205415-s084.pdf]

Supplemental Material

HIF-1–mediated PPME1 expression promotes breast cancer progression via AKT activation and β-catenin recruitment

Yajing Lyu[1,2], Varen Talwar[4], Yongkang Yang[1,3], Si-Sim Kang[4], Shuyi Li[1], Shaima Salman[1,3], Daiana Drehmer[1,2], Yufeng Wang[1,2], Chelsey Chen[1], Vijay Ramu[4], Sujin Kim[4], Dylan Park[4], Tina Yi-Ting Huang[1,2], Emmanuel Datan[1,2], Dominic Dordai[1,2], Jonathan P. Schneck[1,3], and Gregg L. Semenza[1,2,3]

[1]Institute for Cell Engineering, Johns Hopkins University School of Medicine, Baltimore, MD 21205, USA. [2]Department of Genetic Medicine, Johns Hopkins University School of Medicine, Baltimore, MD 21205, USA. [3]Sidney Kimmel Comprehensive Cancer Center at Johns Hopkins, Baltimore, MD 21231, USA. [4]Johns Hopkins University, Baltimore, MD 21218, USA.

Corresponding Author: Gregg L. Semenza, Miller Research Building, Suite 671, 733 N. Broadway, Baltimore, MD 21205. Fax: 443-287-5618; E-mail: gsemenza@jhmi.edu

Supplemental Tables 1-10

Supplemental Figures 1-10

Supplemental Methods

References

Supplemental Tables

Supplemental Table 1. Cell lines and culture media.

Cell line	Source	Medium type	Medium manufacturer	Medium catalog #
MDA-MB-231	ATCC	DMEM	Corning	10-013-CV
MCF-7	ATCC	DMEM	Corning	10-013-CV
SUM149	BIOIVT	Ham's F-12	Corning	10-080-CV
SUM159	BIOIVT	DMEM/F12	Corning	10-092-CV
T-47D	ATCC	RPMI-1640	Corning	10-040-CV
HCC1954	ATCC	RPMI-1640	Corning	10-040-CV
BT-474	ATCC	RPMI-1640	Corning	10-040-CV
4T1	ATCC	DMEM	Corning	10-013-CV
4T1-BR5	Suyun Huang, VCU	DMEM	Corning	10-013-CV
EMT6	ATCC	Waymouth's	Invitrogen	11220-035
EO771	ATCC	DMEM	Corning	10-013-CV
HEK293T	ATCC	DMEM	Corning	10-013-CV

Supplemental Table 2. Nucleotide sequence of primers used for RT-qPCR.

Gene	Nucleotide sequence (5' to 3')
Human *PPME1*	Forward: CCCTGGAAGAAAGCGGGAC
	Reverse: TGAACCACTCTTGTAGACTCGAA
Human *CTNNB1*	Forward: GGAGACGGAGGAAGGTCTGA
	Reverse: GAGTAGCCATTGTCCACGCT
Human *NANOG*	Forward: TTTGTGGGCCTGAAGAAAACT
	Reverse: AGGGCTGTCCTGAATAAGCAG
Human *KLF4*	Forward: CGGACATCAACGACGTGAG
	Reverse: GACGCCTTCAGCACGAACT
Human *ISG20*	Forward: CTCGTTGCAGCCTCGTGAA
	Reverse: CGGGTTCTGTAATCGGTGATCTC
Human *STAT1*	Forward: ATCAGGCTCAGTCGGGGAATA
	Reverse: TGGTCTCGTGTTCTCTGTTCT
Human *IRF1*	Forward: CTGTGCGAGTGTACCGGATG
	Reverse: ATCCCCACATGACTTCCTCTT
Human *CXCL10*	Forward: GTGGCATTCAAGGAGTACCTC
	Reverse: TGATGGCCTTCGATTCTGGATT
Human *VEGFA*	Forward: AGGGCAGAATCATCACGAAGT
	Reverse: AGGGTCTCGATTGGATGGCA
Human *RPL13A*	Forward: CTCAAGGTCGTGCGTCTG
	Reverse: TGGCTTTCTCTTTCCTCTTCTC

Human *18S rRNA*	Forward: CGGCGACGACCCATTCGAAC
	Reverse: GAATCGAACCCTGATTCCCCGTC
Mouse *Ppme1*	Forward: TCGAAACCTTGAGTCTGCCC
	Reverse: GCCAGCTCAATTCTCCAGGT
Mouse *Ctnnb1*	Forward: GGTGCTGACTTTGCTTGCTT
	Reverse: CCGCACGTATCCCCGATAAA
Mouse *Isg20*	Forward: TGGGCCTCAAAGGGTGAGT
	Reverse: CGGGTCGGATGTACTTGTCATA
Mouse *Stat1*	Forward: TCACAGTGGTTCGAGCTTCAG
	Reverse: CGAGACATCATAGGCAGCGTG
Mouse *Irf1*	Forward: ATGCCAATCACTCGAATGCG
	Reverse: TTGTATCGGCCTGTGTGAATG
Mouse *Cxcl10*	Forward: CCAAGTGCTGCCGTCATTTTC
	Reverse: TCCCTATGGCCCTCATTCTCA
Mouse *Ifng*	Forward: TGAACGCTACACACTGCATCTTGG
	Reverse: CGACTCCTTTTCCGCTTCCTGAG
Mouse *Il6*	Forward: TAGTCCTTCCTACCCCAATTTCC
	Reverse: TTGGTCCTTAGCCACTCCTTC
Mouse *Vegfa*	Forward: CTGCCGTCCGATTGAGACC
	Reverse: CCCCTCCTTGTACCACTGTC
Mouse *18S rRNA*	Forward: GTAACCCGTTGAACCCCATT
	Reverse: CCATCCAATCGGTAGTAGCG

Supplemental Table 3. Antibodies for immunoblot assays.

Primary Ab	Source	Catalog #	Usage
HIF-1α	BD Biosciences	610959	1:1000
HIF-1α	Cayman Chemical	10006421	1:1000
HIF-2α	Novus Biologicals	NB100-122	1:1000
PPME1	Novus Biologicals	NBP2-19903	1:1000
PP2Ac	Novus Biologicals	NB100-2260	1:1000
PP2Ac methyl-Leu309	Sigma-Aldrich	MABE1809	1:1000
AKT1	Novus Biologicals	NBP2-01724	1:1000
AKT1 phospho-Ser473	Novus Biologicals	NBP1-69923	1:1000
AKT1 phospho-Thr308	Novus Biologicals	MAB7419	1:1000
β-catenin	Novus Biologicals	NBP1-32239	1:1000
β-catenin phospho-Ser552	Novus Biologicals	NBP3-05441	1:1000
ISG20	Novus Biologicals	NBP3-35464	1:500
NANOG	Novus Biologicals	NB100-58842	1:500
KLF4	R&D Systems	AF3640	1:1000
KLF4	R&D Systems	AF3158	1:1000
STAT1	Novus Biologicals	PAF-ST1	1:1000
IRF1	Novus Biologicals	NBP2-76419	1:1000
β-actin	Santa Cruz Biotechnology	sc-47778	1:5000
**Secondary Ab**	**Source**	**Identifier**	**Usage**
Mouse IgG HRP	Cytiva	NA931	1:2000

Rabbit IgG HRP	Cytiva	NA934	1:2000
Goat IgG HRP	Santa Cruz Biotechnology	sc-2354	1:2000

Supplemental Table 4. Antibodies for ChIP.

Primary Ab	Source	Catalog #	Usage
HIF-1α	Novus Biologicals	NB100-479	2 µg
HIF-1β	Novus Biologicals	NB100-110	2 µg
HIF-2α	Novus Biologicals	NB100-122	2 µg
p300	Novus Biologicals	NB500-161	4 µg
RNA Polymerase II phospho-Ser2	Novus Biologicals	NBP2-59215	2 µg
RNA Polymerase II phospho-Ser5	Novus Biologicals	NBP2-59219	2 µg
RNA Polymerase II	Novus Biologicals	NBP2-32080	2 µg

Supplemental Table 5. Nucleotide sequence of primers used for ChIP-qPCR.

**Transcription factor binding site**	**Nucleotide sequence (5' to 3')**
Human *PPME1* HRE	Forward: GCTTCGCCTACTGTTTGACT
	Reverse: GGAGAAGGGGTATGGGTCAC
Human *ISG20* HRE	Forward: GTAGCCCAGGAGATGGAGCA
	Reverse: CAGCTCACGTCTGCCTCTCT
Human *RPL13A* Non-HRE	Forward: GAGGCGAGGGTGATAGAG
	Reverse: ACACACAAGGGTCCAATTC

Supplemental Table 6. shRNAs in lentiviral vectors and other plasmids.

shRNA	Source	Identifier
Human HIF1A shRNA	Sigma Aldrich	TRCN0000003810
Human HIF2A shRNA	Sigma Aldrich	TRCN0000003806
Human PPME1 shRNA-#1	Sigma Aldrich	TRCN0000003057
Human PPME1 shRNA-#2	Sigma Aldrich	TRCN0000003059
Human PPME1 shRNA-#3	Sigma Aldrich	TRCN0000003060
Human PPME1 shRNA-#4	Sigma Aldrich	TRCN0000010755
Human PPME1 shRNA-#5	Sigma Aldrich	TRCN0000320885
Mouse PPME1 shRNA-#1	Sigma Aldrich	TRCN0000032959
Mouse PPME1 shRNA-#2	Sigma Aldrich	TRCN0000032960
Mouse PPME1 shRNA-#3	Sigma Aldrich	TRCN0000032961
Mouse PPME1 shRNA-#4	Sigma Aldrich	TRCN0000032962
Mouse PPME1 shRNA-#5	Sigma Aldrich	TRCN0000032963
Human CTNNB1 shRNA-#1	Sigma Aldrich	TRCN0000314991
Human CTNNB1 shRNA-#2	Sigma Aldrich	TRCN0000314990
Human CTNNB1 shRNA-#3	Sigma Aldrich	TRCN0000314921
Human CTNNB1 shRNA-#4	Sigma Aldrich	TRCN0000350477
Human CTNNB1 shRNA-#5	Sigma Aldrich	TRCN0000003846
Mouse CTNNB1 shRNA-#1	Sigma Aldrich	TRCN0000012689
Mouse CTNNB1 shRNA-#2	Sigma Aldrich	TRCN0000012690
Mouse CTNNB1 shRNA-#3	Sigma Aldrich	TRCN0000012691

Mouse CTNNB1 shRNA-#4	Sigma Aldrich	TRCN0000012692
Mouse CTNNB1 shRNA-#5	Sigma Aldrich	TRCN0000314920
pcDNA3-HIF-1α	Pai-Sheng Chen, National Cheng Kung University	https://www.jci.org/articles/view/89212
pcDNA3-β-catenin	Addgene	Plasmid #16828

Supplemental Table 7. Antibodies used for flow cytometry (FC) and ELISA kits.

Primary Ab	Source	Catalog #	Application	Usage
CD45	Biolegend	147708	FC	1.5 µl
CD3	Biolegend	100206	FC	1.5 µl
CD8	Biolegend	100743	FC	1.5 µl
CD4	Biolegend	100412	FC	1.5 µl
CD11b	Biolegend	101212	FC	1.5 µl
CD11c	Biolegend	117328	FC	1.5 µl
Ly6G	Biolegend	127608	FC	1.5 µl
Ly6C	Biolegend	128031	FC	1.5 µl
MHC-II	Biolegend	107606	FC	1.5 µl
LIVE/DEAD™ Fixable Aqua Dead Cell Stain Kit	ThermoFisher	L34965	FC	0.1 µl
IFNG	R&D Systems	MIF00	ELISA	-
CXCL10	R&D Systems	DY466	ELISA	-
IL6	R&D Systems	DY406-05	ELISA	-
VEGFA	Novus Biologicals	MMV00-1	ELISA	-

Supplemental Table 8. Chemical inhibitors.

Inhibitor	Source	Catalog #
LB-100	Selleck Chemicals	S7537
SMAP-2	MedChemExpress	HY-120272
LY294002	Selleck Chemicals	S1105
MSAB	MedChemExpress	HY-120697

Supplemental Table 9. Additional reagents and kits.

**Item**	**Source**	**Catalog #**
TRIzol	Invitrogen	15596018
High-Capacity RNA-to-cDNA kit	Thermo Fisher Scientific	4387406
SYBR Green	Bio-Rad	1725125
ECL prime western blotting detection reagent	Cytiva	RPN2236
PolyJet	SignaGen Laboratories	SL100688
Puromycin	Thermo Fisher Scientific	A1113803
Ser/Thr phosphatase assay kit	Upstate Biotechnology	17-127
6-well ultra-low attachment plate	Corning	3471
MammoCult™ medium	STEMCELL Technologies	05620
ALDEFLUOR assay	STEMCELL Technologies	01700
DNase I	Invitrogen	AM1906
Type I collagenase	Sigma-Aldrich	C0130
Bovine serum albumin (BSA)	Sigma-Aldrich	A9647
Fetal bovine serum (FBS)	GeminiBio	100-106
L-glutamine	Thermo Fisher Scientific	25030081

Supplemental Table 10. Antibodies used in animal experiments.

Antibody	Source	Catalog #
Anti-mouse CTLA-4 (CD152)	Bio X Cell	BP0131
Polyclonal Syrian hamster IgG	Bio X Cell	BP0087
Anti-mouse VEGF-A (Clone 2G11-2A05)	Bio X Cell	BE0399
Rat IgG2a isotype control, anti-trinitrophenol	Bio X Cell	BE0089
Anti-mouse CXCL10 (IP-10) (Clone 1F11)	Bio X Cell	BE0440
Anti-mouse CD8α (Clone 2.43)	Bio X Cell	BE0061
Polyclonal Armenian hamster IgG	Bio X Cell	BE0091

Supplemental Figures

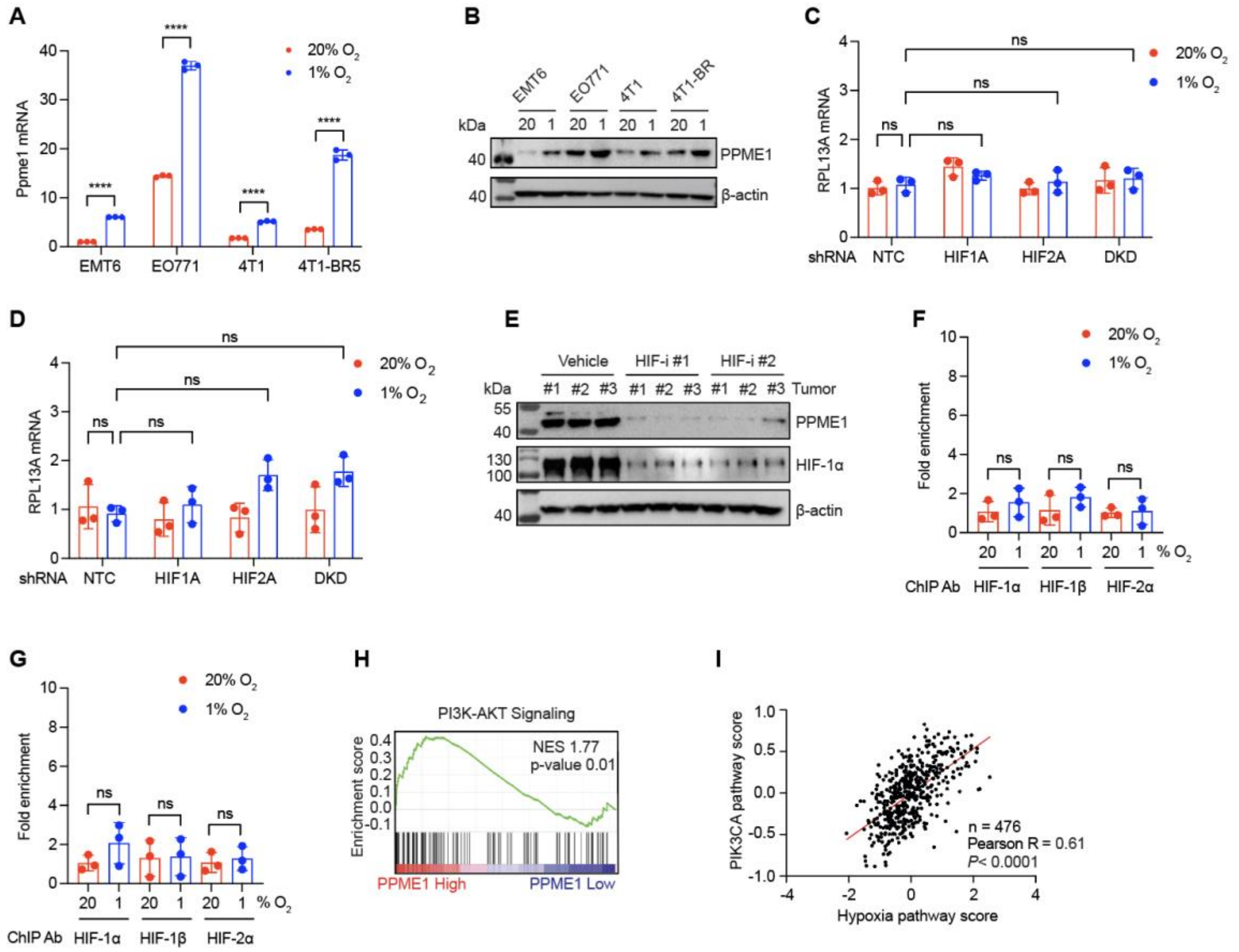

Supplemental Figure 1. PPME1 expression is mediated by HIF-1 in hypoxic BC cells. (**A**) Mouse BC cell lines were exposed to 20% or 1% O_2 for 24 hours and PPME1 mRNA expression was analyzed by RT-qPCR. For each cell line, the expression of PPME1 mRNA was normalized to the mean result for EMT6 cells at 20% O_2. $^{****}P < 0.0001$, two-way ANOVA with Tukey's multiple comparisons test. (**B**) Mouse BC cell lines were exposed to 20% or 1% O_2 for 48 hours and the expression of PPME1 protein and β-actin was determined by immunoblot assays. (**C** and **D**) MDA-MB-231 (**C**) and MCF7 (**D**) subclones were exposed to 20% or 1% O_2 for 24 hours, followed by RT-qPCR assays; ns, not significant, two-way ANOVA with Tukey's multiple comparisons test. (**E**) BALB/c mice with 150-mm^3 EMT6 tumors were treated with vehicle or either of two dual HIF-1/2 inhibitors by intraperitoneal injection (40 mg/kg twice daily) for 5 days. Tumors were harvested 4 hours after the last dose and tissue lysates were prepared for immunoblot assays. (**F** and **G**) MDA-MB-231 (**F**) and MCF7 (**G**) cells were exposed to 20% or 1% O_2 for 16 hours, and ChIP-qPCR assays were performed using antibodies against HIF-1α, HIF-1β or HIF-2α. An *RPL13A* sequence was amplified by qPCR; ns, not significant, two-tailed Student's t-test. In all bar graphs, data are presented as mean ± SD (n = 3). (**H**) Gene Set Enrichment Analysis was performed to compare the PI3K-AKT signaling pathway in the top 200 TCGA BCs ranked by PPME1 expression with the bottom 200. NES, normalized enrichment score. (**I**) Correlation analysis using Pearson's test between hypoxia and PIK3CA pathway scores in TCGA BCs.

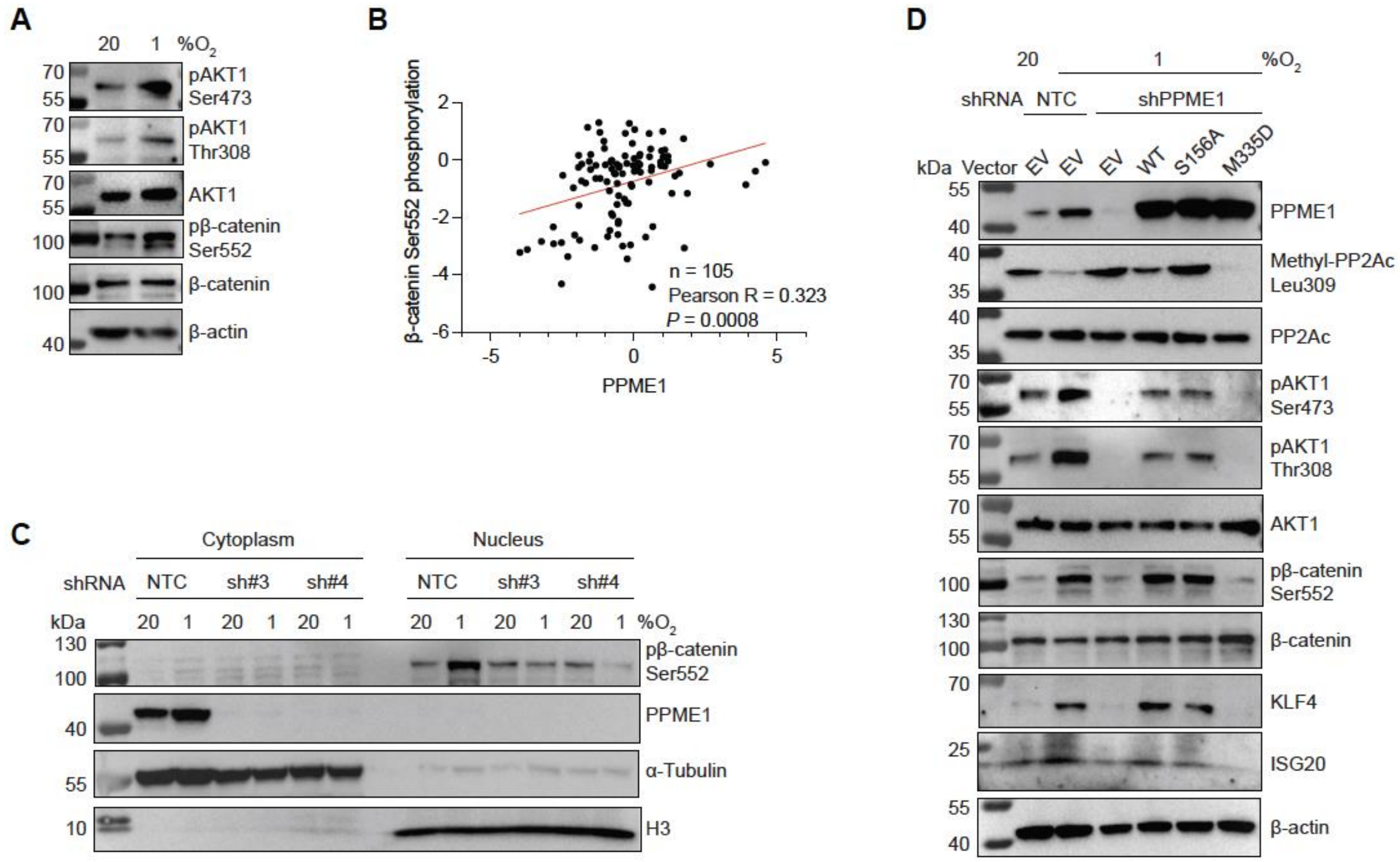

Supplemental Figure 2. PPME1 activates AKT–β-catenin signaling via direct PP2Ac catalytic inhibition independently of its demethylase activity. (**A**) MCF10A cells were exposed to 20% or 1% O_2 for 48 hours, followed by immunoblot assays. (**B**) Pearson correlation analysis between PPME1 protein expression and phosphorylated β-catenin at Ser552 using a published proteomic dataset. (**C**) MDA-MB-231 subclones expressing NTC or PPME1 shRNA (#3 or #4) were exposed to 20% or 1% O_2 for 48 hours, followed by cytoplasmic and nuclear fractionation and immunoblot assays. (**D**) MDA-MB-231 NTC or shPPME1 subclones were transduced with empty vector (EV) or vector encoding wild-type PPME1 (WT) or the indicated PPME1 mutant, exposed to 20% or 1% O_2 for 48 hours, and analyzed by immunoblot assays.

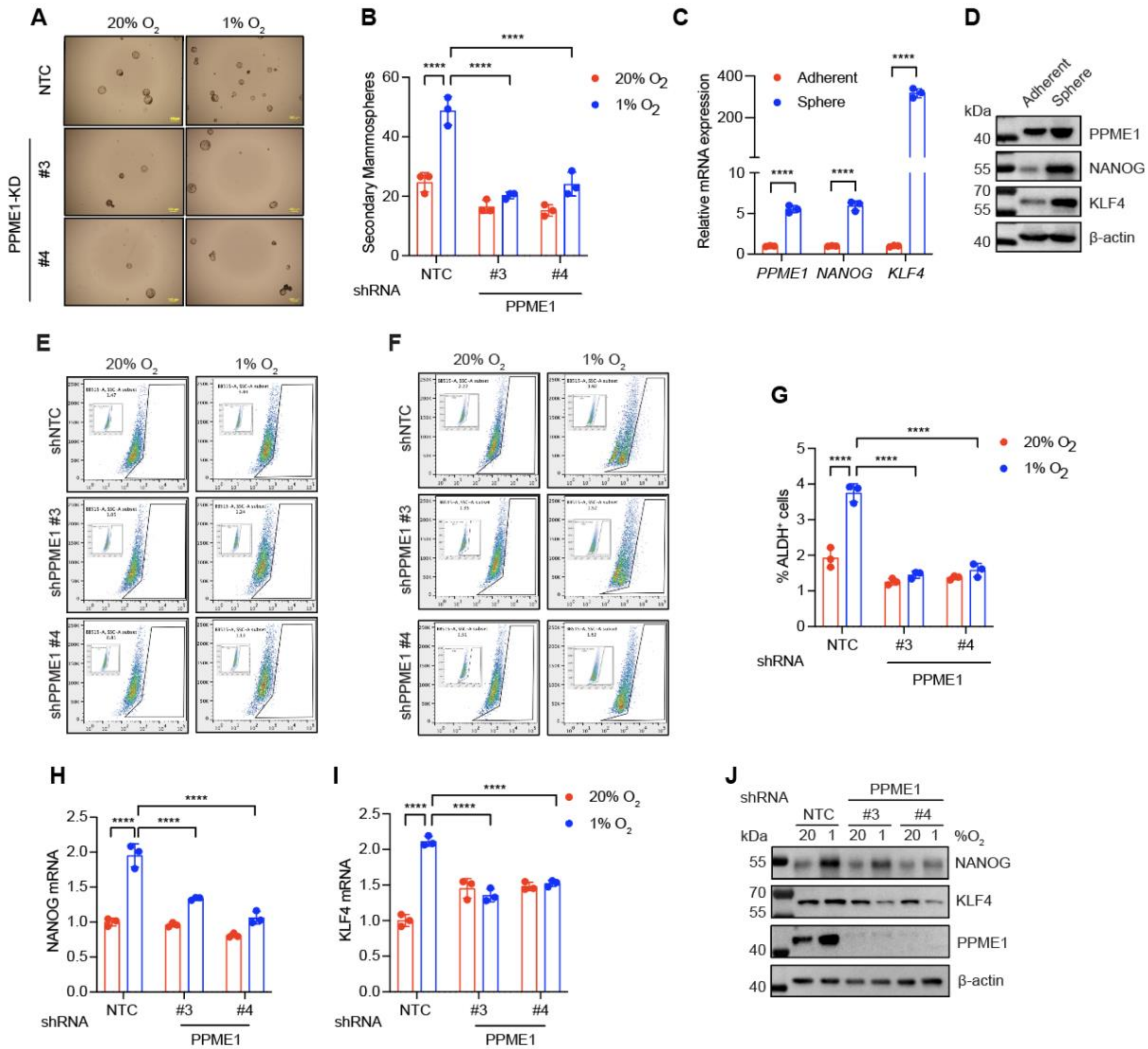

Supplemental Figure 3. PPME1 is required for hypoxia-induced BCSC enrichment. (**A** and **B**) Representative light microscope images (**A**; scale bar: 100 μm) and quantification (**B**) of 7-day secondary mammospheres obtained after dispersion of 7-day primary mammospheres grown from MCF7 subclones expressing NTC or PPME1 shRNA (#3 or #4) that were exposed to 20% or 1% O_2 for 72 hours. $^{****}P < 0.0001$, two-way ANOVA with Tukey's multiple comparisons test. (**C** and **D**) mRNA and protein were extracted from MCF7 cells grown as an adherent monolayer or from secondary mammospheres, followed by RT-qPCR (**C**) and immunoblot assays (**D**), respectively. RT-qPCR data were normalized to adherent cells. $^{****}P < 0.0001$, two-tailed Student's *t* test. (**E** and **F**) Flow cytometry gating of $ALDH^+$ cells in MDA-MB-231 (**E**) and MCF7 (**F**) subclones. (**G**) MCF7 subclones were exposed to 20% or 1% O_2 for 72 hours, and the percentage of $ALDH^+$ cells was determined. $^{****}P < 0.0001$, two-way ANOVA with Tukey's multiple comparisons test. (**H-J**) MCF7 subclones were exposed to 20% or 1% O_2 for 24 or 48 hours, followed by RT-qPCR (**H** and **I**) and immunoblot assays (**J**), respectively. RT-qPCR data were normalized to NTC at 20% O_2. $^{****}P < 0.0001$, two-way ANOVA with Tukey's multiple comparisons test. In all bar graphs, data are presented as mean ± SD (n = 3).

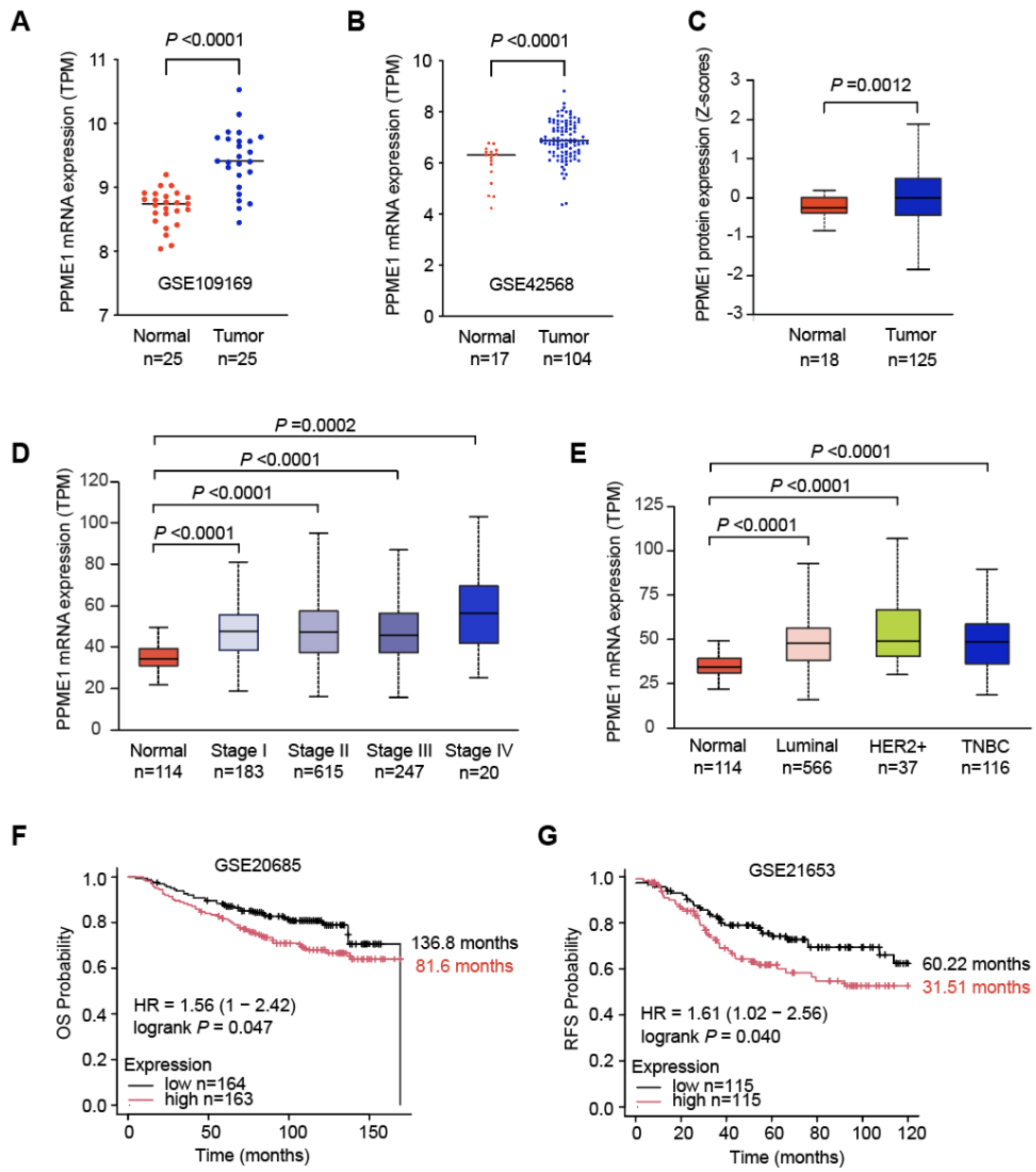

Supplemental Figure 4. PPME1 expression correlates with adverse clinical outcomes in BC patients. (**A**) PPME1 mRNA expression in normal breast tissue and BC samples from Gene Expression Omnibus (GEO) dataset GSE109169 were analyzed by Student's *t* test. (**B**) PPME1 mRNA expression in normal breast tissue and BC samples from GEO dataset GSE42568 were analyzed using unpaired Student's *t* test. (**C**) *Z*-scores for PPME1 protein expression in normal breast tissue and BC samples from the Clinical Proteomic Tumor Analysis Consortium were analyzed using unpaired Student's *t* test. (**D**) PPME1 mRNA expression in normal and BC samples stratified according to cancer stage were analyzed by one-way ANOVA with Tukey's multiple comparisons test. (**E**) PPME1 mRNA expression in normal and BC samples from different clinical subtypes were analyzed by one-way ANOVA with Tukey's multiple comparisons test. (**F** and **G**) BC samples from the GSE20685 (**F**) and GSE21653 (**G**) datasets were stratified based on PPME1 mRNA expression in primary tumors into high (red) and low (black) expression groups relative to the median. Kaplan–Meier analyses were performed to evaluate overall survival (OS; **F**) and relapse-free survival (RFS; **G**) between the two groups. The hazard ratio (HR), *P* value by log-rank test, and median survival time are shown.

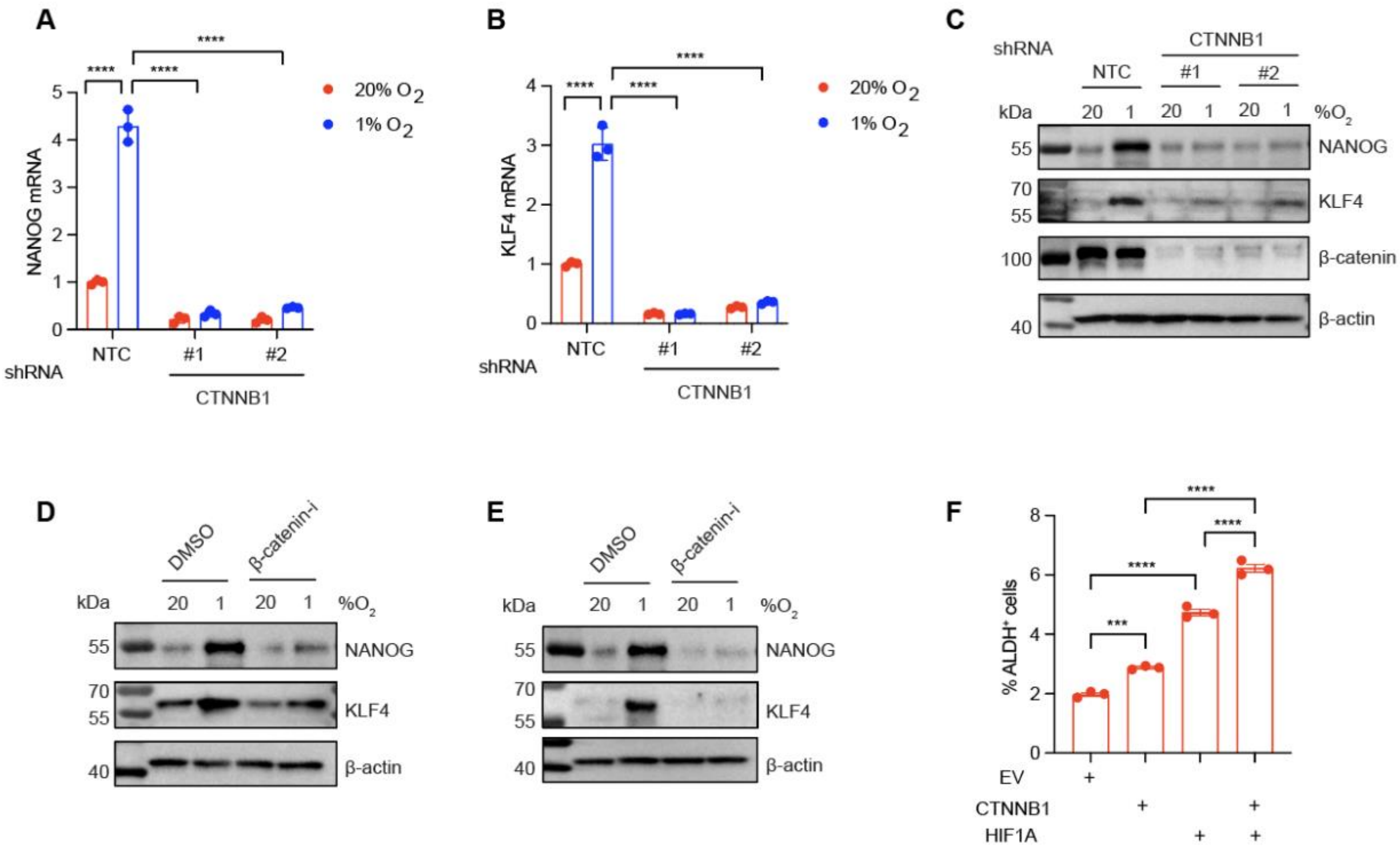

Supplemental Figure 5. β-catenin is required for hypoxia-induced BCSC enrichment in association with HIF-1. (**A** and **B**) MCF7 subclones expressing NTC or β-catenin shRNA (#1 or #2) were exposed to 20% or 1% O_2 for 24 hours, followed by RT-qPCR using primers specific for NANOG (**A**) or KLF4 (**B**) mRNA, which was normalized to NTC at 20% O_2. $^{****}P < 0.0001$, two-way ANOVA with Tukey's multiple comparisons test. (**C**) MCF7 subclones were exposed to 20% or 1% O_2 for 48 hours, followed by immunoblot assays. (**D** and **E**) MDA-MB-231 (**D**) and MCF7 (**E**) cells were treated with DMSO vehicle or β-catenin inhibitor MSAB (5 μM) and exposed to 20% or 1% O_2 for 48 hours, followed by immunoblot assays. (**F**) MDA-MB-231 cells were transfected with empty vector (EV) or expression vector encoding β-catenin or HIF-1α. The percentage of $ALDH^+$ cells was determined 48 hours after transfection. $^{***}P < 0.001$, $^{****}P < 0.0001$, one-way ANOVA with Tukey's multiple comparisons test. In all bar graphs, data are presented as mean ± SD (n = 3).

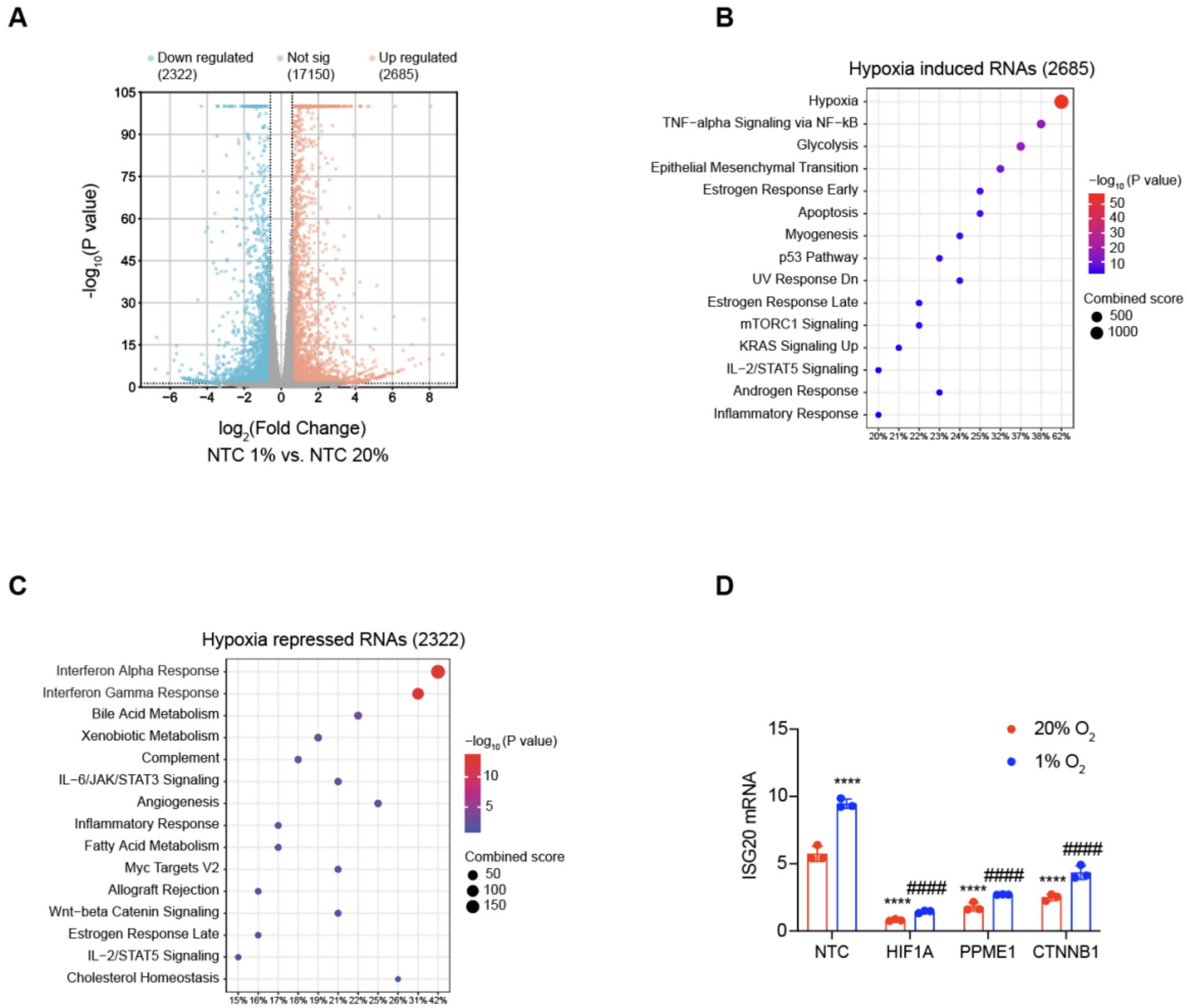

Supplemental Figure 6. HIF-1, PPME1, and β-catenin are required for a broad transcriptional response to hypoxia. (**A**) Differential gene expression from RNA-seq analysis in NTC cells exposed to 1% vs. 20% O_2 is presented as a volcano plot. RNAs with fold change > 1.5 and FDR < 0.05 are shown in blue (hypoxia-repressed) or red (hypoxia-induced). (**B** and **C**) Bubble plots depicting the pathway enrichment analysis of hypoxia-induced (**B**) and hypoxia-repressed (**C**) RNAs. The color scale represents the – $\log_{10}$ transformed *P* value (FDR); the size of each bubble corresponds to the combined score; and the *x*-axis indicates the proportion of genes within each pathway that show significant differential expression. (**D**) ISG20 expression across all groups from RNA-seq analysis is shown. $^{****}P < 0.0001$ versus NTC at 20% O_2; $^{\#\#\#\#}P < 0.0001$ versus NTC at 1% O_2, two-way ANOVA with Tukey's multiple comparisons test. Data are presented as mean ± SD (n = 3).

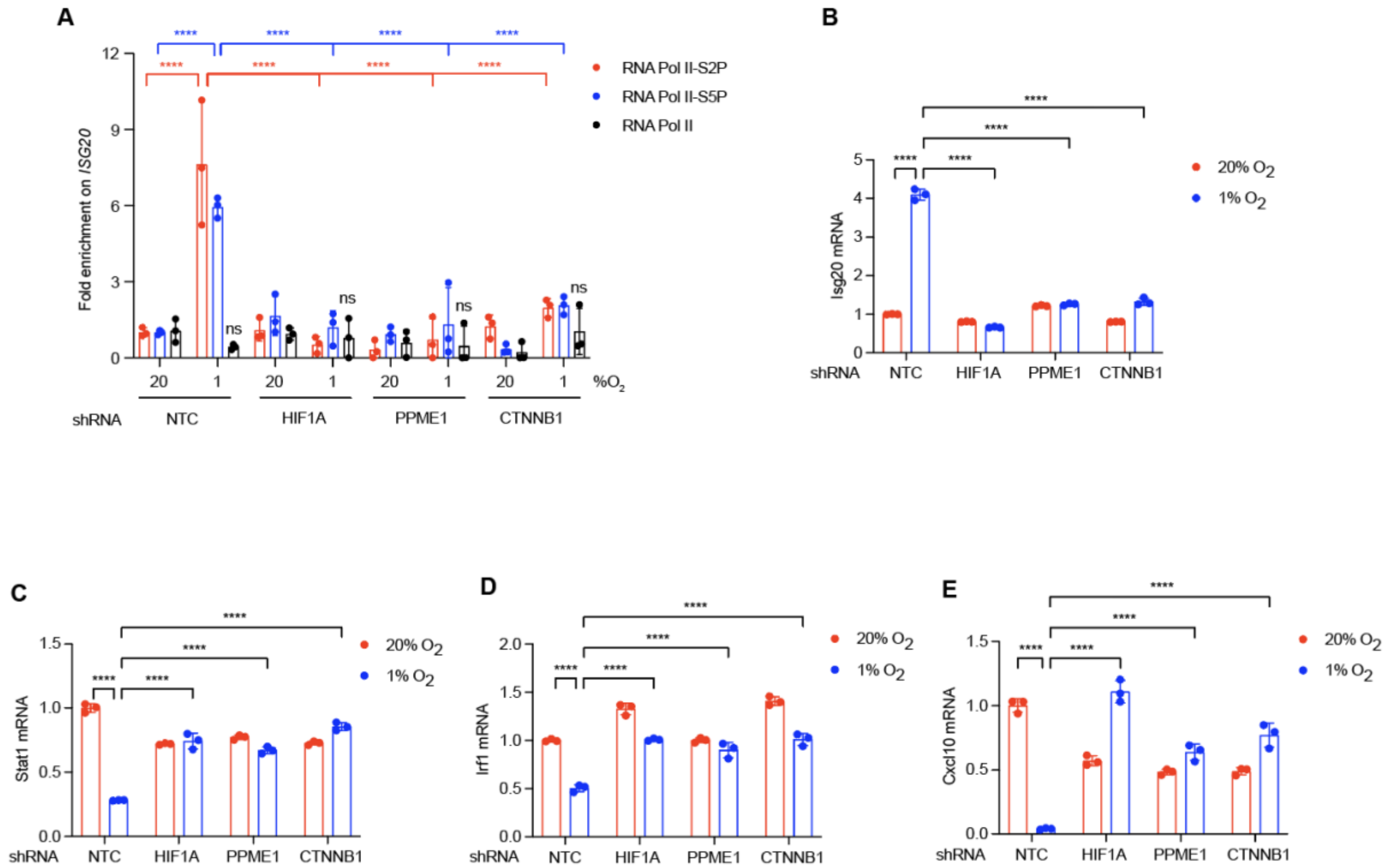

Supplemental Figure 7. PPME1 and β-catenin are required for *ISG20* transcription initiation and elongation. (**A**) MDA-MB-231 subclones were exposed to 20% or 1% O_2 for 16 hours, and ChIP was performed using antibody against RNA polymerase II phosphorylated on the carboxyl-terminal domain of the large subunit at serine-5 (RNA Pol II-S5P) or serine-2 (RNA Pol II-S2P), or total RNA Pol II. Primers encompassing HIF-1 binding sites in the *ISG20* gene were used for qPCR. $^{****}P < 0.0001$, two-way ANOVA with Tukey's multiple comparisons test. (**B**-**E**) EMT6 subclones were exposed to 20% or 1% O_2 for 48 hours, followed by RT-qPCR. Results were normalized to NTC at 20% O_2, $^{****}P < 0.0001$, two-way ANOVA with Tukey's multiple comparisons test. For all bar graphs, data are presented as mean ± SD (n = 3).

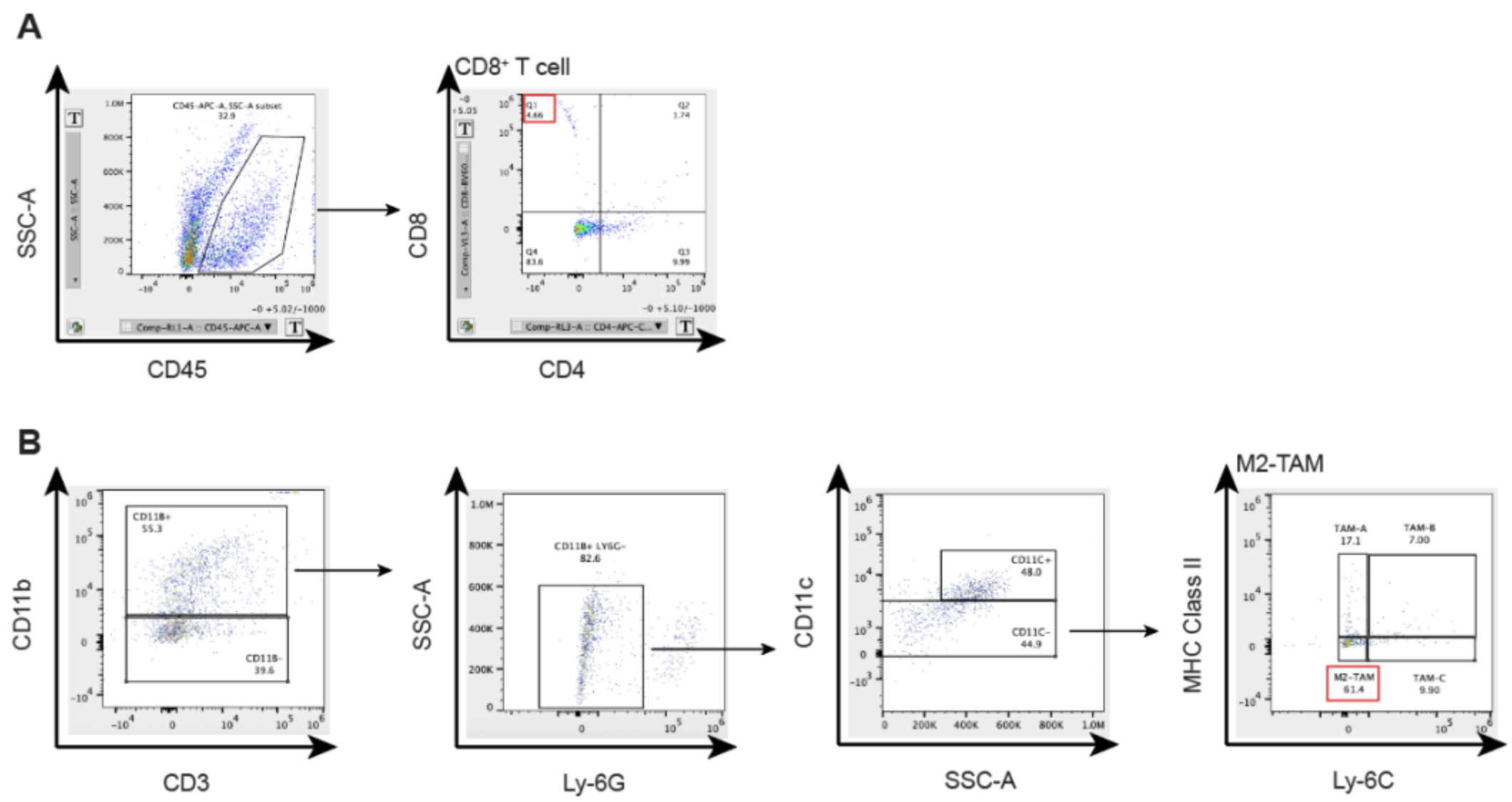

Supplemental Figure 8. Analysis of immune cells in EMT6 tumors. (**A** and **B**) Flow cytometry gating strategy used to identify CD8^{+} T cells (**A**) and M2-TAMs **(B**).

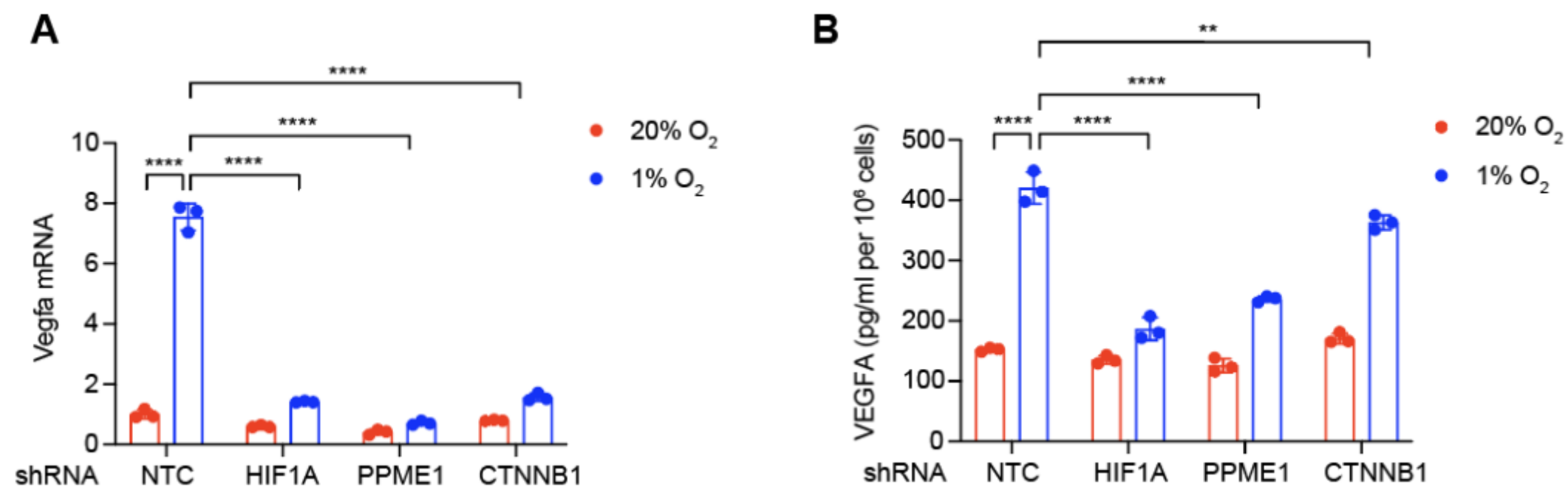

Supplemental Figure 9. Analysis of VEGFA expression in cultured cells. (**A** and **B**) EMT6 subclones were exposed to 20% or 1% O_2 for 24 hours. Total RNA was extracted for RT-qPCR analysis of VEGFA mRNA levels (**A**). RT-qPCR data were normalized to NTC at 20% O_2. $^{****}P < 0.0001$, two-way ANOVA with Tukey's multiple comparisons test. VEGFA protein levels in the culture supernatant were measured by ELISA (**B**). VEGFA protein concentration was normalized to the cell number. $^{**}P < 0.01$, $^{****}P < 0.0001$, two-way ANOVA with Tukey's multiple comparisons test. In both bar graphs, data are presented as mean ± SD (n = 3).

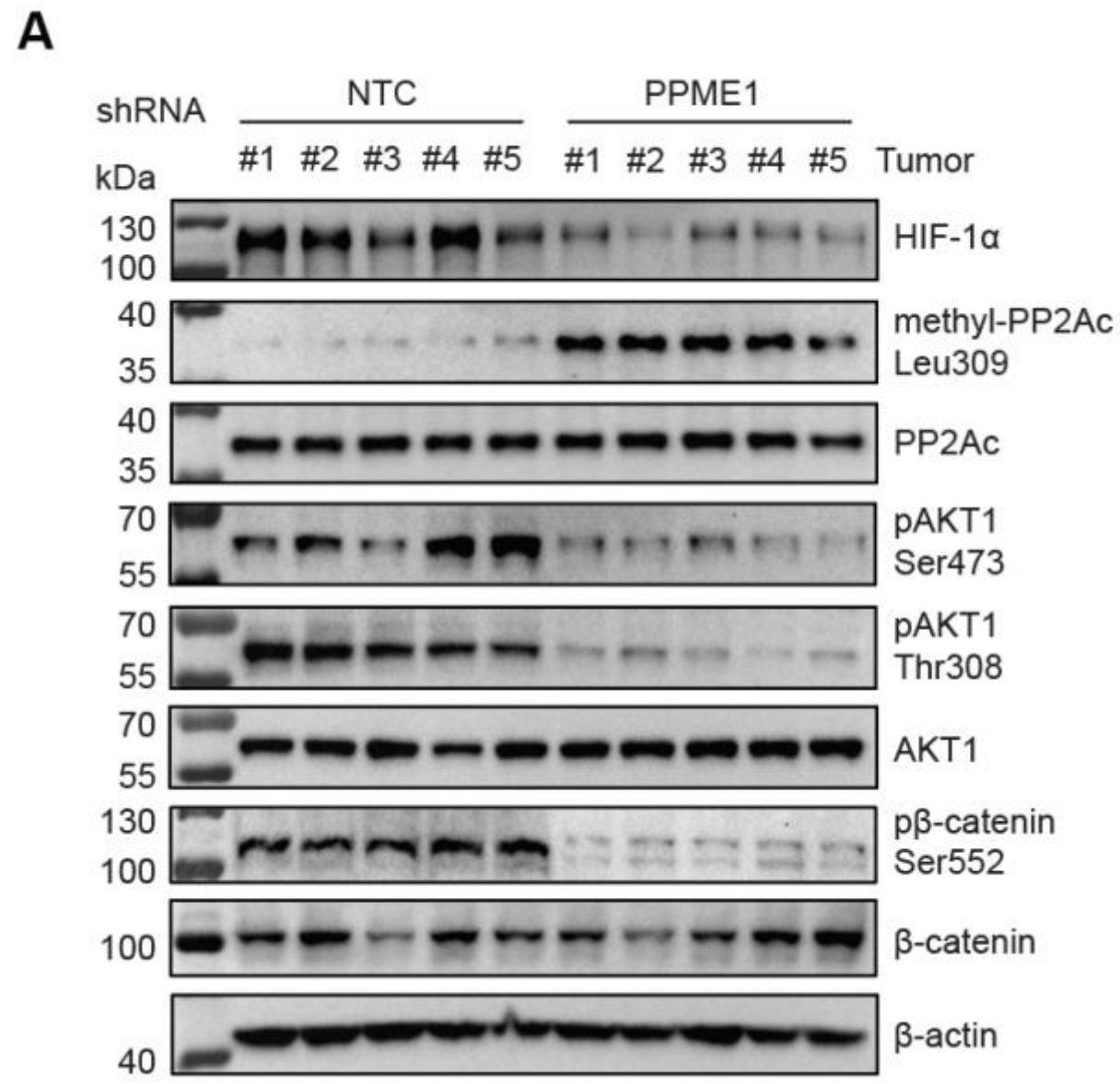

Supplemental Figure 10. Analysis of the AKT–β-catenin signaling axis in NTC and PPME1-KD tumors. (**A**) Protein was extracted from tumor tissues obtained from the animal experiment shown in Figure 8 and immunoblot assays were performed.

Supplemental Methods

Cell culture. The culture medium for EMT6 cells was supplemented with 15% (vol/vol) FBS, 2 mM L-glutamine and 1% (vol/vol) penicillin-streptomycin. Other culture media were supplemented with 10% FBS and 1% penicillin-streptomycin. Cells were subjected to hypoxia in a controlled-atmosphere chamber (Plas Labs, Lansing, MI, USA) flushed with a gas mixture of 1% O_2, 5% CO_2, and 94% N_2. Cell lines were mycoplasma-free and authenticated by short tandem repeat DNA profiling analysis by the Johns Hopkins Genetics Resources Core Facility (JHGRCF).

Lentiviral transduction. All lentiviral vectors were co-transfected into HEK293T cells with packaging plasmids pCMVΔR8.9 and pCMV-VSV-G using PolyJet. Viruses were harvested 48 hours after transfection and passed through a 0.45-µm filter. BC cells were transduced with viral supernatant for 48 hours, and stably transduced cells were selected with puromycin at a concentration of 0.5 (MDA-MB-231) or 1.0 (MCF7, SUM159 and EMT6) µg/mL.

ChIP-seq data analysis. FastQ files were quality checked and adapter trimmed using Trim-Galore (v0.6.6) as described before (1). ChIP-seq peaks were called using MACS2 in narrow peak mode with input samples as controls, and peaks were overlaid and subsetted using Bedtools (v2.30.0). Differentially bound peaks were identified using the DiffBind package (v3.8.4) in R (v4.2). Genes with differential HIF-1α binding were identified using Gencode gene annotations and visualized using Integrative Genomics Viewer genome browser.

ChIP. Chromatin was sheared by sonication to fragments of 200 to 1000 bp. Lysates were diluted 1:10 with ChIP dilution buffer (0.01% SDS, 1.1% Triton X-100, 1.2 mM EDTA, 16.7 mM Tris-HCl [pH 8.1], 167 mM NaCl), precleared with salmon sperm DNA/protein A-agarose beads

(MilliporeSigma) at 4 °C for 1 hour, and incubated with antibody in the presence of protein A-agarose beads overnight. After sequential washes with low-salt (0.1% SDS, 1% Triton X-100, 2 mM EDTA, 20 mM Tris-HCl [pH 8.1], 150 mM NaCl), high-salt (0.1% SDS, 1% Triton X-100, 2 mM EDTA, 20 mM Tris-HCl [pH 8.1], 500 mM NaCl), LiCl (0.25 M LiCl, 1% IGEPAL-CA630, 1% deoxycholic acid, 1 mM EDTA, 10 mM Tris [pH 8.1]), and Tris-EDTA (10 mM Tris-HCl [pH 8.0], 1 mM EDTA) buffers, DNA was recovered in elution buffer (1% SDS, 0.1 M $NaHCO_3$), and cross-links were reversed by addition of 0.2 M NaCl at 65°C for 4 hours. DNA was purified by phenol-chloroform extraction and ethanol precipitation and analyzed by qPCR.

Nuclear/cytoplasmic fractionation. Cultured cells or dissociated tumor cells were gently resuspended in Buffer A (10 mM HEPES, 1.5 mM $MgCl_2$, 10 mM KCl, 0.5 mM DTT, and 0.05% NP-40, pH 7.9), incubated on ice for 15 min, and centrifuged at 3,000 rpm for 10 min at 4°C. The supernatant was collected as the cytoplasmic fraction. The pellet was resuspended in Buffer B (5 mM HEPES, 1.5 mM $MgCl_2$, 0.2 mM EDTA, 0.5 mM DTT, 26% [vol/vol] glycerol, and 300 mM NaCl, pH 7.9) and homogenized with 30 strokes in a glass Dounce homogenizer on ice. The lysate was incubated on ice for 30 min and then centrifuged at 14,000 rpm for 20 min at 4°C. The supernatant was collected as the nuclear fraction.

ALDH assay. Cultured cells were trypsinized and filtered through a 70-µm cell strainer. Cells were counted and 1×10^6 live cells were suspended in assay buffer containing 1 mM BODIPY-aminoacetaldehyde and incubated at 37°C for 45 minutes. An aliquot of cells from each sample was treated with 50 mM diethylaminobenzaldehyde, an ALDH inhibitor, as a negative control for gating. Samples were analyzed by flow cytometry using FACSCalibur (BD Biosciences).

RNA-seq. Library preparation and sequencing using the NovaSeq 6000 platform (Illumina) were performed by the Johns Hopkins Genetics Resources Core Facility High-Throughput Sequencing Center. RNA-seq data were processed and interpreted using Genialis Expressions software (https://www.genialis.com). The automated data analysis on the Genialis platform consisted of the following steps: sequence quality checks were performed on raw and trimmed reads (FastQC), trimming and quality filtering of reads (BBDuk), mapping to reference human genome Ensembl v.92 (STAR), expression quantification (featureCounts), and expression normalization (RNA.norm). Key QC metrics (e.g., mapping statistics) were collected. As an additional quality control step, a sample of one million reads (Seqtk tool) was mapped (STAR) separately to human rRNA and globin sequences to evaluate the proportion of these reads in the sample. Differential gene expression analyses were performed with DESeq2. Lowly-expressed genes, which have expression count summed over all samples below 10, were filtered out from the differential expression analysis input matrix. Differential expression results (1% versus 20% O_2) with FDR < 0.05 and an absolute fold change (FC) > 1.5 were used as cutoff for identification of hypoxia-induced and hypoxia-repressed genes. We also compared different subclones (KD versus NTC) under hypoxic conditions to identify significantly changed mRNA expression (absolute FC > 1.5 and FDR < 0.05). The combined list of mRNAs identified by these two strategies was used for Venn and gene ontology (GO) analysis. RNA-seq data were visualized using SRplot for Venn diagrams, volcano plots and bubble plots (2) and Heatmapper for heatmaps (3).

Animal studies. Cancer cells were trypsinized, rinsed with PBS and injected into mice in a 1:1 (vol/vol) suspension of Matrigel (BD Biosciences) in PBS. MDA-MB-231 (1000 cells) or EMT6 (2.5×10^5 cells) subclones were injected into the mammary fat pad of SCID (Charles River, strain 561) or BALB/c (Charles River, strain 028) mice, respectively. Primary tumors were

measured with calipers to determine the long (L) and short (S) axis by a blinded observer and tumor volume (V) was calculated as $V = L \times S^2 \times 0.5$.

ELISA. Tumor homogenates were centrifuged at 10,000 × g for 20 minutes at 4 °C to pellet debris, and the clarified supernatants (or aliquots of conditioned media from cultured cells) were collected for cytokine analysis according to the manufacturers' instructions. Optical density was measured at 450 nm using a VICTOR Nivo plate reader. Sample concentrations were determined from analyte-specific standard curves generated for each assay.

Statistics. All statistical analyses were performed using Prism 9 (GraphPad Software). Comparisons between two groups were assessed using the Mann–Whitney test, Welch's t test, or two-tailed Student's t test, as appropriate. Differences among multiple groups were analyzed using 1-way or 2-way ANOVA, as indicated in the corresponding figure legends, followed by the indicated multiple-comparisons test. Associations between variables were evaluated using Pearson's test. For Kaplan–Meier survival analysis, patients were stratified based on the median mRNA expression level, and survival curves were analyzed using the log-rank test through the KMplot online tool (https://kmplot.com/analysis/). For animal experiments, sample sizes were determined based on pilot data and previous studies using orthotopic breast tumor models, with the goal of providing at least 80% power to detect biologically meaningful differences in tumor growth, tumor weight, or tumor-infiltrating immune cell populations at a two-sided α level of 0.05, while minimizing animal use in accordance with institutional animal care guidelines. The exact sample size for each experiment is indicated in the corresponding figure legend.

Public Database Analysis. The TCGA BRCA dataset was accessed through the UCSC Xena browser (https://xenabrowser.net/) (4). Gene set enrichment analysis was performed using

GSEA version 2.1.0 software downloaded from the Broad Institute (https://www.gsea-msigdb.org/gsea/index.jsp) using the MSigDB 3.0 gene set signature database (5) following the author's instructions. PPME1 mRNA expression in BC patients was further analyzed using GEO datasets GSE109169 and GSE42568. mRNA and protein expression of PPME1 in tumor versus normal breast tissues were evaluated using the UALCAN database (https://ualcan.path.uab.edu/) (6). Kaplan–Meier survival analyses were performed using BC patient datasets integrating gene expression and clinical outcome data, obtained from the KMplot database (https://kmplot.com/analysis/) (7).

References

1. Yang Y, et al. HIF-1 promotes murine breast cancer brain metastasis by increasing production of integrin β3-containing extracellular vesicles. *J Clin Invest.* 2025;135(14).
2. Tang D, et al. SRplot: A free online platform for data visualization and graphing. *PLoS One.* 2023;18(11):e0294236.
3. Babicki S, et al. Heatmapper: web-enabled heat mapping for all. *Nucleic Acids Res.* 2016;44(W1):W147–153.
4. Goldman MJ, et al. Visualizing and interpreting cancer genomics data via the Xena platform. *Nat Biotechnol.* 2020;38(6):675–678.
5. Liberzon A, et al. Molecular signatures database (MSigDB) 3.0. *Bioinformatics.* 2011;27(12):1739–1740.
6. Chandrashekar DS, et al. UALCAN: A portal for facilitating tumor subgroup gene expression and survival analyses. *Neoplasia.* 2017;19(8):649–658.
7. Lánczky A, and Győrffy B. Web-based survival analysis tool tailored for medical research (KMplot): development and implementation. *J Med Internet Res.* 2021;23(7):e27633.
