# Supplementary material for: HIF-1–mediated PPME1 expression promotes breast cancer progression via AKT activation and β-catenin recruitment
Source: J Clin Invest. 2026 Oct 1;136(19):e205415. doi: 10.1172/JCI205415 (PMC13626848; doi:10.1172/JCI205415)

Figure1

Full unedited blot/gel for Figure1B

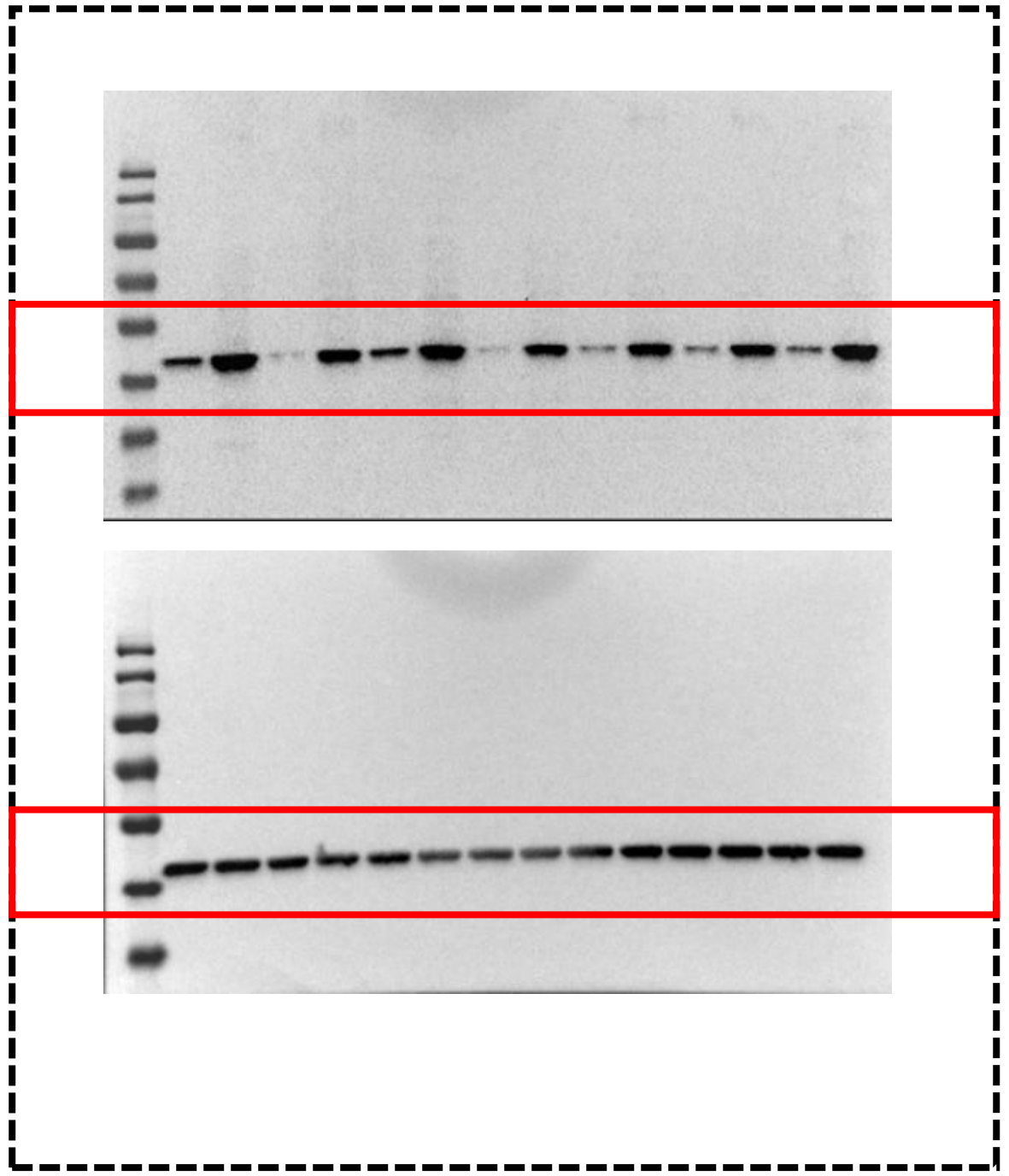

Full unedited blot/gel for Figure1E

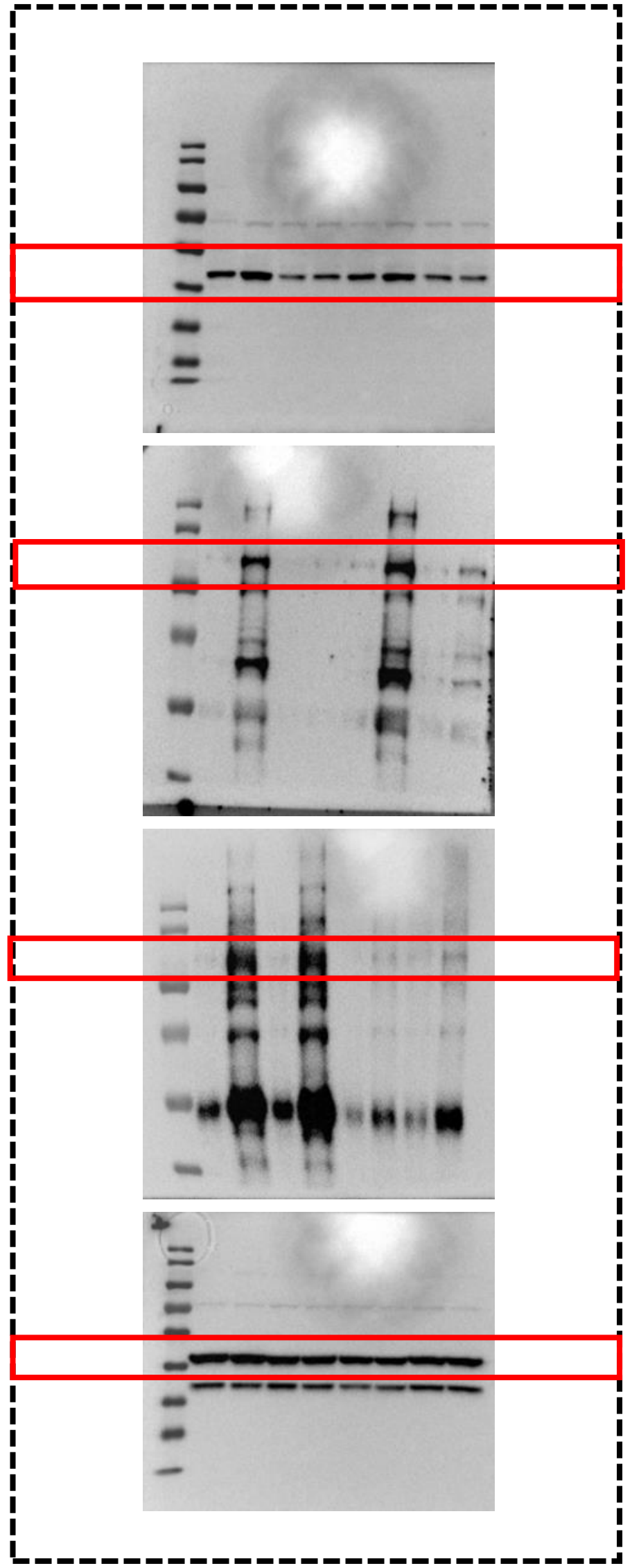

Full unedited blot/gel for Figure1G

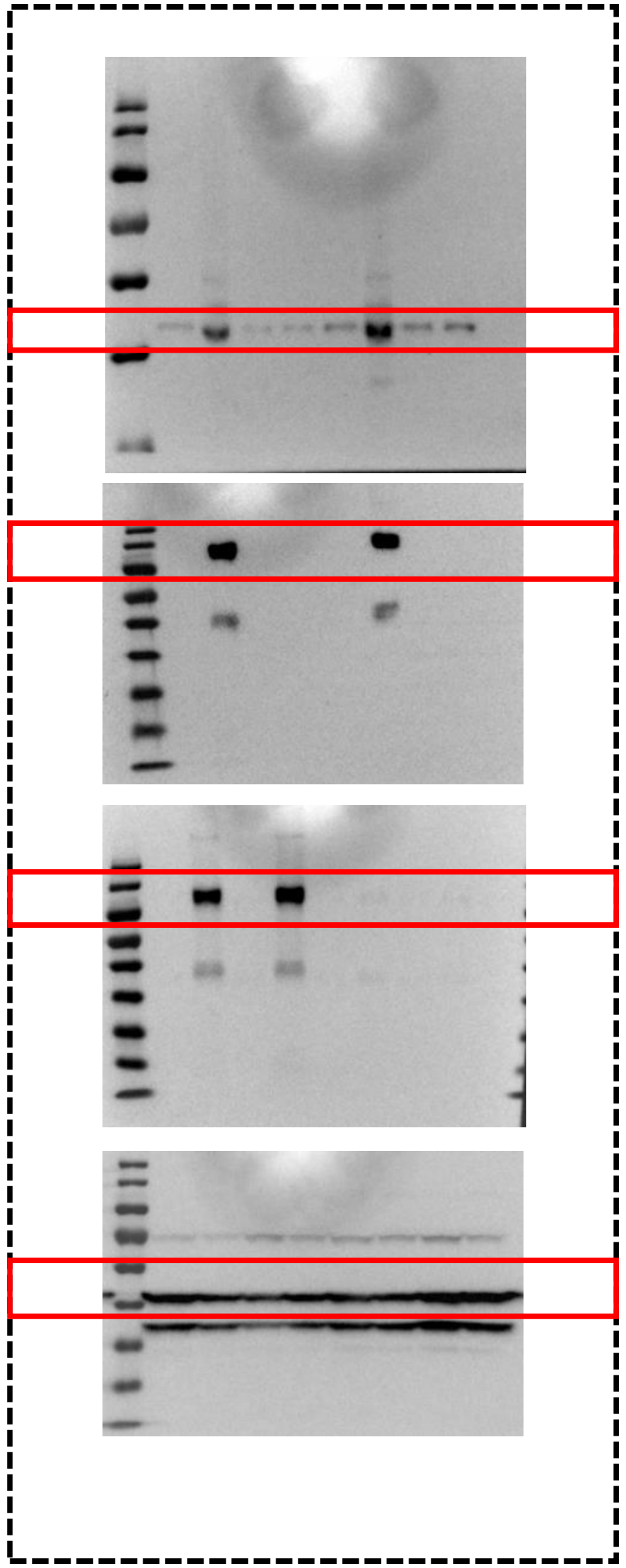

Figure2

Full unedited blot/gel for Figure2A

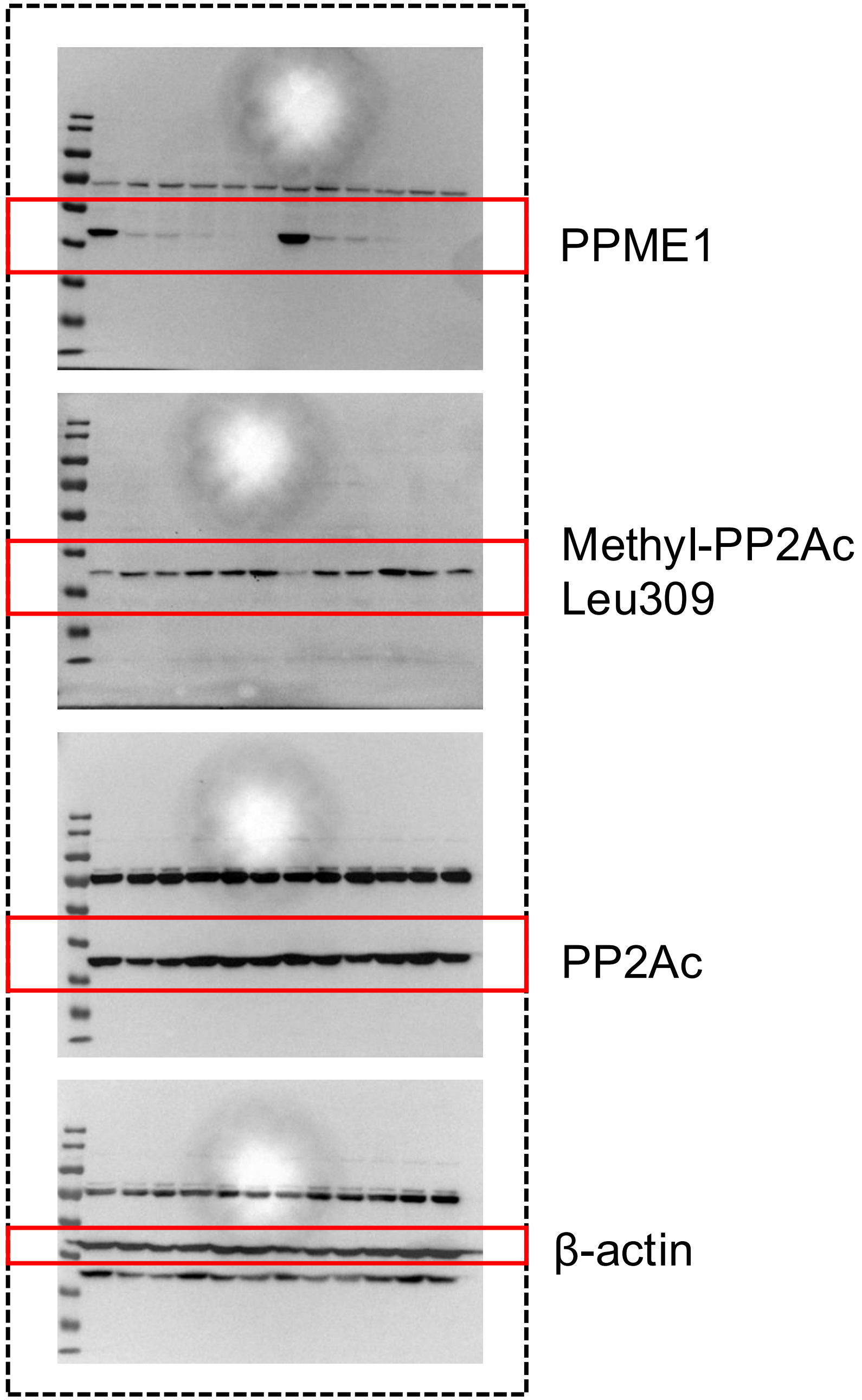

Full unedited blot/gel for Figure2C

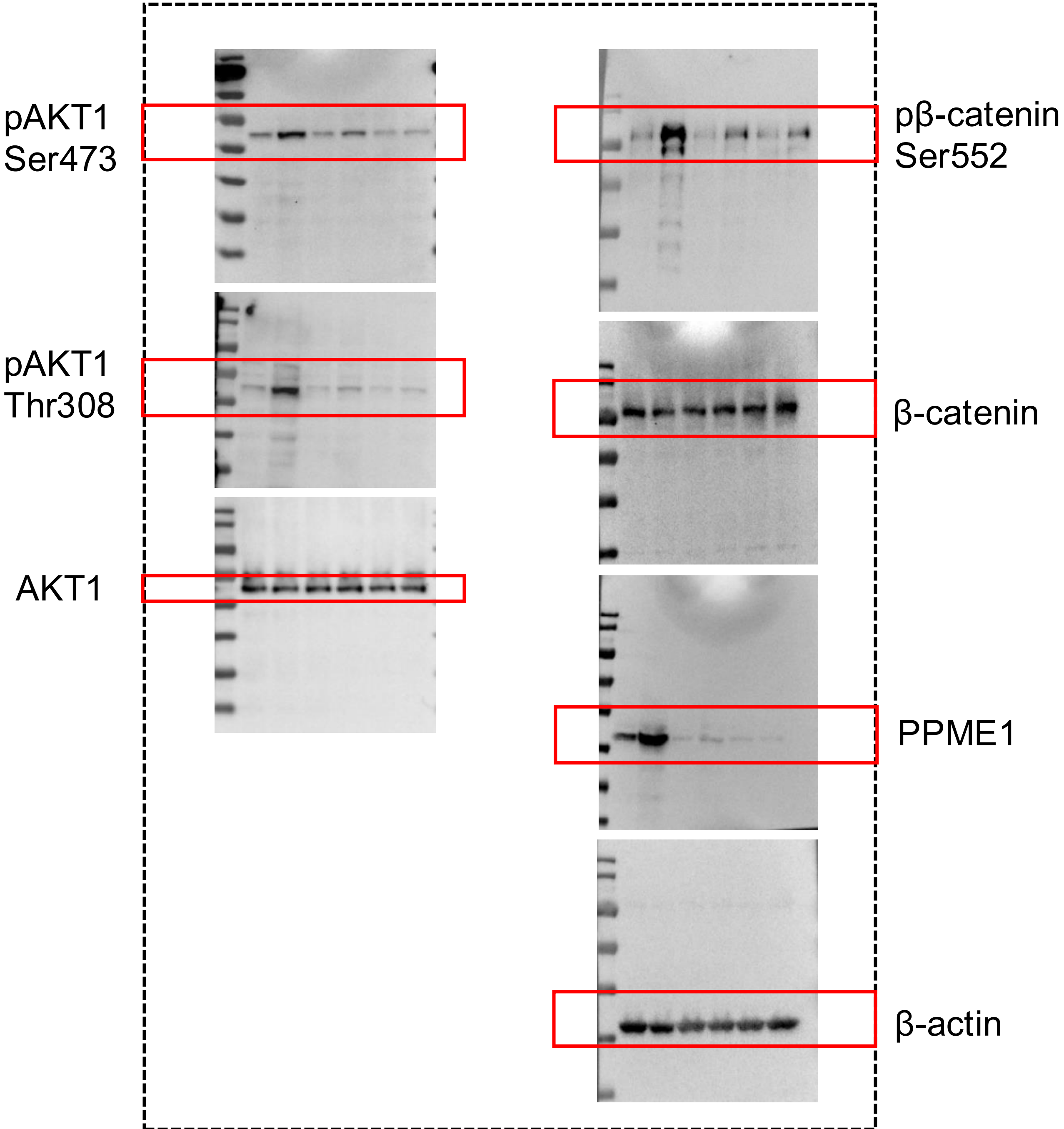

Figure2

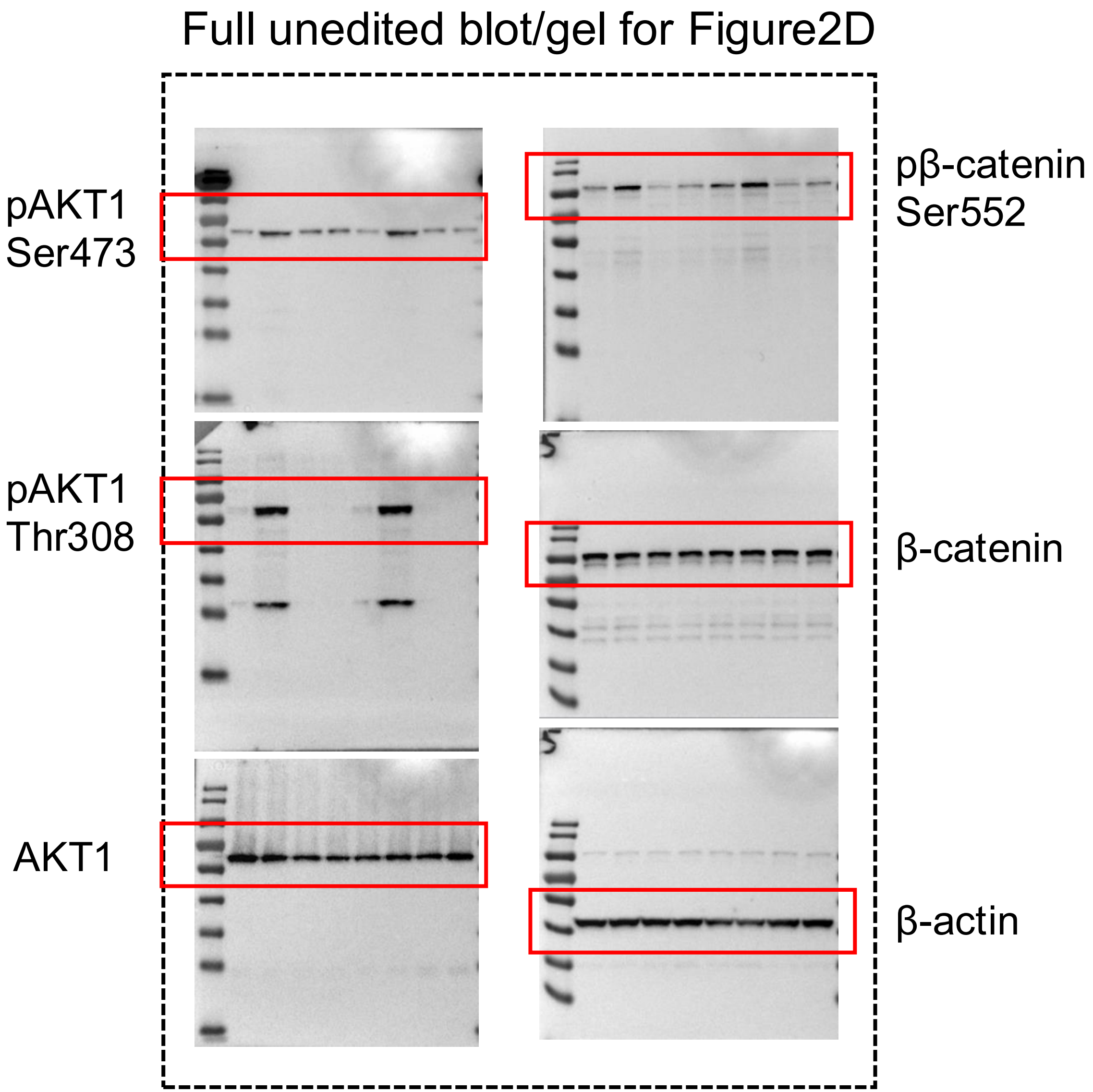
Full unedited blot/gel for Figure2D
pAKT1
Ser473
pAKT1
Thr308
AKT1
pβ-catenin
Ser552
β-catenin
β-actin

Figure2

Full unedited blot/gel for Figure2E

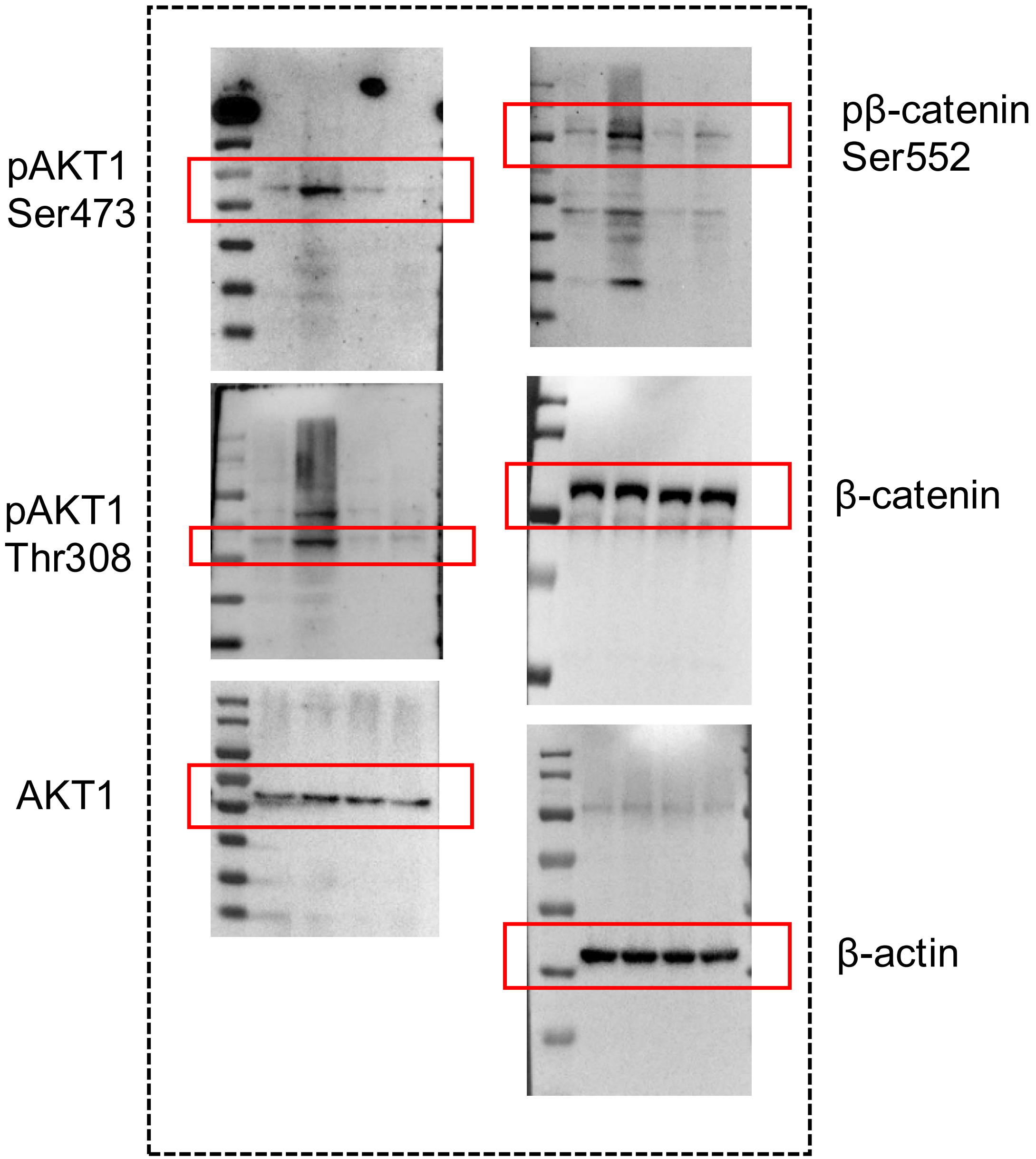

Full unedited blot/gel for Figure2F

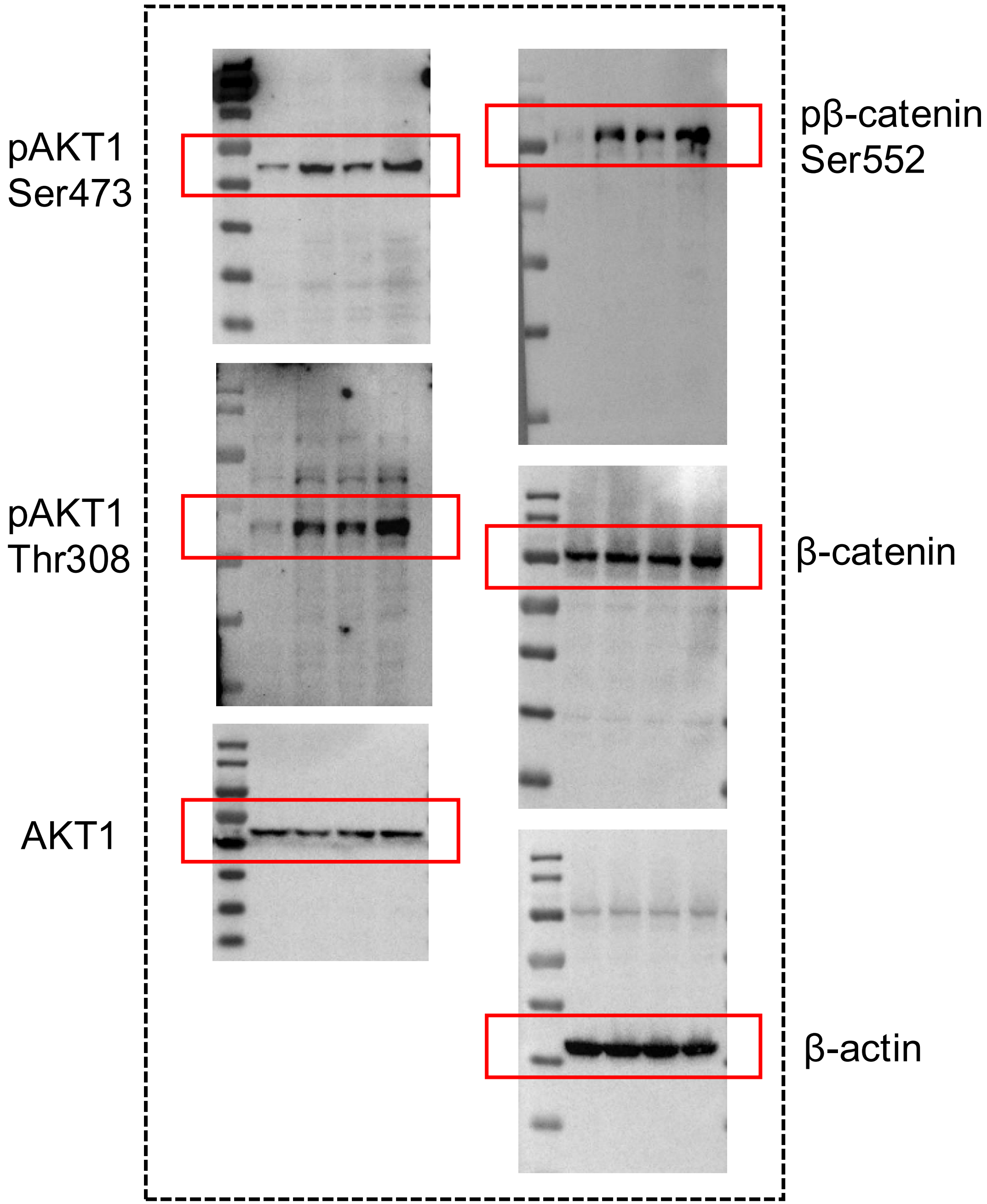

Figure3

Full unedited blot/gel for Figure3E

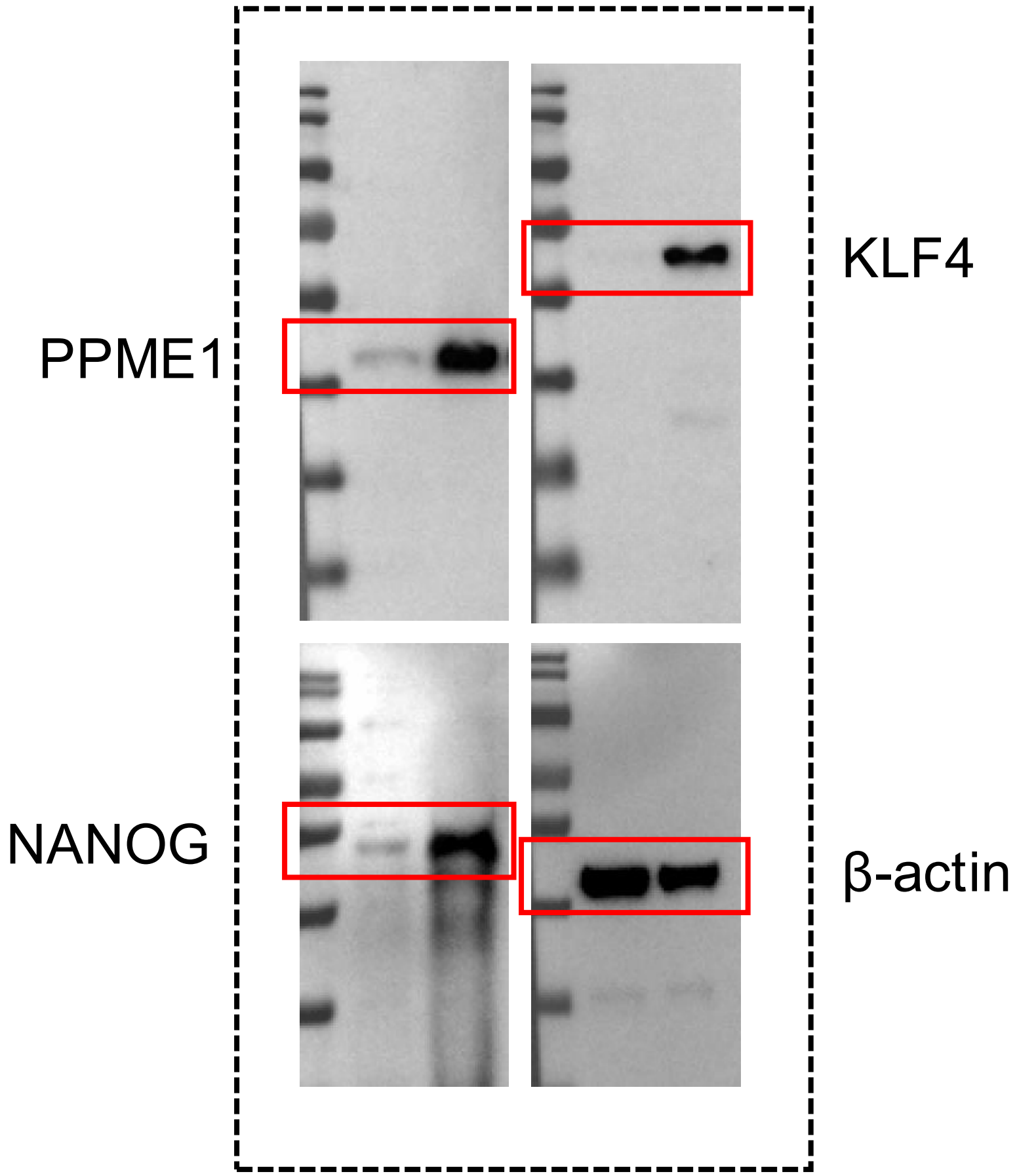

Full unedited blot/gel for Figure3I

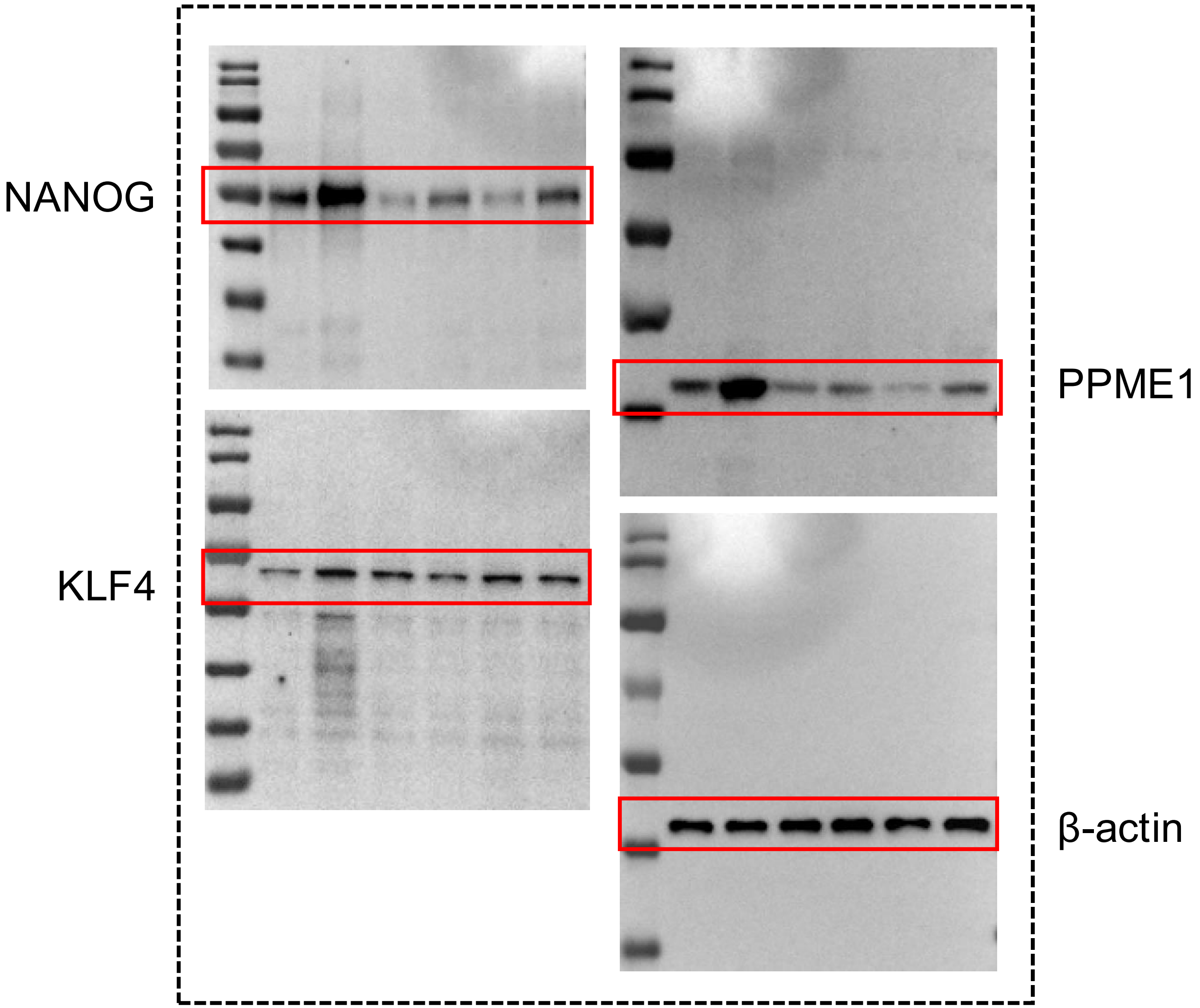

Figure4

Full unedited blot/gel for Figure4G

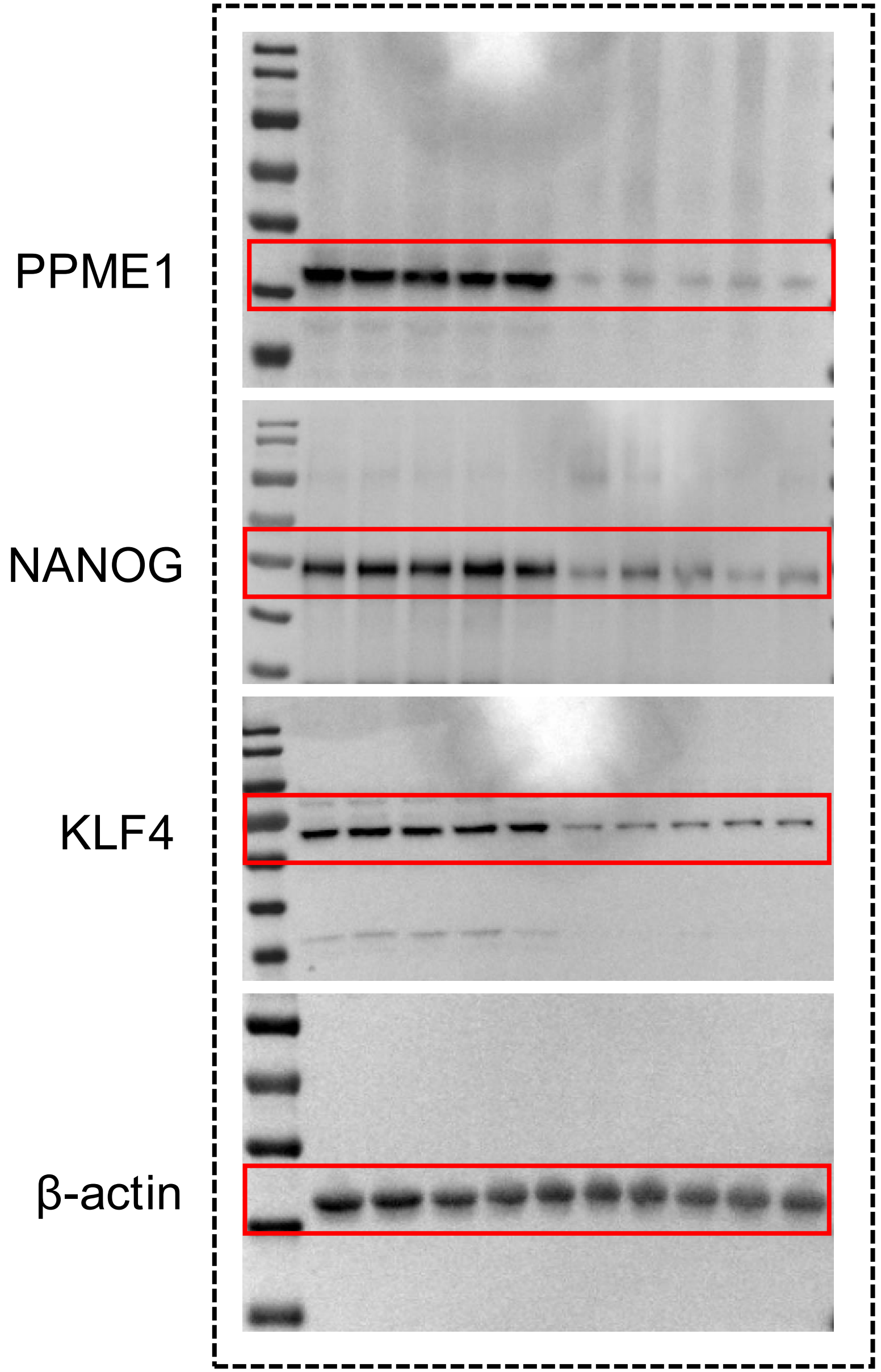

Figure5

Full unedited blot/gel for Figure5C

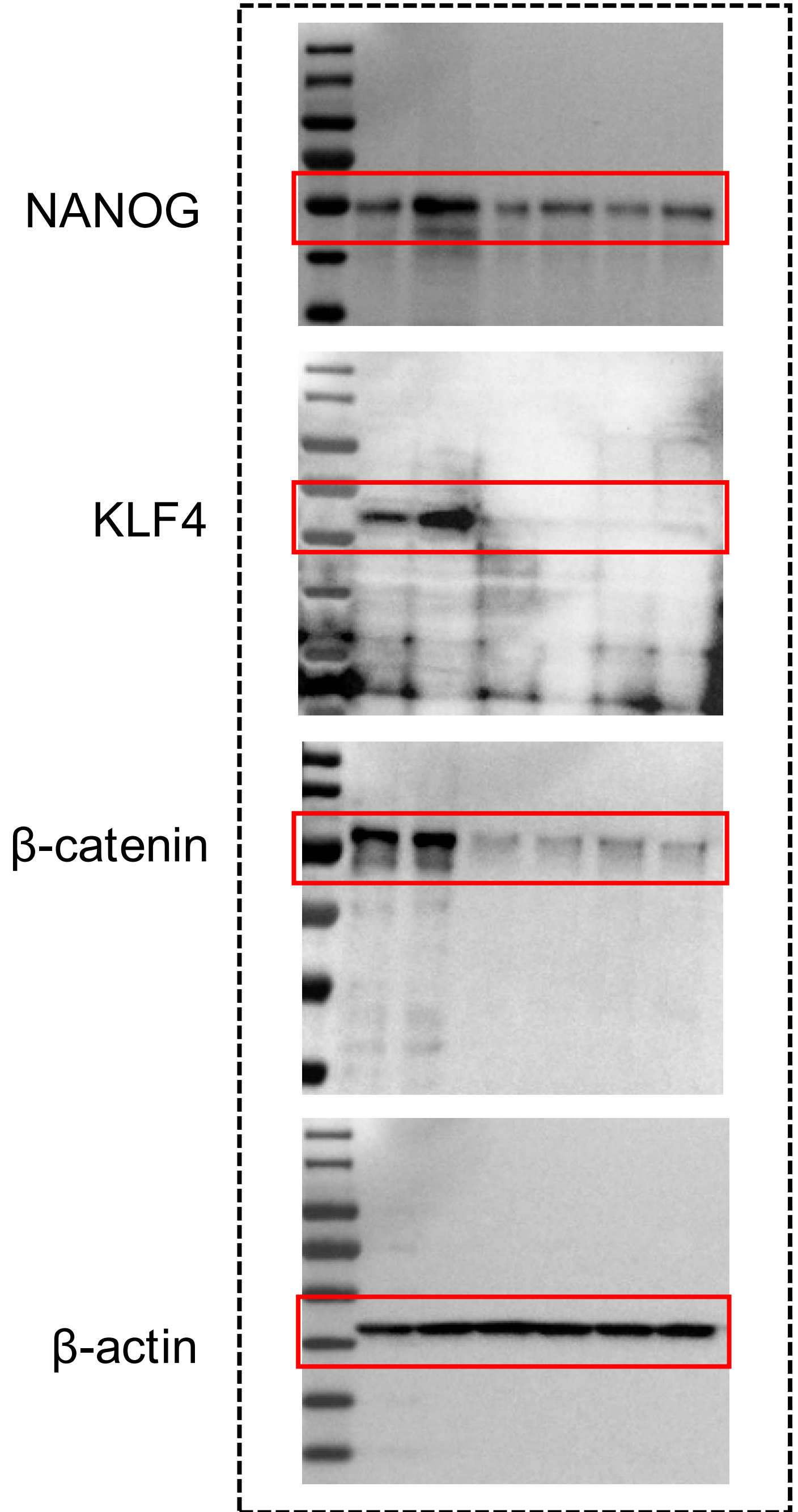

Figure7

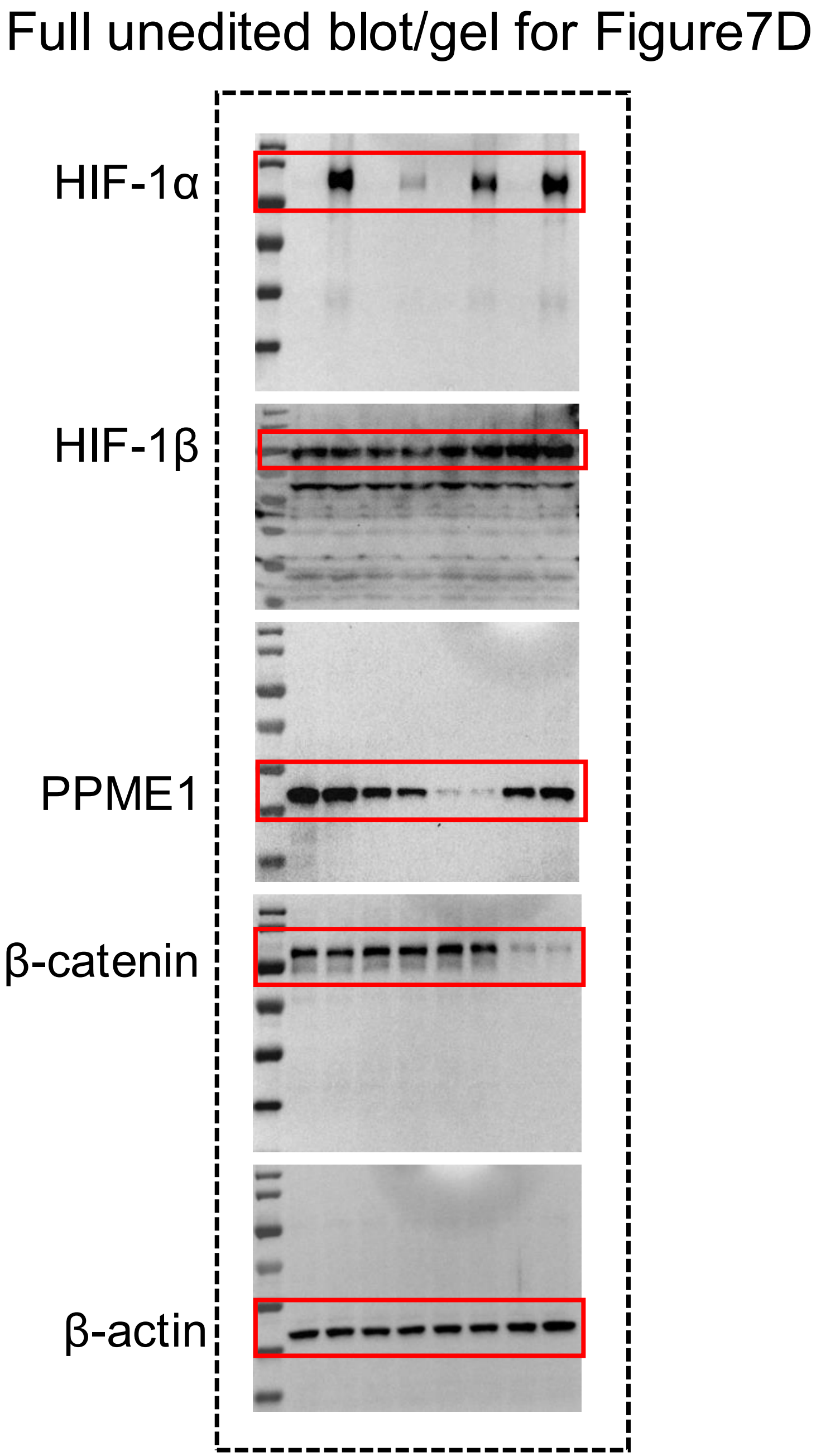
Full unedited blot/gel for Figure7D
HIF-1α
HIF-1β
PPME1
β-catenin
β-actin

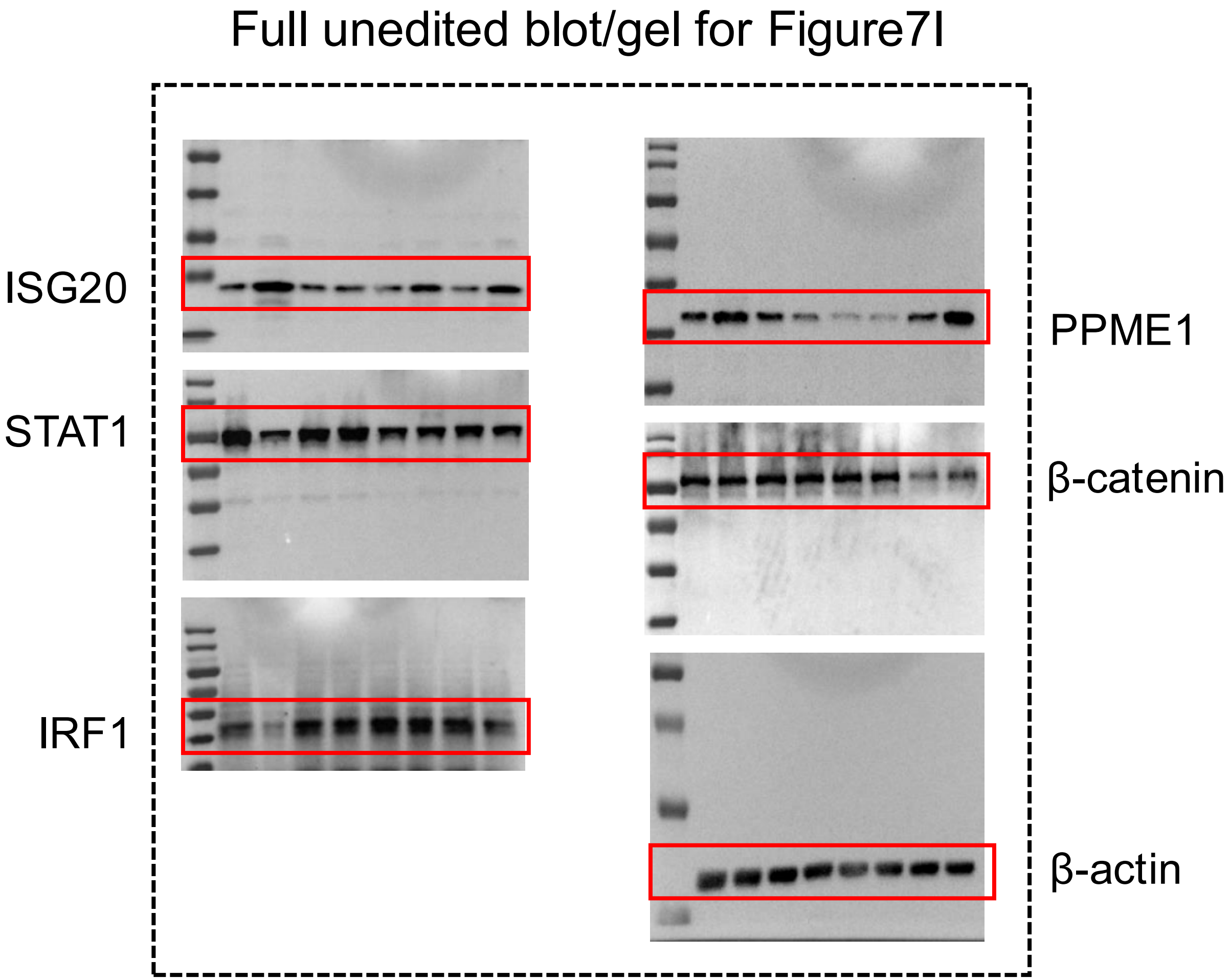
Full unedited blot/gel for Figure7I
ISG20
STAT1
IRF1
PPME1
β-catenin
β-actin

Figure8

Full unedited blot/gel for Figure8M

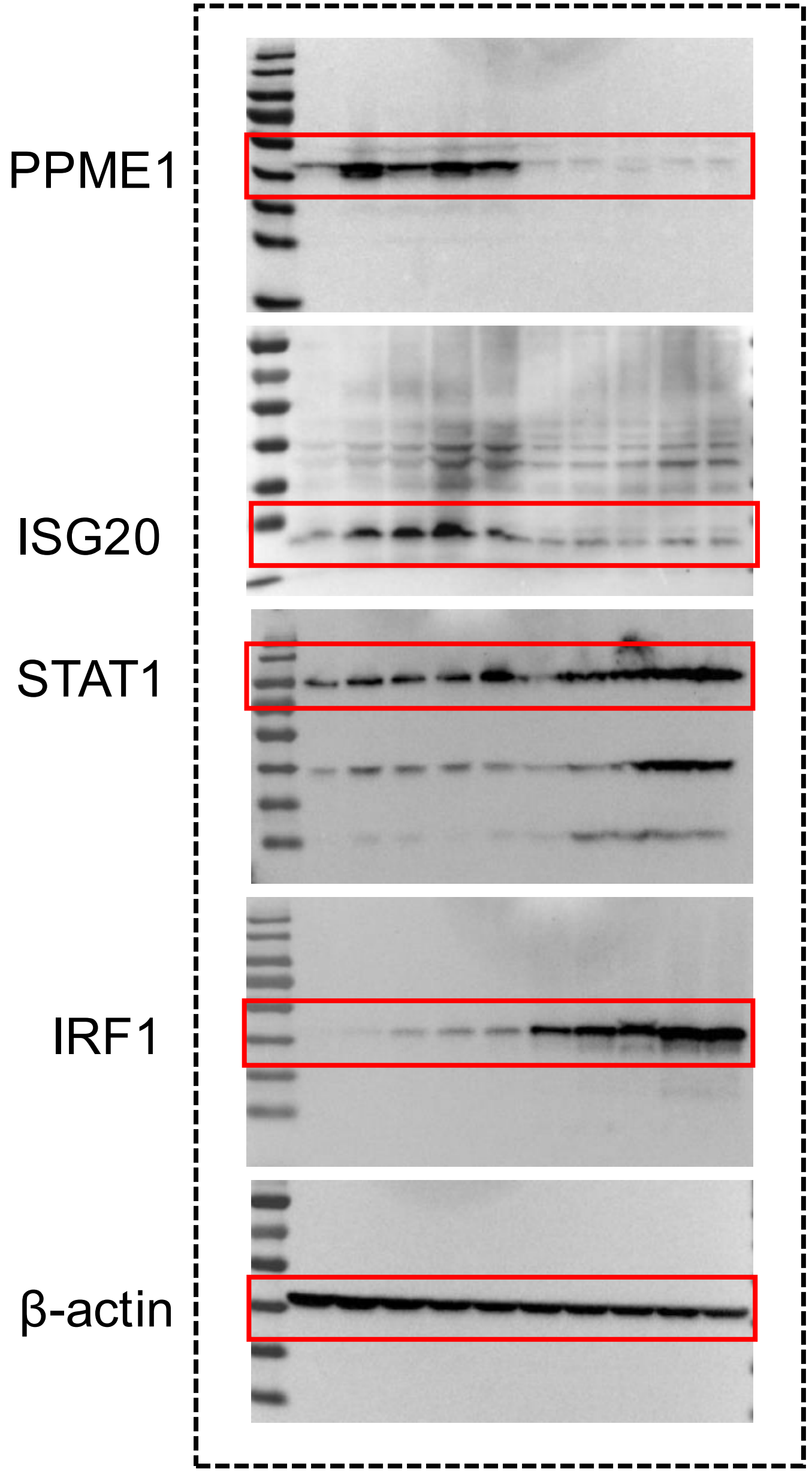

Figure S1

Full unedited blot/gel for Figure S1B

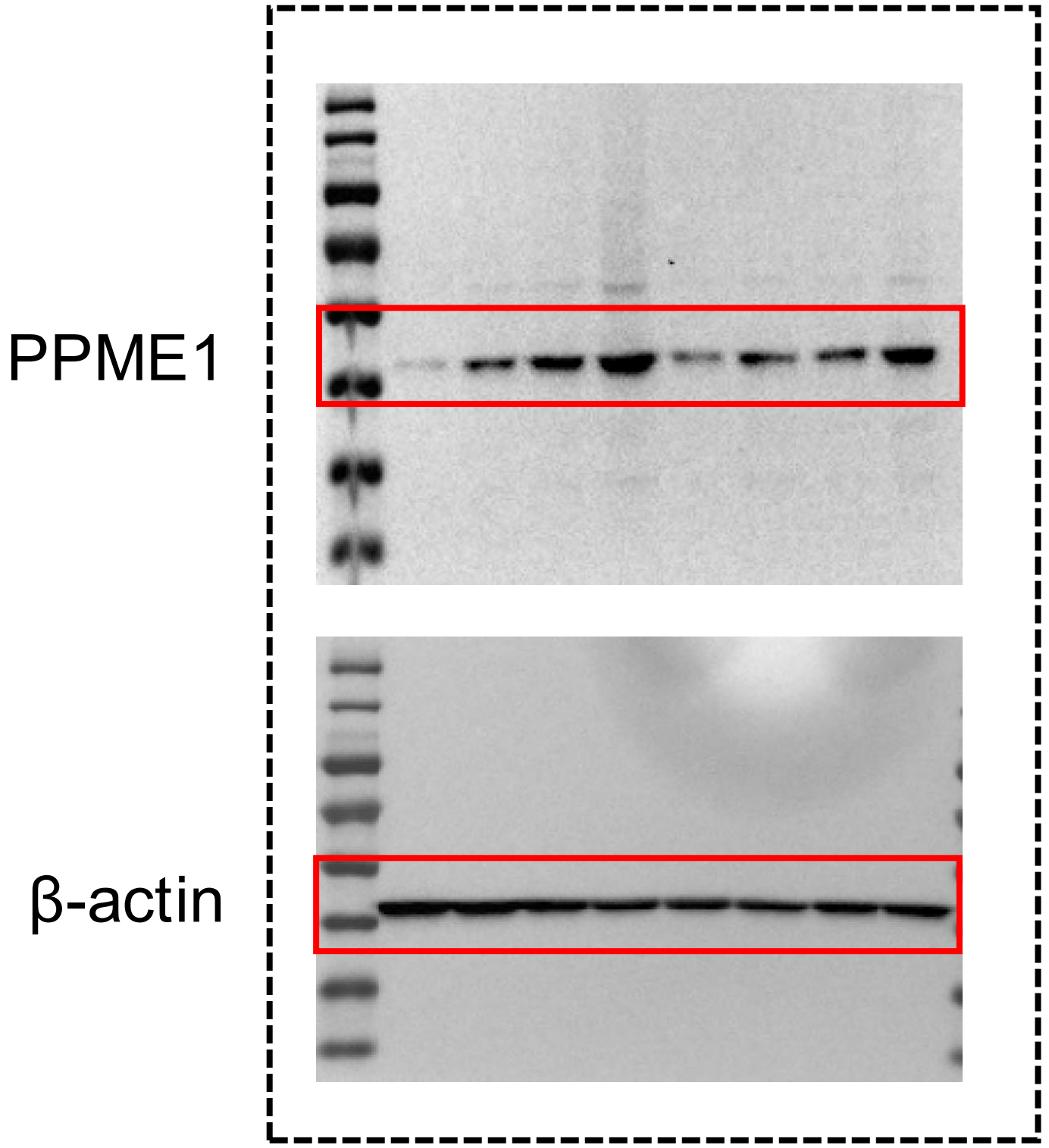

Full unedited blot/gel for Figure S1E

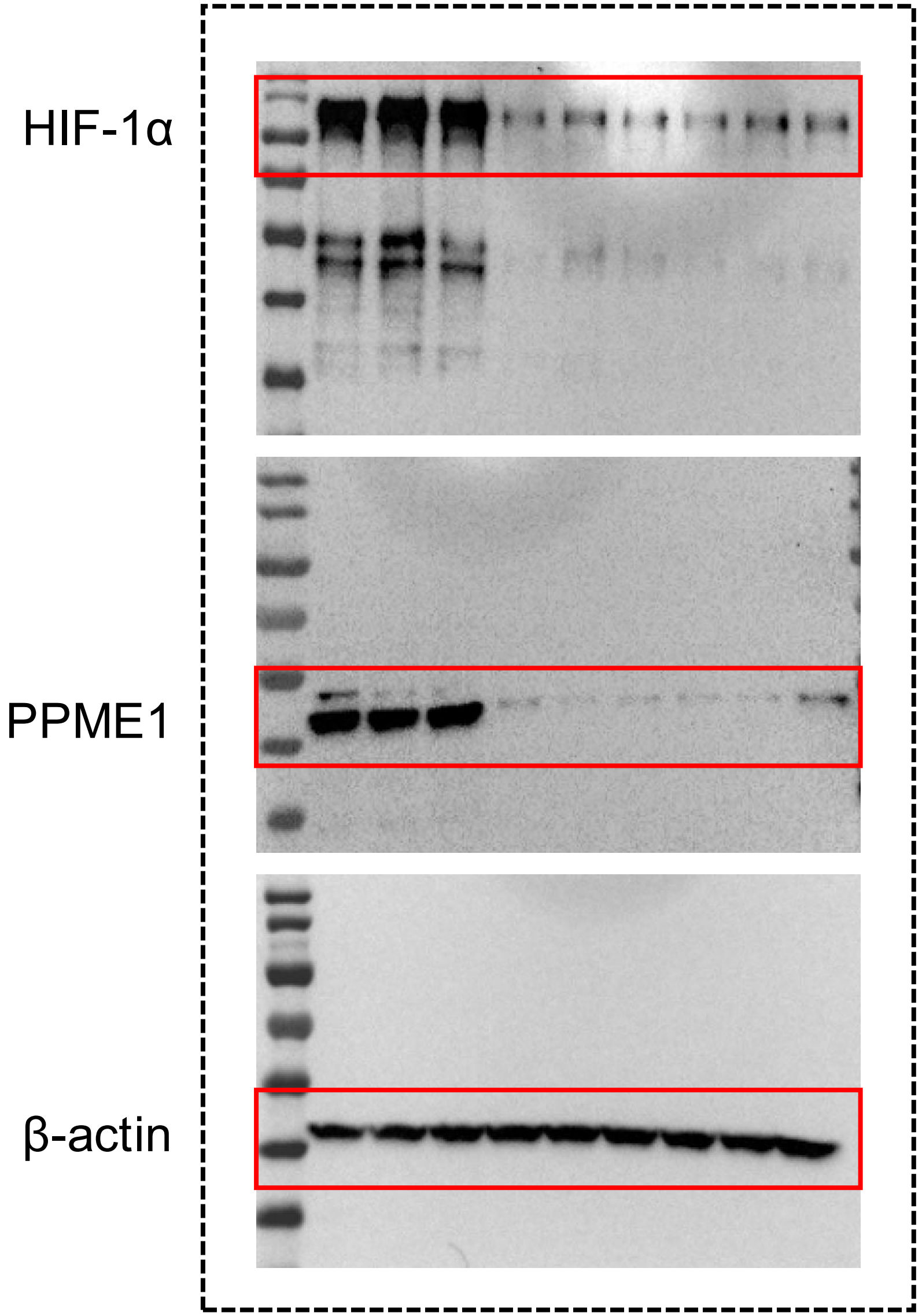

Figure S2

Full unedited blot/gel for Figure S2A

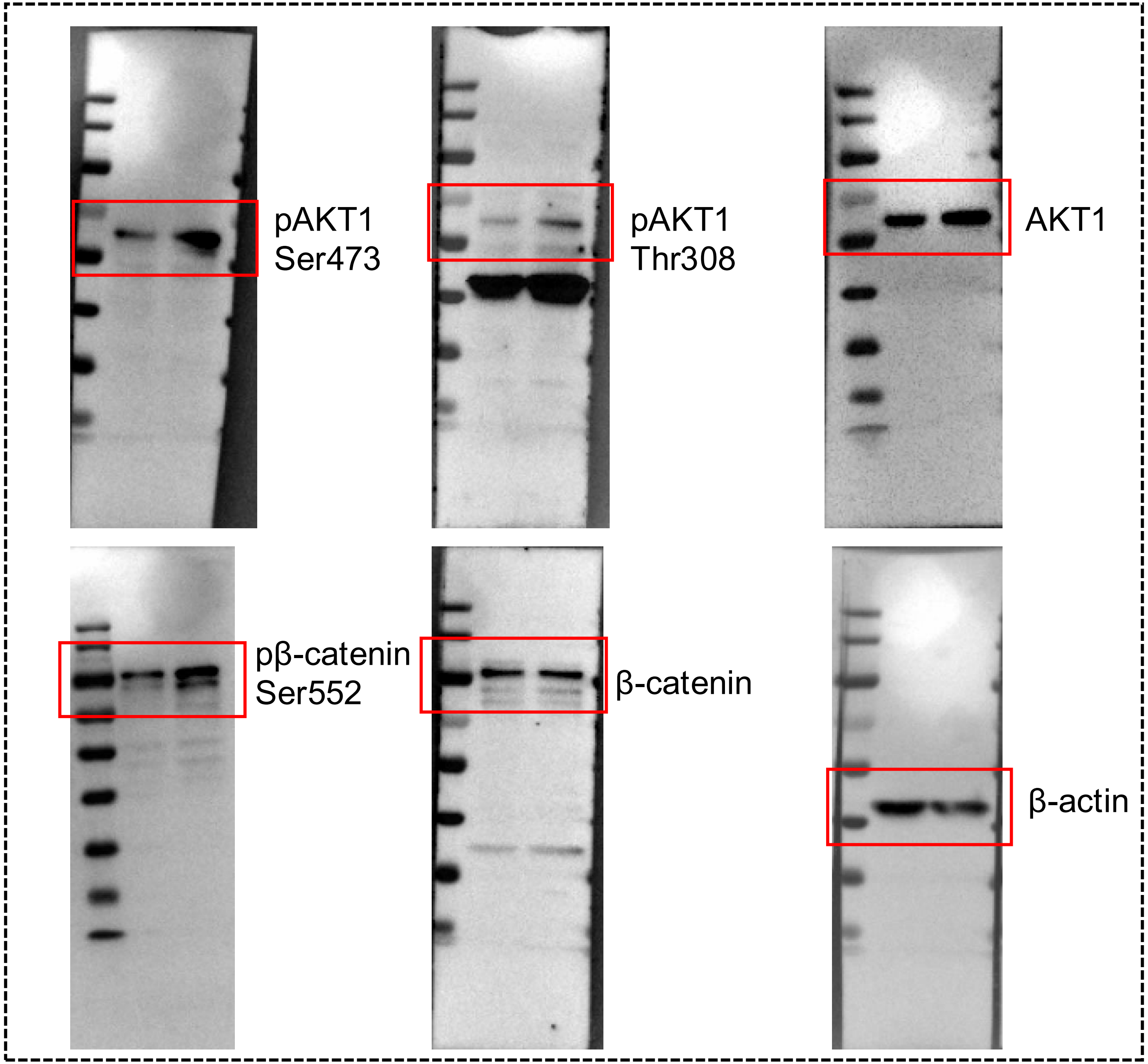

Figure S2

Full unedited blot/gel for Figure S2C

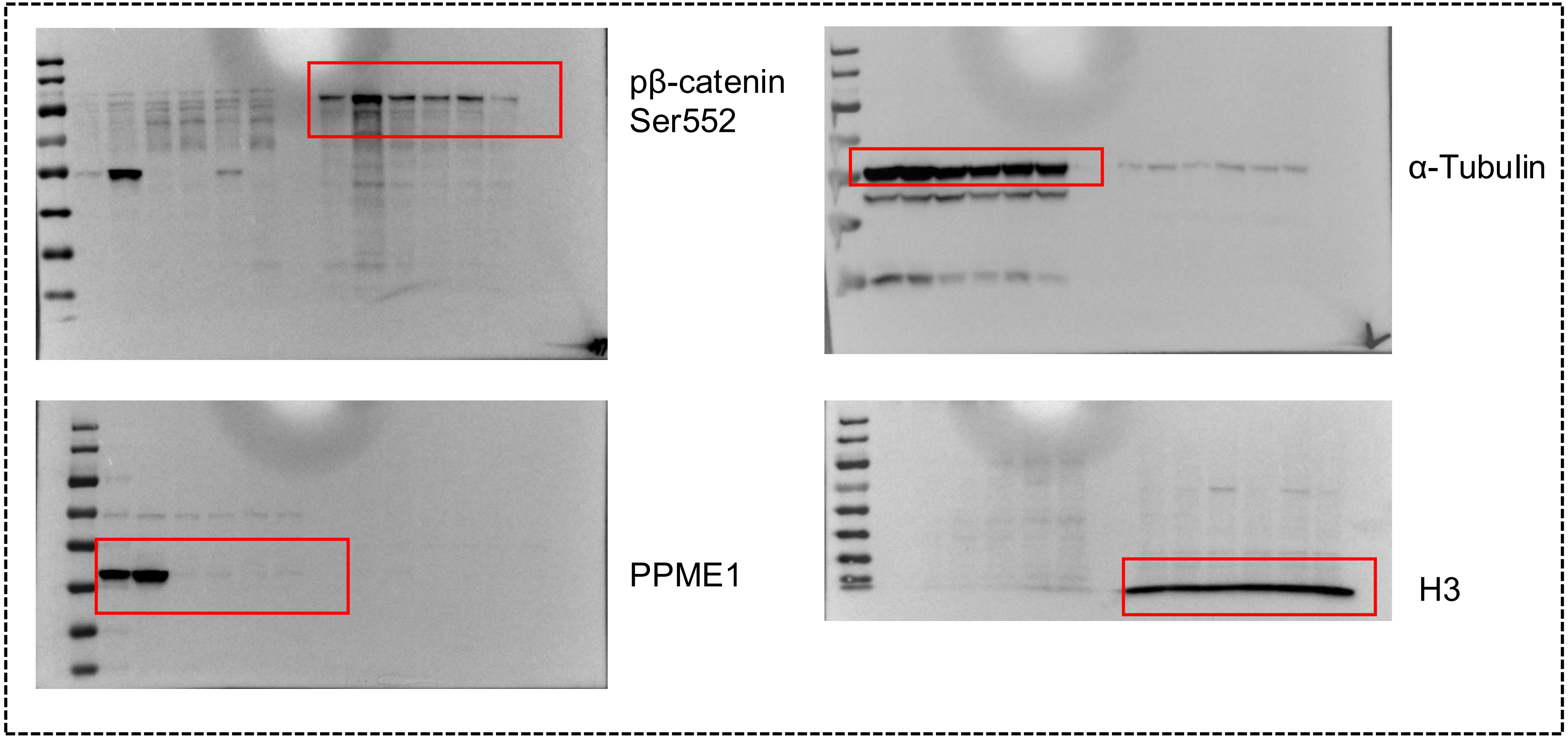

Figure S2

Full unedited blot/gel for Figure S2D

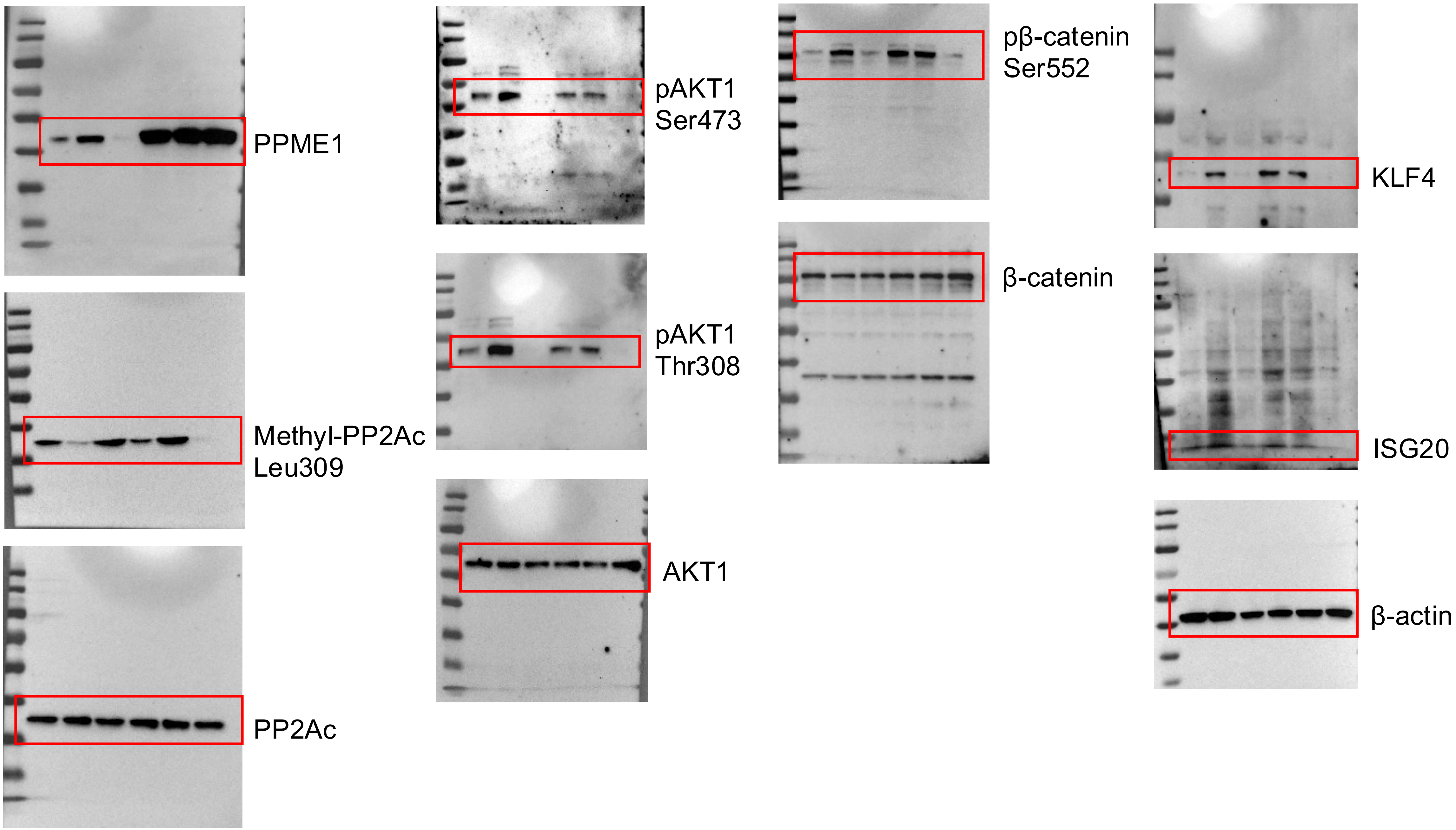

Figure S3

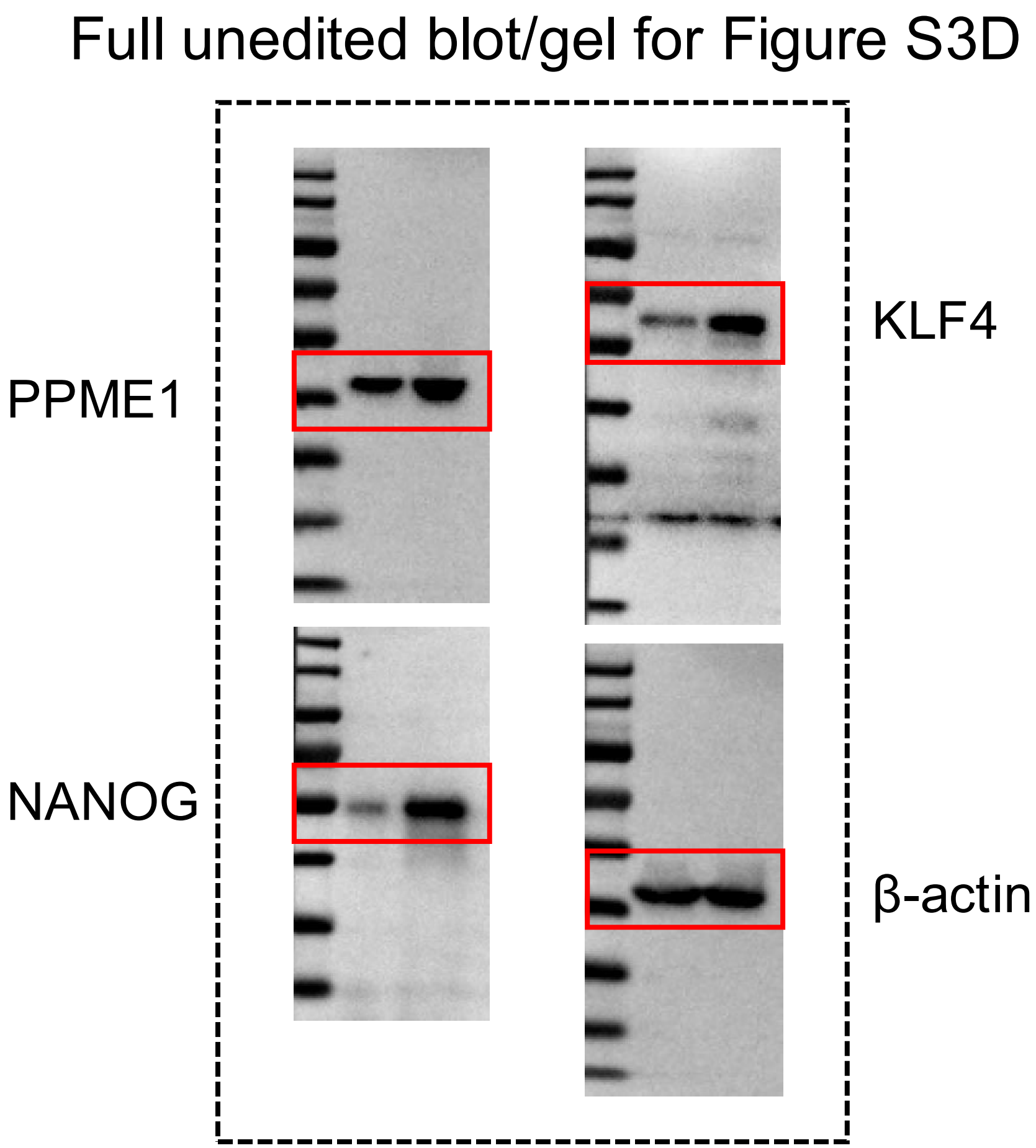

Full unedited blot/gel for Figure S3D
PPME1
NANOG
KLF4
β-actin

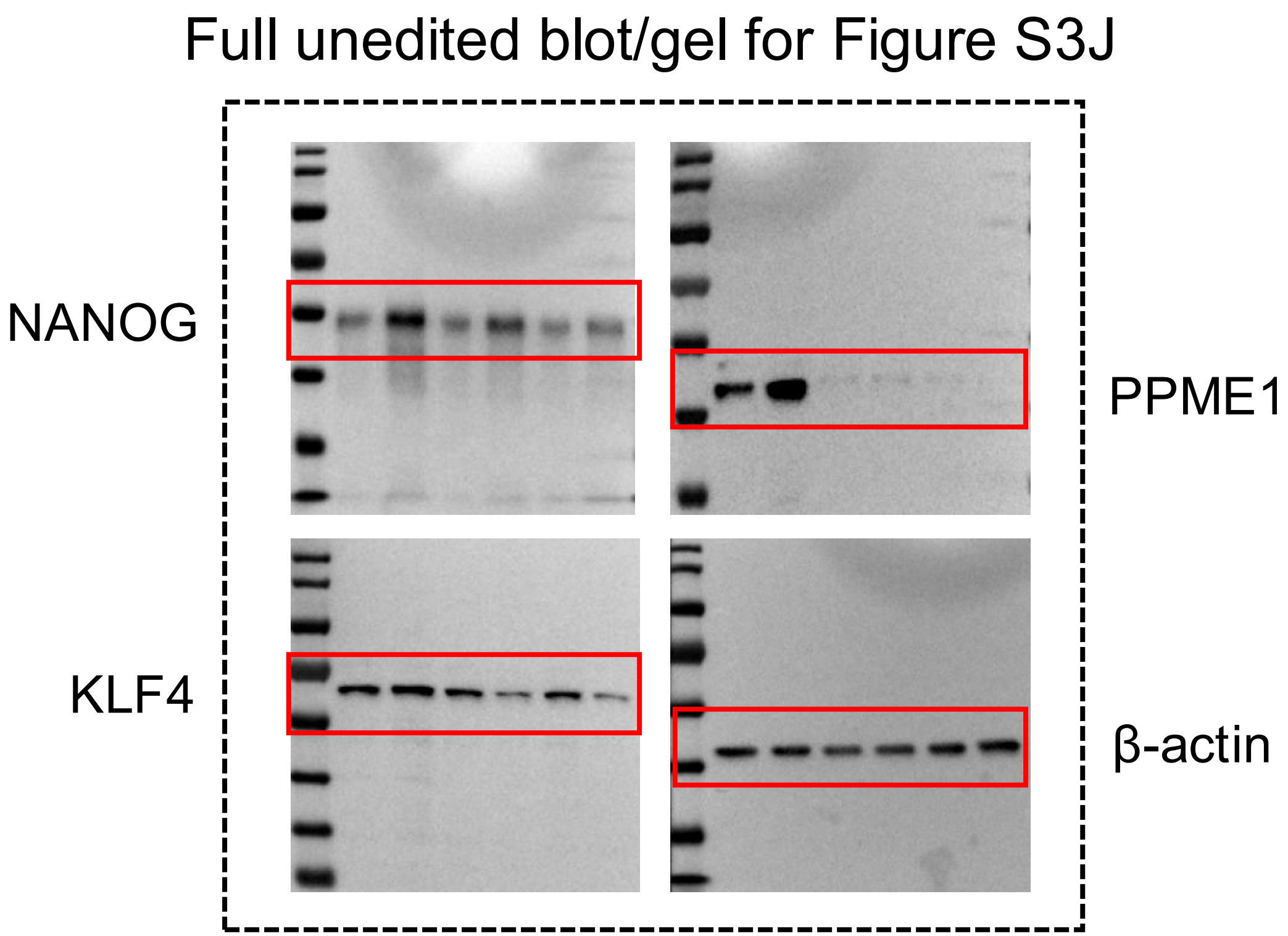

Full unedited blot/gel for Figure S3J
NANOG
KLF4
PPME1
β-actin

Figure S5

Full unedited blot/gel for Figure S5C

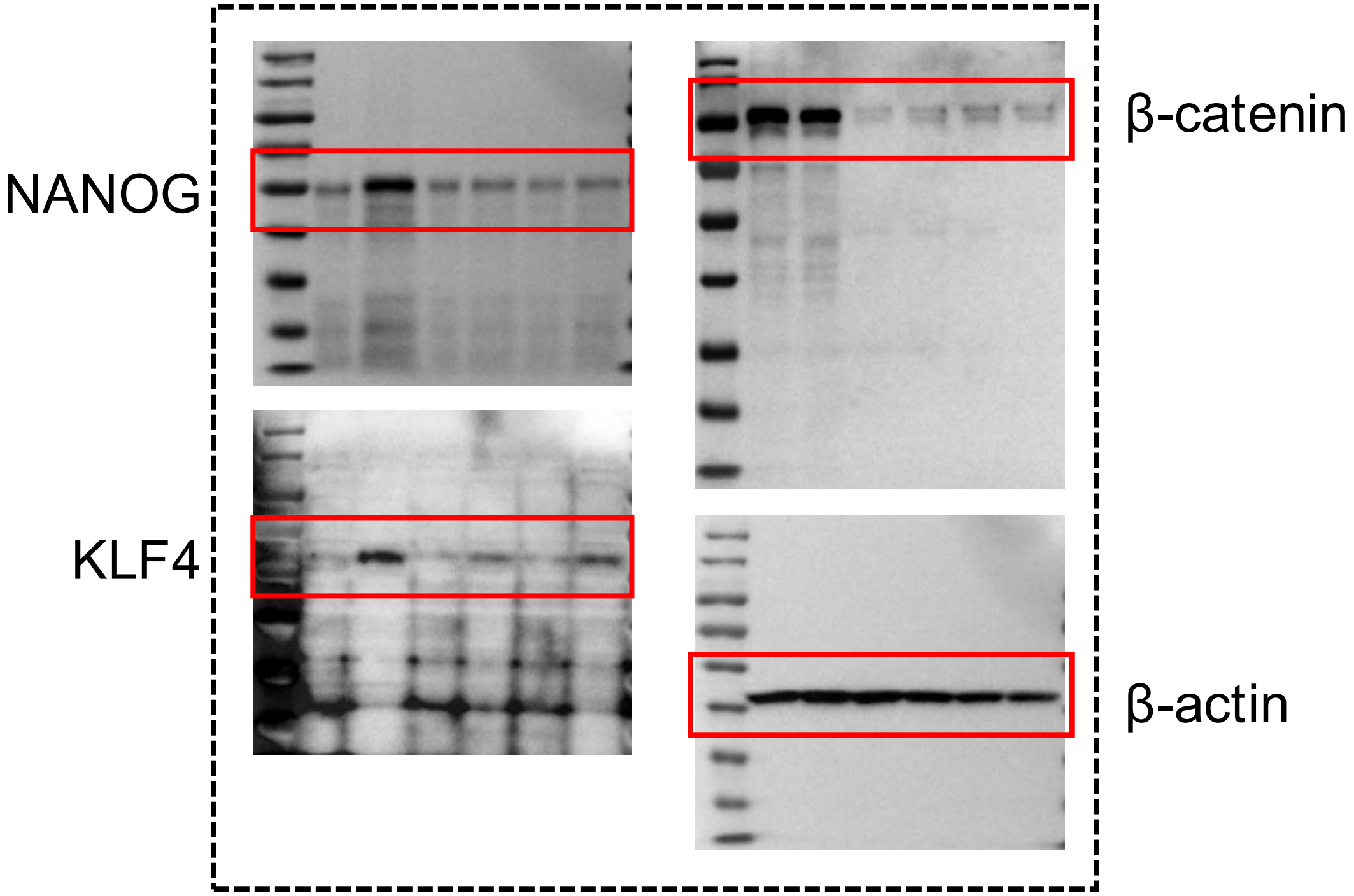

Full unedited blot/gel for Figure S5D

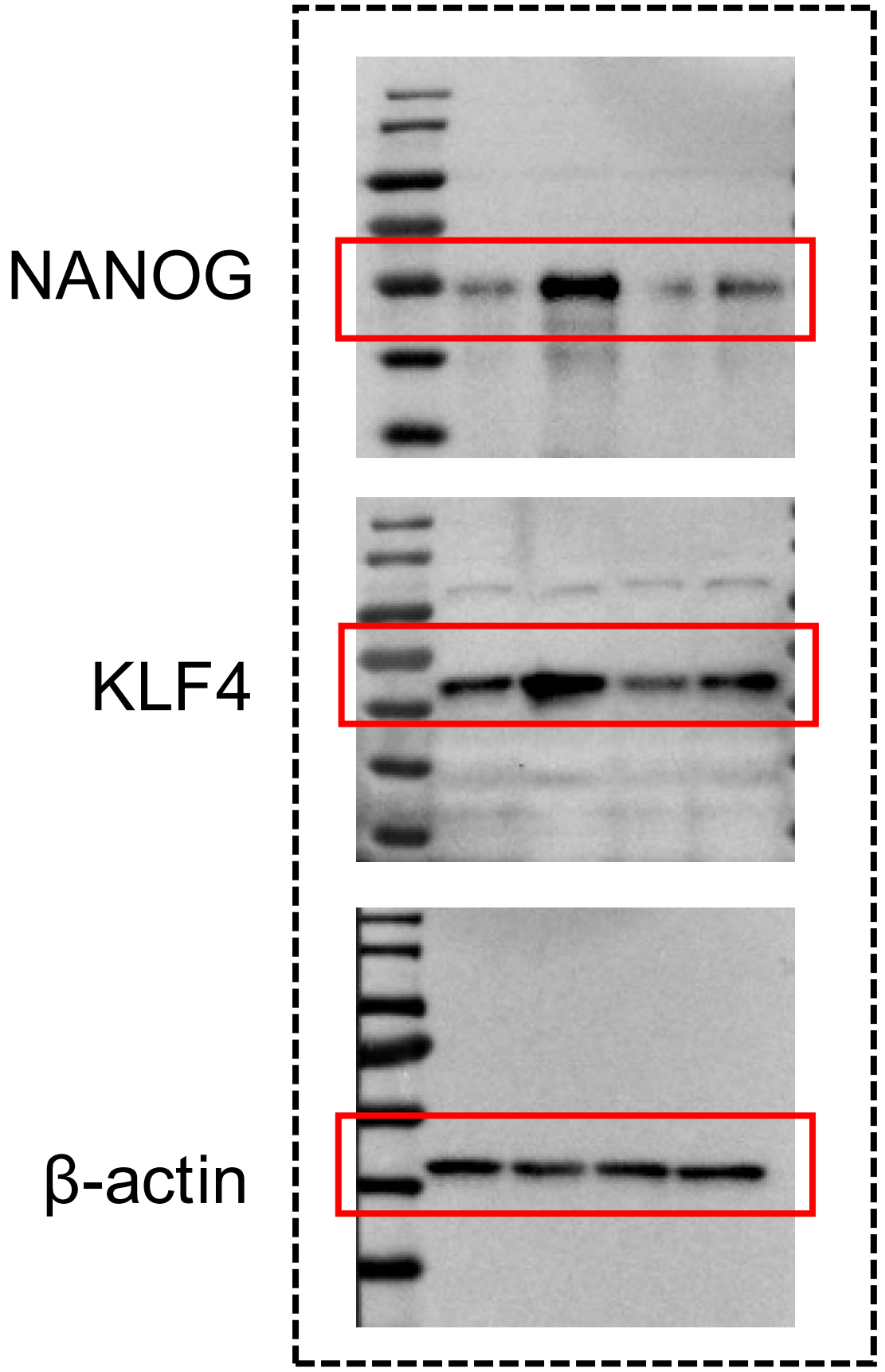

Full unedited blot/gel for Figure S5E

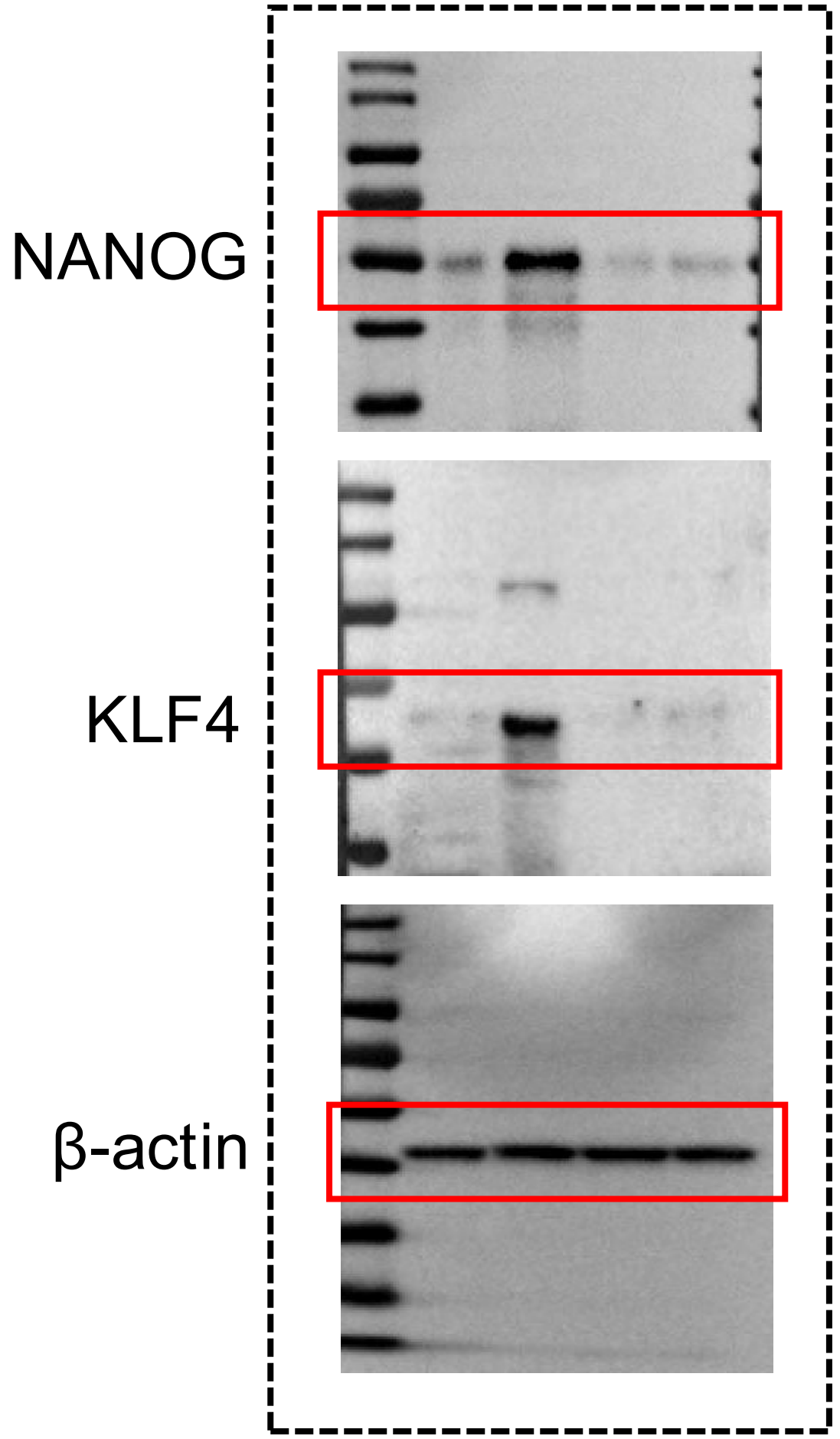

Figure S10

Full unedited blot/gel for Figure S10A

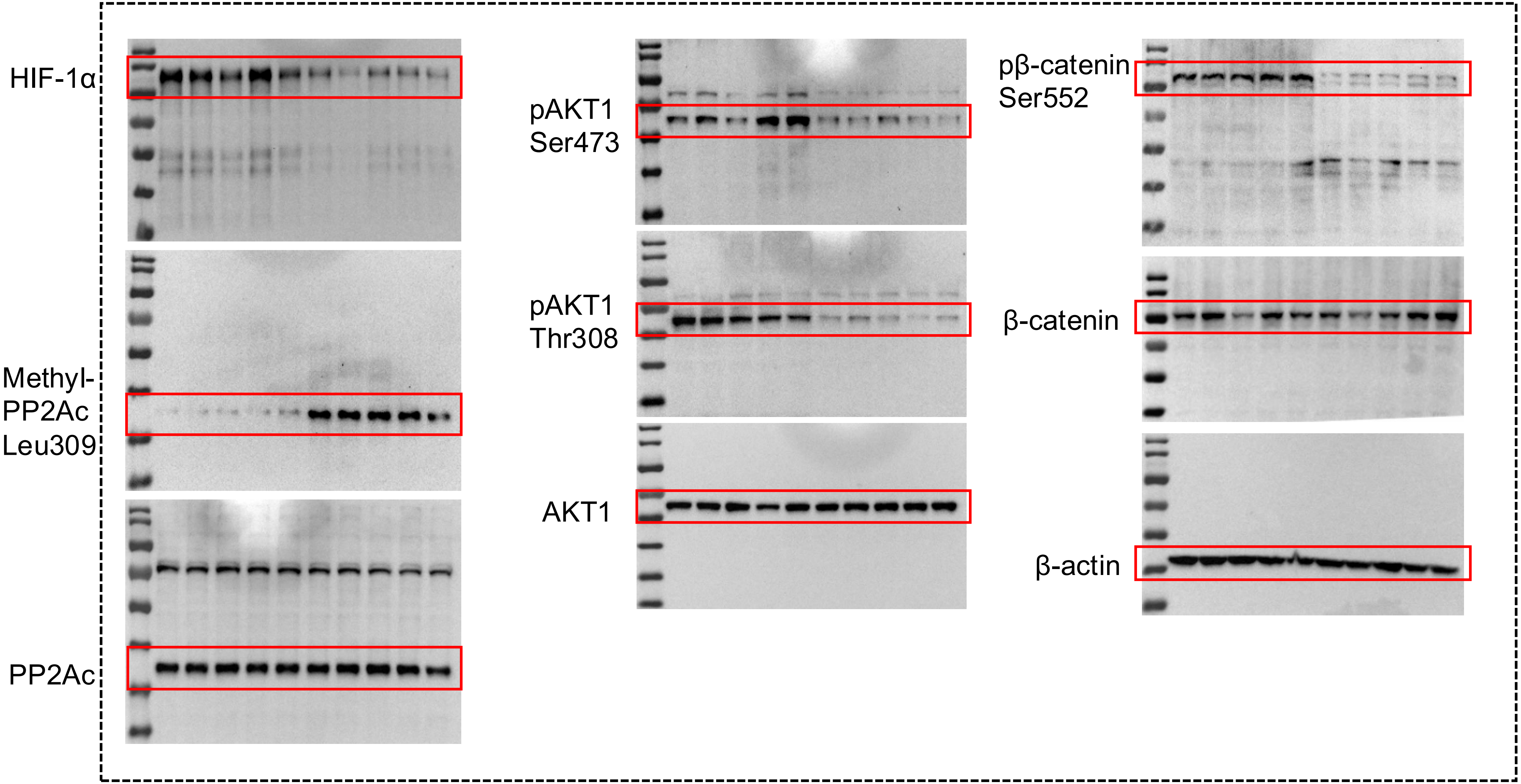

Supplement: Supplementary file 2 [file jci-136-205415-s085.pdf]
